# Multigenerational cell tracking of DNA replication and heritable DNA damage

**DOI:** 10.1038/s41586-025-08986-0

**Published:** 2025-05-21

**Authors:** Andreas Panagopoulos, Merula Stout, Sinan Kilic, Peter Leary, Julia Vornberger, Virginia Pasti, Antonio Galarreta, Aleksandra Lezaja, Kyra Kirschenbühler, Ralph Imhof, Hubert Rehrauer, Urs Ziegler, Matthias Altmeyer

**Affiliations:** 1 Department of Molecular Mechanisms of Disease, University of Zurich, Zurich, Switzerland; 2 Functional Genomics Center Zurich, ETH Zurich and University of Zurich, Zurich, Switzerland; 3 Center for Microscopy and Image Analysis, University of Zurich, Zurich, Switzerland; 4 Present Address: The Novo Nordisk Foundation Center for Protein Research, University of Copenhagen, Copenhagen, Denmark; 5 Present Address: NEXUS Personalized Health, ETH Zurich, Schlieren, Switzerland

**Keywords:** Cell biology, Cellular imaging, DNA damage and repair, DNA replication, Cell division

## Abstract

Cell heterogeneity is a universal feature of life. Although biological processes affected by cell-to-cell variation are manifold, from developmental plasticity to tumour heterogeneity and differential drug responses, the sources of cell heterogeneity remain largely unclear^1,2^. Mutational and epigenetic signatures from cancer (epi)genomics are powerful for deducing processes that shaped cancer genome evolution^3–5^. However, retrospective analyses face difficulties in resolving how cellular heterogeneity emerges and is propagated to subsequent cell generations. Here, we used multigenerational single-cell tracking based on endogenously labelled proteins and custom-designed computational tools to elucidate how oncogenic perturbations induce sister cell asymmetry and phenotypic heterogeneity. Dual CRISPR-based genome editing enabled simultaneous tracking of DNA replication patterns and heritable endogenous DNA lesions. Cell lineage trees of up to four generations were tracked in asynchronously growing cells, and time-resolved lineage analyses were combined with end-point measurements of cell cycle and DNA damage markers through iterative staining. Besides revealing replication and repair dynamics, damage inheritance and emergence of sister cell heterogeneity across multiple cell generations, through combination with single-cell transcriptomics, we delineate how common oncogenic events trigger multiple routes towards polyploidization with distinct outcomes for genome integrity. Our study provides a framework to dissect phenotypic plasticity at the single-cell level and sheds light onto cellular processes that may resemble early events during cancer development.

## Abstract (web summary)

A multigenerational single-cell tracking approach provides a framework to dissect phenotypic plasticity at the single-cell level, offering insights into cellular processes that may resemble early events during cancer development.

## Main

Cellular heterogeneity, including genetic and non-genetic heterogeneity, is pervasive in nature, yet insufficiently represented in ensemble behaviours of cell populations^6–8^. Stochastic processes in cells, such as the ones involved in transcription, translation, protein turnover and cell division, cause fluctuations in cellular components and in biochemical reactions that can drive phenotypic variability. Such stochastic variation may be buffered or amplified by deterministic factors, most of which are unknown^9,10^.

Phenotypic plasticity is a hallmark of cancer, and most tumours show genetic and non-genetic spatial and temporal heterogeneity, which affects adaptability and resistance to cancer therapies^11,12^. Classical Darwinian somatic evolution driven by mutations, selection and clonal expansion appears to be insufficient to fully explain cancer progression and responses to therapies^13,14^. Moreover, genomic lesions that shape cancer genome evolution and adaptation are typically not resolved within a single cell cycle but, instead, segregate into subsequent cell generations^15,16^. How genetic and non-genetic heterogeneity are intertwined and the dynamics with which phenotypic heterogeneity emerges and is propagated across cell generations are poorly understood.

Polyploidization by whole-genome duplication occurs frequently in cancer and is associated with enhanced phenotypic variability^17,18^. Polyploidization can lead to chromosomal instability and aneuploidy^19–21^, which correlate with therapy resistance and poor patient outcomes^22,23^. Aneuploidy can arise through erroneous cell division, and asymmetric cell divisions promote cell-to-cell variability, including both genetic and non-genetic heterogeneity^24,25^. Alternative routes to polyploidization exist, but how they affect genome integrity and cellular variability between cancer cells remains unclear.

A main driver of carcinogenesis is oncogene-induced replication stress^26^. Endogenous and oncogene-induced replication stress give rise to heritable DNA lesions that are transmitted through cell division to daughter cells^27^. Such heritable lesions may be an inevitable consequence of stochastic replication origin activation, resulting in under-replicated DNA in large replicons of the human genome^28^. In daughter cells, inherited genomic lesions regulate G1 duration and determine the decision between quiescence and cell cycle commitment^29–33^. They are bound by the chromatin reader 53BP1, which protects them from nucleolytic degradation^27^ and regulates their replication timing in the next S phase^34^. While representing a paradigm for inheritance of genomic lesions, whether 53BP1-marked chromatin scars affect sister cell heterogeneity and are propagated to subsequent cell generations is unclear.

Here, to overcome limitations of cell tracking and complement genomics-focused retrospective analyses of cancer cell evolution, we devised an approach for quantitative multigenerational single-cell tracking and lineage analysis. The approach is based on endogenous protein labelling through CRISPR–Cas9-mediated genome editing, tailored cell segmentation and tracking algorithms, and single-cell end-point measurements by iterative staining. Applied to various conditions of oncogenic replication stress and DNA damage, it provides time-resolved insights into damage inheritance and emergence of sister cell asymmetry, and sheds light on routes towards polyploidization and associated phenotypic variability that may contribute to cancer evolution.

## Tracking replication and DNA damage

**Fig. 1:**
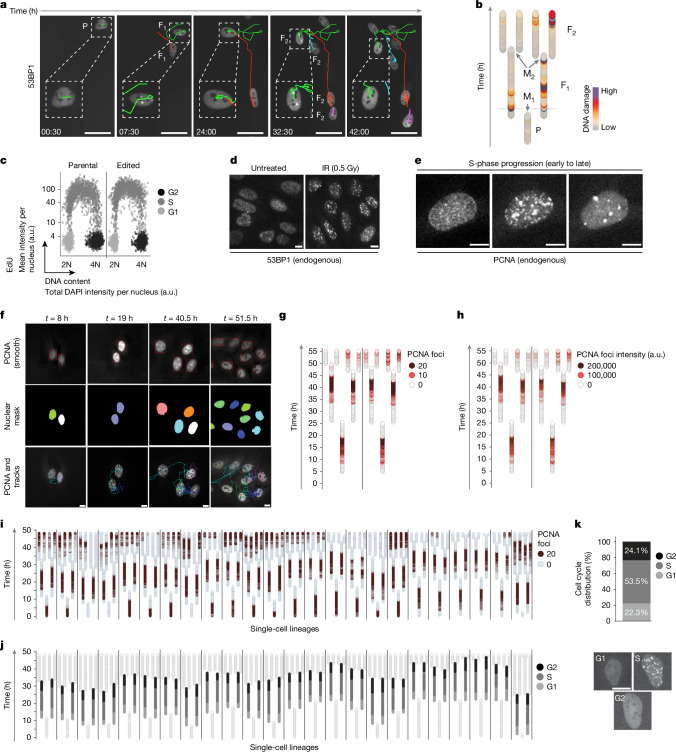
Quantitative cell-cycle-resolved single-cell tracking based on endogenous markers. **a**, Representative images from time-lapse microscopy with multigenerational cell tracking of mEGFP–53BP1 U-2 OS cells. The daughter cell generation (F_1_) and granddaughter cell generation (F_2_) generations with 53BP1 foci after cell division are highlighted. Scale bars, 50 µm. **b**, Example cell lineage depicting lineage relationships and DNA damage marked by 53BP1. P, parental cell generation; M_1_, first mitosis; M_2_, second mitosis. Time-lapse microscopy for 65 h at 30 min intervals. **c**, High-content-microscopy-derived EdU profiles of the parental and the edited 53BP1–PCNA U-2 OS cell line. a.u., arbitrary units. **d**, Representative images of endogenously tagged 53BP1 from 53BP1–PCNA U-2 OS cells treated as indicated and fixed after 45 min. Scale bars, 10 µm. **e**, Representative images from 53BP1–PCNA U-2 OS cells depicting endogenous PCNA patterns during S phase. Scale bars, 10 µm. **f**, Representative images of the cell-tracking procedure, depicting the smoothened endogenous PCNA signal for nuclei segmentation, the nuclear mask and the single-cell tracks applied on the non-smoothened PCNA signal. Scale bars, 10 µm. **g**, Single-cell lineages of the cells depicted in **f**. Endogenous PCNA patterns are depicted according to the colour code indicated. **h**, The same single-cell lineages as in **g**, with endogenous PCNA foci intensities depicted according to the colour code indicated. **i**, Single-cell lineages from endogenously tagged 53BP1–PCNA U-2 OS cells. PCNA foci are depicted according to the colour code indicated. **j**, The same single-cell lineages as in **i**, with cell cycle phase of the F_1_ generation, based on the endogenous PCNA signals, depicted according to the colour code indicated (G1, S, G2). **k**, The cell cycle distribution derived from the time spent in G1, S and G2 according to the endogenous PCNA foci pattern of the single-cell lineages shown in **i** and **j**. Representative single-cell images are shown below. Scale bar, 10 µm.

**Extended Data Fig. 1:**
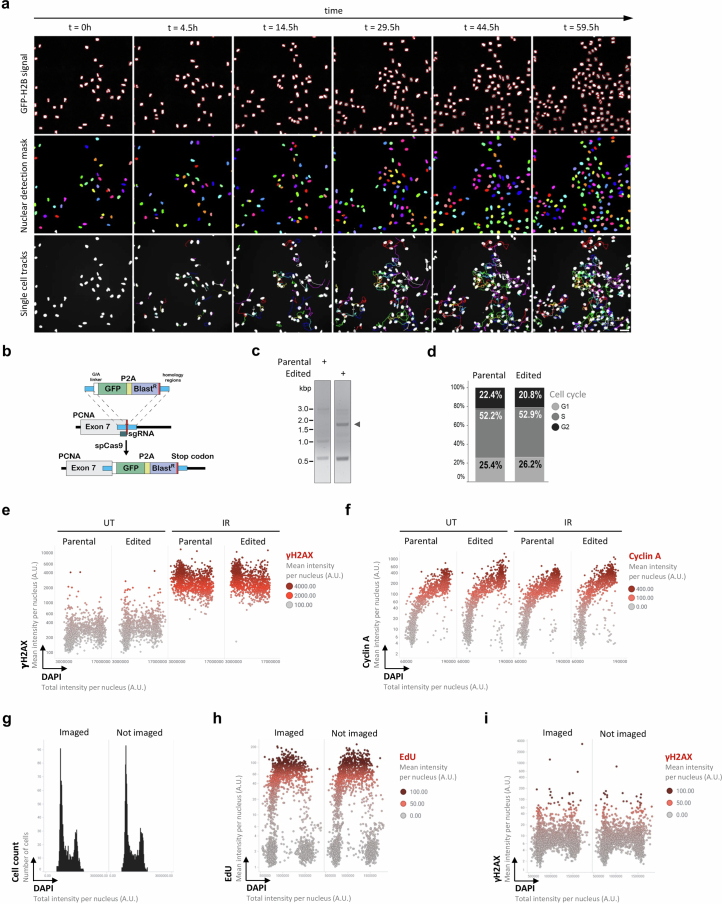
CRISPR-engineered cell lines and multigenerational cell tracking. (**a**) Representative live imaging stills with the nuclear detection mask and the multigenerational single cell tracks from GFP-H2B U-2 OS cells. Scale bar, 50 µm. (**b**) Schematic representation of the strategy for tagging the endogenous *PCNA* gene locus at the last exon in U-2 OS cells. (**c**) Gel electrophoresis of the PCR products of the *PCNA* locus from the parental and the edited U-2 OS cell line. The arrowhead points to the band indicative of correct targeting. (**d**) Cell cycle phase distribution of the parental and the edited U-2 OS cell line based on DAPI and EdU. (**e**) Cell cycle resolved γH2AX mean intensity scatter plots of the parental and the edited 53BP1-PCNA U-2 OS cells either untreated or IR-treated (4 Gy, 2 h recovery). (**f**) Cell cycle resolved Cyclin A mean intensity scatter plots of the parental and the edited 53BP1-PCNA U-2 OS cells either untreated or IR-treated (4 Gy, 2 h recovery). (**g**) High-content microscopy derived cell cycle profiles of 53BP1-PCNA U-2 OS cells subjected or not to timelapse microscopy for 28 h at 30 min intervals. (**h**) Cell cycle resolved EdU mean intensity scatter plots of 53BP1-PCNA U-2 OS cells subjected or not to timelapse microscopy for 28 h at 30 min intervals. (**i**) Cell cycle resolved γH2AX mean intensity scatter plots of 53BP1-PCNA U-2 OS cells subjected or not to timelapse microscopy for 28 h at 30 min intervals.

To develop an experimental framework enabling simultaneous tracking of individual cells and their fates across multiple cell generations, we first used cells stably expressing ectopic H2B–GFP. The bright nuclear H2B–GFP signal facilitated software-assisted image segmentation, enabling us to define conditions for multi-day live-cell imaging and design custom scripts for semi-automated single-cell tracking (Methods and Extended Data Fig. 1a). To test whether multigenerational single-cell tracking could be combined with tracking DNA lesions in individual cells, we turned to cells stably expressing ectopic mEGFP–53BP1. The nuclear mEGFP–53BP1 signal was bright enough for automated image segmentation and cell tracking (Fig. 1a), and the appearance and disappearance of nuclear 53BP1 bodies resembling replication-stress-associated DNA lesions could be quantified in a time-resolved manner in the daughter and granddaughter cell generation and could be depicted as cell lineage trees (Fig. 1b).

**Extended Data Fig. 2:**
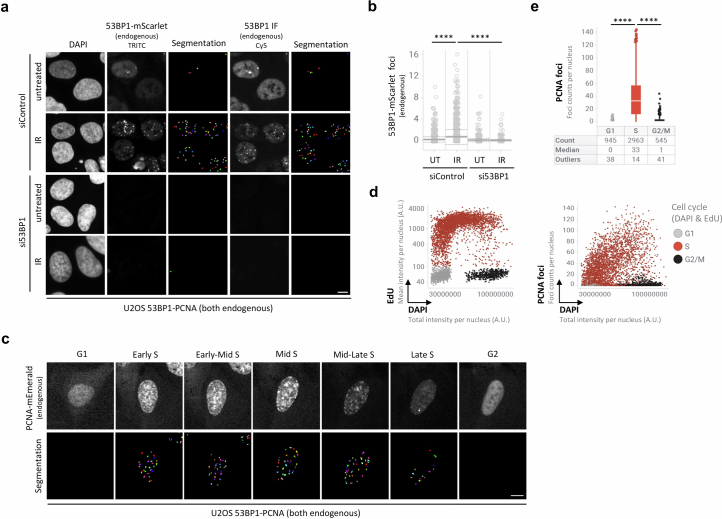
Validation of CRISPR-engineered 53BP1-PCNA U-2 OS cells. (**a**) 53BP1-PCNA U-2 OS cells were treated with siRNA for 72 h and IR (4 Gy, 2 h) as indicated and stained for 53BP1 (Cy5). The endogenous 53BP1-mScarlet and antibody-based signals are shown, together with their segmentation masks. Scale bar, 10 µm. (**b**) High-content microscopy-based quantification of 53BP1-mScarlet foci using the samples and foci segmentation mask from (a). Statistical analysis by one-way ANOVA followed by Tukey’s post hoc test. (**c**) Video stills of 53BP1-PCNA U-2 OS cells at different cell cycle phases from G1 through S-phase and to G2. PCNA-mEmerald patterns and their segmentation are shown. Scale bar, 10 µm. (**d**) DAPI- and EdU-based cell cycle gating of 53BP1-PCNA U-2 OS cells and PCNA foci analysis in different cell cycle phases (colour-coded based on DAPI and EdU). (**e**) PCNA foci quantification from (d) in G1 versus S-phase versus G2 (based on DAPI and EdU). Statistical analysis by one-way ANOVA followed by Tukey’s post hoc test. Source data

Having established that endogenous DNA lesions marked by 53BP1 can be tracked quantitatively across multiple cell generations, we aimed to overcome limitations associated with ectopic protein overexpression by using CRISPR–Cas9-engineered cells containing the coding sequence for mScarlet-I in-frame with the last exon of *TP53BP1*^33^. To combine time-resolved measurements of heritable DNA lesions with DNA replication patterns, we also tagged the replication factor PCNA by CRISPR–Cas9-mediated knock-in of *mEmerald* in frame with the last exon (Extended Data Fig. 1b,c). The engineered double-tagged cells showed normal proliferation, unperturbed cell cycle profiles and a normal response to DNA damage compared to the parental cells (Fig. 1c and Extended Data Fig. 1d–f). No detectable phototoxicity was observed in time-lapse microscopy experiments, as measured by cell cycle profiling, EdU incorporation and cell-cycle-resolved analysis of the DNA damage marker γH2AX (Extended Data Fig. 1g–i). Like mEGFP–53BP1 cells, endogenous DNA lesions could be measured with sufficient resolution using the endogenous 53BP1 protein signal (Extended Data Fig. 2a) and 53BP1–mScarlet formed nuclear repair condensates at endogenous DNA lesions and at sites of induced DNA damage (Fig. 1d). The endogenous 53BP1 foci detected through the mScarlet signal co-localized with foci from 53BP1 immunofluorescence, and their specificity was validated by *53BP1* knockdown (Extended Data Fig. 2a,b). The endogenous PCNA–mEmerald signal marked regions of active replication in S phase, which were confined to EdU-positive cells (Fig. 1e and Extended Data Fig. 2c–e). Segmentation of the PCNA replication pattern enabled cell cycle staging in single-cell lineages of asynchronously growing cells (Fig. 1f–h), and cell cycle distributions based on PCNA replication patterns from live-cell imaging closely matched cell cycle distributions based on 4′,6-diamidino-2-phenylindole dihydrochloride (DAPI)/EdU profiles from fixed cell populations (Fig. 1i–k and Extended Data Fig. 1d). Moreover, the endogenous PCNA signal in the double-tagged cells could be used for multigenerational cell tracking (Supplementary Video 1).

**Extended Data Fig. 3:**
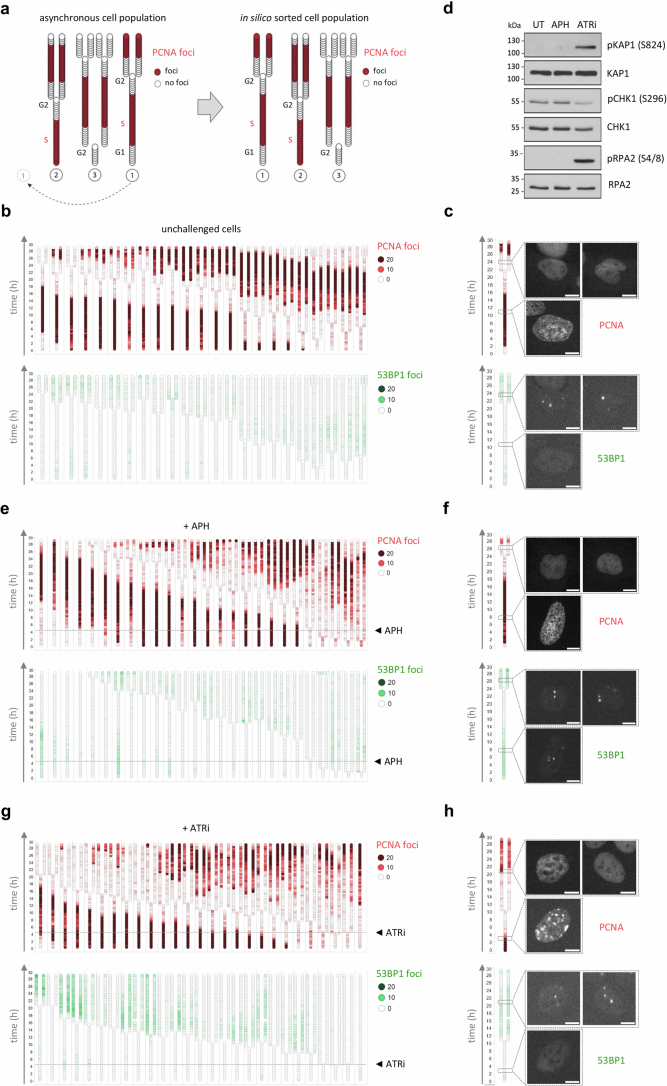
Single cell lineages after replication stress. (**a**) Schematic representation of in silico single cell lineage sorting. (**b***)* In silico sorted single cell lineages from untreated 53BP1-PCNA U-2 OS cells imaged for 30 h. Single cell lineages colour-coded for PCNA patterns (top) and 53BP1 foci (bottom) are shown. (**c**) Representative image stills from a single cell lineage from (b) depicting PCNA patterns and 53BP1 localization at the timepoints indicated. Scale bars, 10 µm. (**d**) Western blot analysis of DNA damage markers in untreated U-2 OS cells or U-2 OS cells treated with 0.2 μM APH or 1 μM ATRi for 24 h. (**e**) In silico sorted single cell lineages from 53BP1-PCNA U-2 OS cells imaged for 30 h. Cells were treated with 0.2 μM APH 4.5 h after the beginning of acquisition. Single cell lineages colour-coded for PCNA patterns (top) and 53BP1 foci (bottom) are shown. (**f**) Representative image stills from a single cell lineage from (e) depicting PCNA patterns and 53BP1 localization at the timepoints indicated. Scale bars, 10 µm. (**g**) In silico sorted single cell lineages from 53BP1-PCNA U-2 OS cells imaged for 30 h. Cells were treated with 1 μM ATRi 4.5 h after the beginning of acquisition. Single cell lineages colour-coded for PCNA patterns (top) and 53BP1 foci (bottom) are shown. (**h**) Representative image stills from a single cell lineage from (g) depicting PCNA patterns and 53BP1 localization at the timepoints indicated. Scale bars, 10 µm. For gel source data, see Supplementary Fig. 1.

Next, we tracked a cohort of asynchronously growing cells for 30 h and performed in silico alignment of the cell lineages according to cell cycle position based on the PCNA signal (Extended Data Fig. 3a). As expected, 53BP1 nuclear bodies marking regions of inherited endogenous replication stress appeared primarily after cell division in G1 and were cleared as cells entered S phase (Extended Data Fig. 3b,c). By contrast, when mild replication stress was induced by a low dose (200 nM) of the DNA polymerase inhibitor aphidicolin (APH), which did not induce measurable amounts of DNA damage as detected by western blot analysis of the DNA damage markers phosphorylated KAP1 (pKAP1) and pRPA (Extended Data Fig. 3d and Supplementary Fig. 1), 53BP1 foci formation was slightly enhanced and occurred not only in G1 cells but also during S-phase progression (Extended Data Fig. 3e,f). Inhibition of the central checkpoint kinase ATR resulted in more severely perturbed replication patterns and in elevated 53BP1-marked genomic lesions after cell division (Extended Data Fig. 3g,h). ATR inhibition was also associated with DNA damage as detected by pKAP1 and pRPA (Extended Data Fig. 3d). Consistent with the perturbed replication patterns observed in single cells after APH and ATR inhibitor (ATRi) treatment (Extended Data Fig. 3e,g), cell population based EdU profiles were also altered (Supplementary Fig. 2a). Both treatments resulted in increased PCNA foci in cells, which, according to EdU and DNA content, would be classified as G2, indicating replication-stress-induced DNA synthesis beyond normal S phase (Supplementary Fig. 2a–d).

**Extended Data Fig. 4:**
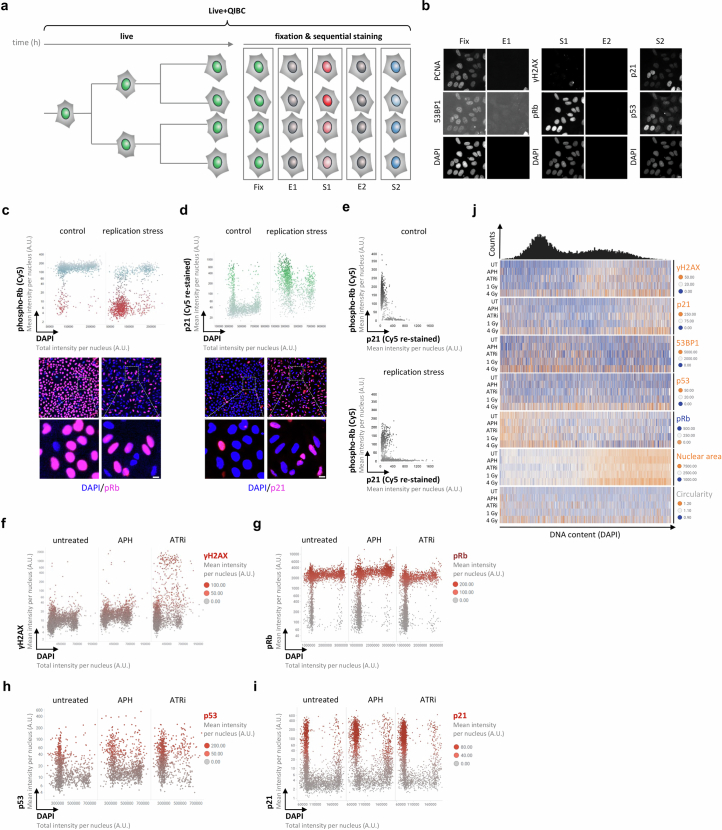
Sequential stainings post multigenerational live cell imaging. (**a**) Schematic representation of the Live+QIBC image analysis pipeline. E, antibody elution; S, staining. (**b**) Representative images of sequential staining and antibody elution steps in the Live+QIBC image analysis pipeline with 53BP1-PCNA U-2 OS cells. Scale bar, 10 µm. (**c, d**) Scatter plots and representative images of control cells or cells undergoing replication stress sequentially stained for phospho-Rb and p21 in the same colour channel. The total DAPI intensity per nucleus indicates DNA content, with 2 N (G1) on the left and 4 N (G2) on the right. Scale bars, 10 µm. (**e**) Direct comparison of the pRb signals and the p21 signals after antibody elution and re-staining. (**f**) Cell cycle resolved scatter plots of γΗ2ΑΧ mean intensities from untreated U-2 OS cells or U-2 OS cells treated with 0.2 µM aphidicolin or 1 µM ATR inhibitor for 48 h. (**g**) Cell cycle resolved scatter plots of pRb mean intensities from untreated U-2 OS cells or U-2 OS cells treated with 0.2 µM aphidicolin or 1 µM ATR inhibitor for 48 h. (**h**) Cell cycle resolved scatter plots of p53 mean intensities from untreated U-2 OS cells or U-2 OS cells treated with 0.2 µM aphidicolin or 1 µM ATR inhibitor for 48 h. (**i**) Cell cycle resolved scatter plots of p21 mean intensities from untreated U-2 OS cells or U-2 OS cells treated with 0.2 µM aphidicolin or 1 µM ATR inhibitor for 48 h. (**j**) Heatmaps of cell cycle resolved analysis of asynchronous U-2 OS cell populations treated as indicated for 24 h with 0.2 μM APH, 1 μM ATRi, or with IR (1 Gy and 4 Gy) followed by recovery for 24 h. Cells were sequentially stained for multiple DNA damage and cell cycle markers. Nuclear intensities (in the case of 53BP1 accumulated foci intensities) per cell and morphological features are indicated by colour-code. Note that the colour-code was deliberately reversed for pRb.

To extend these analyses to additional stress markers, including post-translational protein modifications inaccessible for live-cell imaging, we combined multigenerational time-lapse microscopy for lineage analyses with sequential immunofluorescence staining^35^ of the tracked cells in a cell-cycle-resolved manner, that is, live + quantitative image-based cytometry (Live+QIBC); Extended Data Fig. 4a). For multiplexing after live imaging, we focused on γH2AX, a DNA damage-induced histone phosphorylation, on pRb as a marker of cell cycle commitment after cell division, on the tumour suppressor protein p53, which is stabilized after DNA damage, and on its downstream target, the CDK inhibitor p21. All markers showed nuclear signals that could be eluted efficiently (Extended Data Fig. 4b). No cross-talk between sequential stainings was observed, as demonstrated by the inversely correlated signals of pRb and p21 when imaged in the same colour channel (Extended Data Fig. 4c–e). Overall, APH and ATRi treatments increased the levels of γH2AX, p53 and p21, and reduced pRb (Extended Data Fig. 4f–i), and treatment effects on single cells could be analysed in a cell-cycle-resolved manner on the basis of the DAPI signal (Extended Data Fig. 4j).

## Emergence of sister cell heterogeneity

**Fig. 2:**
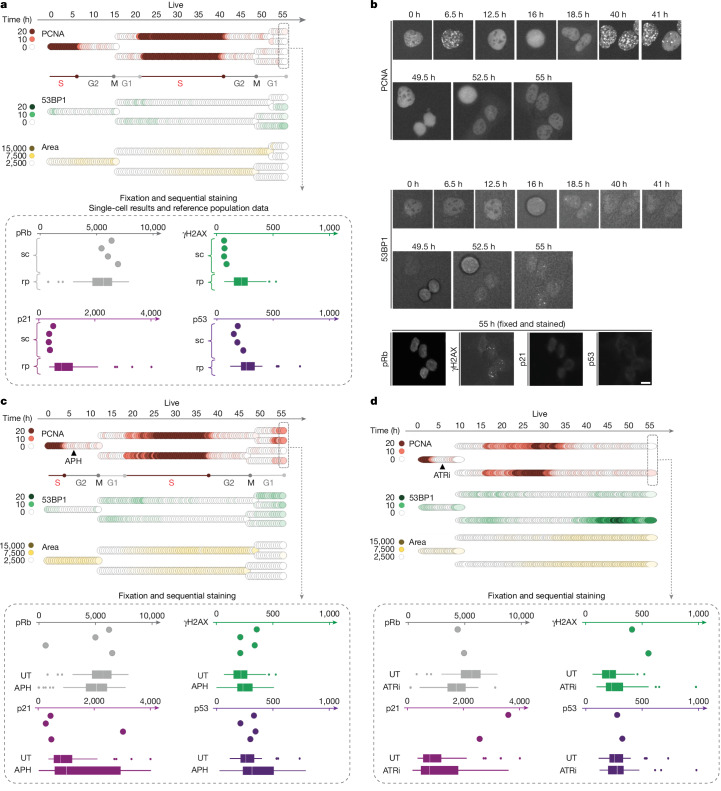
Enhanced sister cell heterogeneity after replication stress. **a**, A representative single-cell lineage from untreated 53BP1–PCNA U-2 OS cells. PCNA foci, 53BP1 foci and the area of the nucleus over 55 h of live imaging are shown. Sequential staining intensities from individual cells of the lineage (sc) and the mean intensities from the reference population (rp) are depicted for the markers pRb, γH2AX, p21 and p53. **b**, Representative video stills from the single-cell lineage in **a** and corresponding images from the sequential staining of cells for pRb, γH2AX, p21 and p53. Scale bar, 10 µm. **c**, Single-cell lineage from 53BP1–PCNA U-2 OS cells treated with 0.2 µM APH during the G2 phase of the cell cycle. PCNA foci, 53BP1 foci and area of the nucleus over 55 h of live imaging are shown. Sequential staining intensities from individual cells of the lineage and mean intensities from the corresponding reference populations are shown. UT, untreated. **d**, Single-cell lineage from 53BP1–PCNA U-2 OS cells treated with 1 µM of ATRi during the G2 phase of the cell cycle. PCNA foci, 53BP1 foci and the area of the nucleus over 55 h of live imaging are shown. Sequential staining intensities from individual cells of the lineage and mean intensities from the corresponding reference populations are shown. For the box plots, the box limits show the interquartile range (IQR, 25th percentile (Q1) to 75th percentile (Q3)), the median (centre line) and the whiskers define the lower and upper adjacent value; the dots show outliers smaller than Q1 − 1.5 × IQR and greater than Q3 + 1.5 × IQR; *n* > 35 end-point measurements per sample. Drugs in **c** and **d** were removed after 24 h.

We combined single-cell tracks from multigenerational live-cell imaging for 55 h, covering up to three cell generations, with end-point measurements obtained by sequential staining. As before (Extended Data Fig. 3), unchallenged asynchronously growing cells had comparatively sharp cell cycle phase transitions, daughter cells showed little heterogeneity in G1 duration and S-phase onset after cell division, and the nuclear area increased gradually during the cell cycle until mitosis (Fig. 2a,b). DNA damage levels measured by 53BP1 were low and mostly confined to G1, and the granddaughter cell generation had background levels of γH2AX, p53 and p21, and high pRb. By contrast, asynchronously growing cells treated with a low dose of APH when they were in G2 showed perturbed S-phase entry in the daughter cell generation, with seemingly normal S-phase exit into G2 and normal cell division (Fig. 2c). However, compared with untreated cells, the granddaughter cells had elevated γH2AX and p53 levels, and individual granddaughter cells showed high p21 and low pRb, which was associated with defective S-phase commitment (Fig. 2c). When asynchronously growing cells were treated with ATRi in G2, the next cell cycle seemed prolonged, with sister cells showing perturbed DNA replication and heterogeneity in S-phase duration, as well as 53BP1 foci formation, and γH2AX and p21 induction (Fig. 2d).

**Extended Data Fig. 5:**
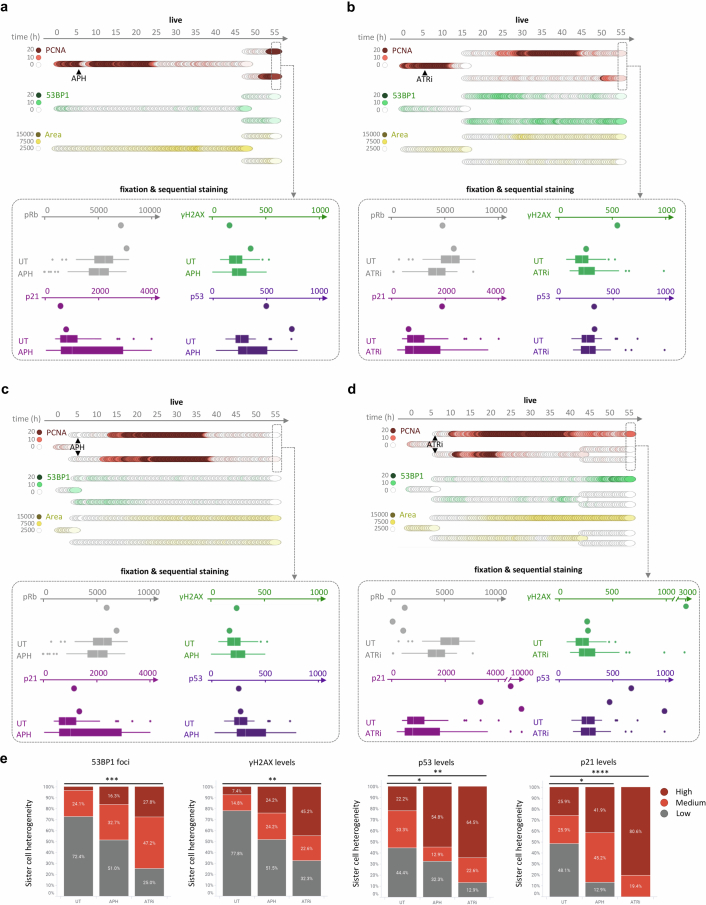
Single cell tracks from cells treated with replication stress inducing agents. (**a**) Single cell lineage from 53BP1-PCNA U-2 OS cells treated with 0.2 µM aphidicolin during the S phase of the cell cycle. PCNA foci, 53BP1 foci and area of the nucleus over 55 h of live imaging are depicted. Sequential staining intensities from individual cells of the lineage and mean intensities from corresponding reference populations are depicted for the markers pRB, γH2AX, p21 and p53. (**b**) Single cell lineage from 53BP1-PCNA U-2 OS cells treated with 1 µM ATR inhibitor during the S phase of the cell cycle. PCNA foci, 53BP1 foci and area of the nucleus over 55 h of live imaging are depicted. Sequential staining intensities from individual cells of the lineage and mean intensities from corresponding reference populations are depicted for the markers pRB, γH2AX, p21 and p53. (**c**) Single cell lineage from 53BP1-PCNA U-2 OS cells treated with 0.2 µM aphidicolin during the G1 phase of the cell cycle. PCNA foci, 53BP1 foci and area of the nucleus over 55 h of live imaging are depicted. Sequential staining intensities from individual cells of the lineage and mean intensities from corresponding reference populations are depicted for the markers pRB, γH2AX, p21 and p53. (**d**) Single cell lineage from 53BP1-PCNA U-2 OS cells treated with 1 µM ATR inhibitor during the G1 phase of the cell cycle. PCNA foci, 53BP1 foci and area of the nucleus over 55 h of live imaging are depicted. Sequential staining intensities from individual cells of the lineage and mean intensities from corresponding reference populations are depicted for the markers pRB, γH2AX, p21 and p53. Drugs in (a-d) were removed after 24 h. (**e**) Sister cell heterogeneity analysis of 53BP1-PCNA U-2 OS cells treated either with 0.2 µM aphidicolin or with 1 µM ATR inhibitor. Heterogeneity was scored for 53BP1 foci between sister cells of the F_1_ generation in G1 phase, whereas γΗ2ΑΧ, p53 and p21 levels were scored at the end of 55 h of live imaging. Statistical analysis by Chi-Square test. Box plot limits indicate 25th percentile (Q1) and 75th percentile (Q3); boxes represent interquartile range (IQR, Q3-Q1) with medians (solid lines). Whiskers define lower and upper adjacent value; dots show outliers smaller than Q1 - 1.5 × IQR and greater than Q3 + 1.5 × IQR. Source data

Cells that received APH during S-phase progression showed S-phase prolongation and signs of DNA synthesis in an extended G2 phase, yet their progeny entered S phase seemingly normally (Extended Data Fig. 5a). However, cells that received ATRi during S-phase showed G2 acceleration and increased sustained 53BP1 foci in the next cell generation, associated with heterogenous and strongly impaired S-phase entry (Extended Data Fig. 5b). Finally, cells that received APH before S-phase entry in G1 showed greatly prolonged cell cycle duration without further cell division during the 55 h of the experiment (Extended Data Fig. 5c), whereas sister cells that received ATRi in G1 exhibited asymmetric S-phase duration and DNA damage accumulation, which was associated with p21 induction and loss of pRb (Extended Data Fig. 5d). When scored across multiple sister cell pairs, sister cell heterogeneity was increased at the level of 53BP1 nuclear bodies, γH2AX levels, and p53 and p21 induction (Extended Data Fig. 5e). Other markers of impaired cell cycle progression and increased heterogeneity were also elevated after APH or ATRi treatment, including perturbed replication (assessed by PCNA patterns), periods with highly elevated nuclear 53BP1 foci and reduced numbers of cell division during the time-lapse imaging period (Supplementary Fig. 2e).

Considering the observed p53–p21 induction and their tumour-suppressive functions, we performed multigenerational cell tracking after p53 or p21 depletion. Both knockdowns seemed to accelerate cell cycle progression and to synergize with APH and ATRi to cause perturbed replication, increased DNA damage and enhanced cellular heterogeneity (Supplementary Fig. 3a–k and Supplementary Fig. 4a–i). Moreover, depletion of the tumour suppressor AMBRA1, which controls the G1/S transition through cyclin D regulation^36–38^, synergized with APH and especially ATRi (Supplementary Fig. 5a–d and Supplementary Fig. 6a,b).

Collectively, these results shed light onto how replication stress (here induced by APH or ATRi), defective checkpoint signalling (here induced by ATRi) or loss of tumour suppressor functions (here induced by p53, p21 or AMBRA1 depletion) induce cellular heterogeneity. Although analysed merely at the phenotypic level, it is conceivable that such changes may accelerate cellular transformation and evolution of drug resistance.

**Extended Data Fig. 6:**
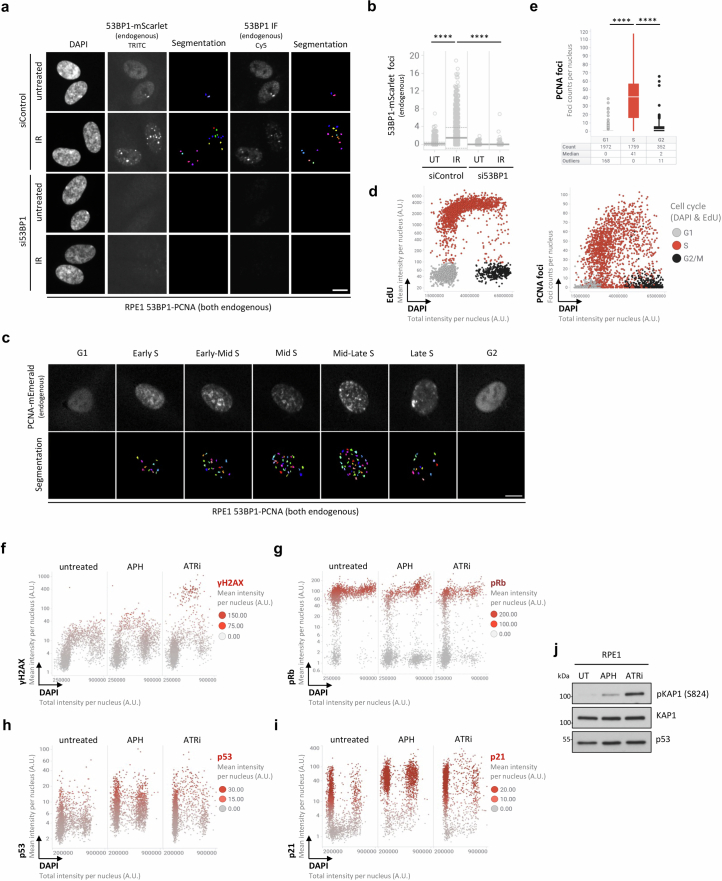
Validation of CRISPR-engineered 53BP1-PCNA RPE-1 cells. (**a**) 53BP1-PCNA RPE-1 cells were treated with siRNA for 72 h and IR (4 Gy, 2 h) as indicated and stained for 53BP1 (Cy5). The endogenous 53BP1-mScarlet and antibody-based signals are shown, together with their segmentation masks. Scale bar, 10 µm. (**b**) High-content microscopy-based quantification of 53BP1-mScarlet foci using the samples and foci segmentation mask from (a). Statistical analysis by one-way ANOVA followed by Tukey’s post hoc test. (**c**) Video stills of 53BP1-PCNA RPE-1 cells at different cell cycle phases from G1 through S-phase and to G2. PCNA-mEmerald patterns and their segmentation are shown. Scale bar, 10 µm. (**d**) DAPI- and EdU-based cell cycle gating of 53BP1-PCNA RPE-1 cells and PCNA foci analysis in different cell cycle phases (colour-coded based on DAPI and EdU). (**e**) PCNA foci quantification from (d) in G1 versus S-phase versus G2 (based on DAPI and EdU). Statistical analysis by one-way ANOVA followed by Tukey’s post hoc test. (**f**) Cell cycle resolved scatter plots of γΗ2ΑΧ mean intensities from untreated RPE-1 cells or RPE-1 cells treated with 0.2 µM aphidicolin or 1 µM ATR inhibitor for 48 h. (**g**) Cell cycle resolved scatter plots of pRb mean intensities from untreated RPE-1 cells or RPE-1 cells treated with 0.2 µM aphidicolin or 1 µM ATR inhibitor for 48 h. (**h**) Cell cycle resolved scatter plots of p53 mean intensities from untreated RPE-1 cells or RPE-1 cells treated with 0.2 µM aphidicolin or 1 µM ATR inhibitor for 48 h. (**i**) Cell cycle resolved scatter plots of p21 mean intensities from untreated RPE-1 cells or RPE-1 cells treated with 0.2 µM aphidicolin or 1 µM ATR inhibitor for 48 h. (**j**) Western blot analysis of phospho-KAP1 in untreated RPE-1 cells or RPE-1 cells treated with 0.2 μM APH or 1 μM ATRi for 24 h. For gel source data, see Supplementary Fig. 1. Source data

To validate these findings in an independent cell system, we used CRISPR–Cas9-mediated genome engineering to fluorescently label endogenous 53BP1 and PCNA in hTERT-immortalized, non-cancer RPE-1 cells. Endogenous 53BP1–mScarlet foci were easily detectable, induced after ionizing radiation (IR), could be segmented, co-localized with antibody-stained 53BP1 and were specific as revealed by *53BP1* knockdown (Extended Data Fig. 6a,b). Endogenous PCNA–mEmerald patterns in RPE-1 cells were also detectable, could be segmented and marked EdU-positive S-phase cells (Extended Data Fig. 6c–e). Similar to U-2 OS cells, APH and ATRi increased γH2AX, p53 and p21 levels, and reduced pRb (Extended Data Fig. 6f–i), with APH hampering more the G2/M transition, as judged by the increase in 4N cells with low pRb and high p21, and ATRi causing cells to accumulate in G1 with low pRb and high p21 (Extended Data Fig. 6f–i). This is consistent with APH-mediated slowing of DNA replication and ATRi-mediated inhibition of the G2/M checkpoint. Similar to U-2 OS cells, APH caused mild and ATRi more severe DNA damage, as measured by KAP1 phosphorylation (Extended Data Fig. 6j). Tracking of endogenously tagged RPE-1 cells across multiple cell generations was possible yet more difficult owing to their high motility (Supplementary Fig. 7a). Moreover, iterative staining was complicated by their loose attachment, resulting in cell loss during sequential rounds of washing and signal elution. Nevertheless, single-cell lineages confirmed the main phenotypes observed in U-2 OS cells, including asymmetric S-phase onset and perturbed replication patterns in individual sister cells after APH, ATRi and IR (Supplementary Fig. 7b–e).

## Sources of elevated heterogeneity

**Fig. 3:**
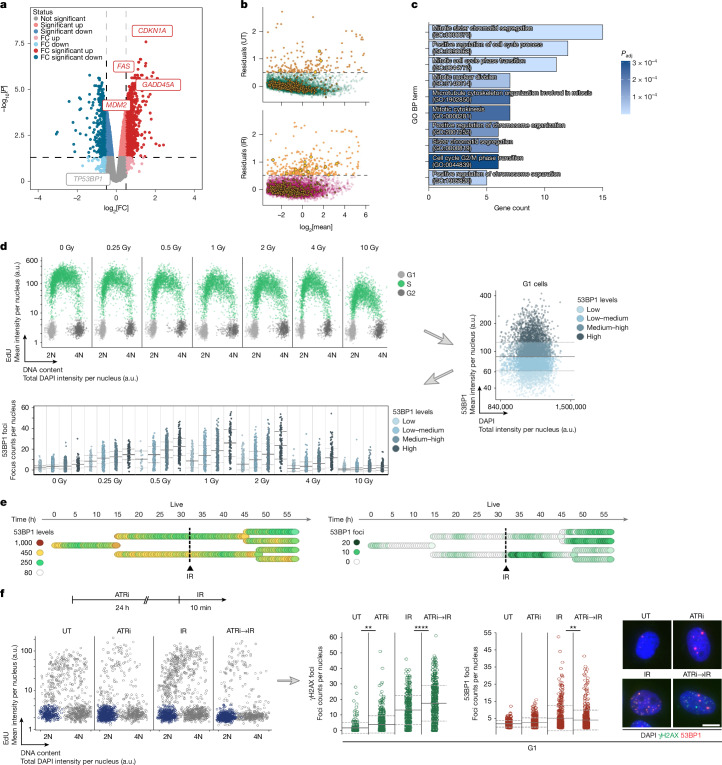
DNA-damage-induced transcriptional and phenotypic heterogeneity. **a**, Volcano plot showing differentially expressed genes in U-2 OS cells 24 h after IR, derived from bulk RNA-seq analysis. **b**, Residual variability of gene expression after a linear fit of the log_2_-transformed s.d. to the log_2_-transformed mean expression in U-2 OS cells 48 h after IR from scRNA-seq analysis. Positive residual values indicate a higher s.d. than expected. Genes of the DNA damage response pathway (GO: 0006974) are highlighted by closed circles. *P* = 2.45 × 10^−12^; odds ratio = 1.90 (Fisher’s exact test on genes with residuals > 0.5 versus genes with residuals ≤ 0.5). **c**, GO analysis of genes with specifically increased residuals (>0.5) after IR treatment. BP, biological process. **d**, U-2 OS cells were treated with the indicated doses of IR and fixed 45 min later for 53BP1 foci analysis by QIBC. The G1 populations were selected based on DAPI and EdU and categorized on the basis of the nuclear 53BP1 levels. The IR-induced 53BP1 foci formation in G1 cells was then analysed as a function of IR dose and 53BP1 expression. The horizontal solid lines represent the mean and the horizontal dashed lines represent s.d. **e**, Single-cell lineage from 53BP1–PCNA U-2 OS cells treated with 4 Gy IR as indicated. Nuclear 53BP1 levels (a.u.) (left) and 53BP1 foci formation (right) are depicted by the colour code. **f**, U-2 OS cells were treated as indicated (1 µM ATRi; 4 Gy IR) and γH2AX and 53BP1 foci in G1 cells were analysed by QIBC. Example images are shown to the right. Scale bar, 10 µm. Statistical analysis by one-way ANOVA followed by Tukey’s post hoc test. **P ≤ 0.01, ****P ≤ 0.0001. Source data

**Extended Data Fig. 7:**
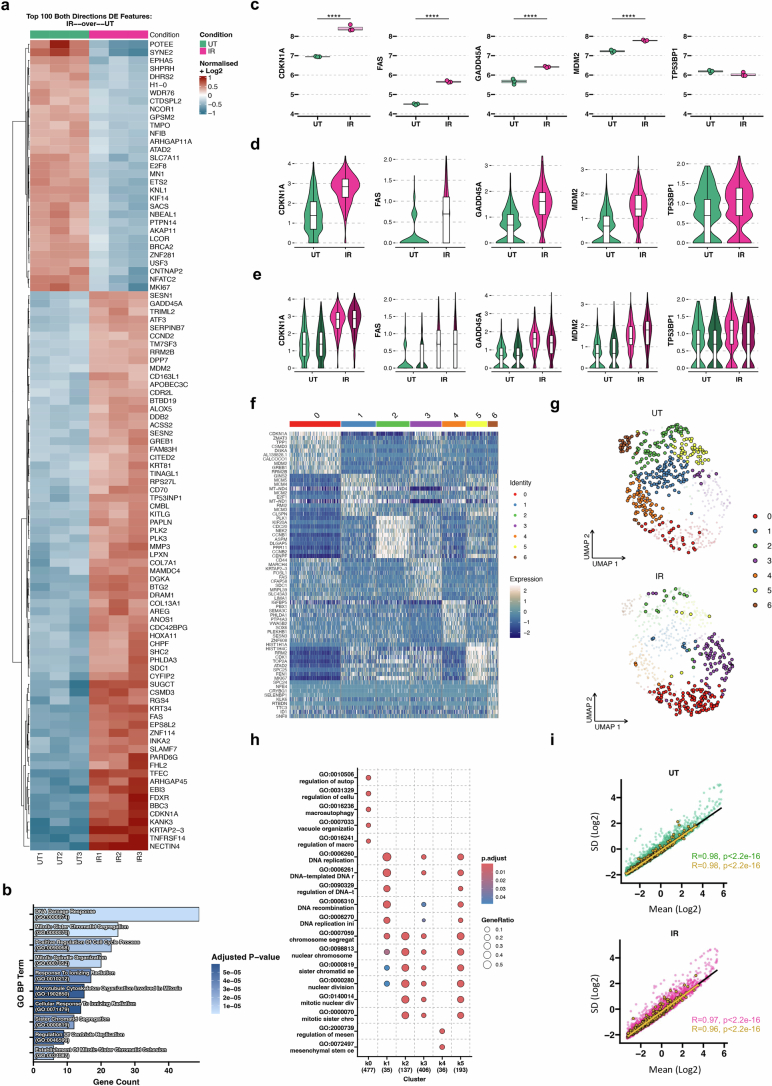
DNA damage-induced transcriptional heterogeneity. (**a**) Heatmap of the top 100 differentially expressed (DE) genes ranked by adjusted p-value upon IR treatment (4 Gy, 24 h) from bulk RNA-seq. (**b**) Gene ontology (GO) analysis of up-regulated DE genes upon IR treatment from bulk RNA-seq. (**c**) Example boxplots of DE genes after IR-induced DNA damage from bulk RNA-seq, including the CDK inhibitor and cell cycle regulator CDKN1A (p21), the pro-apoptotic cell death receptor FAS, the growth arrest and DNA damage-inducible protein GADD45A, and the p53 target gene and p53 regulator MDM2. TP53BP1 (53BP1) is shown as a control gene, the expression of which was not upregulated after IR. (**d**) Violin plots showing gene expression changes from scRNA-seq of the same genes depicted in (c). (**e**) Validation of the observed gene expression changes in a second set of cell samples subjected independently to scRNA-seq. (**f**) Heatmap of the top 10 marker genes identified from unsupervised clustering analysis of the scRNA-seq data from untreated and IR treated cells. (**g**) UMAPs (uniform manifold approximation and projection) of the scRNA-seq data colour-coded by the clusters identified in (f) and split by condition (UT, IR). (**h**) GO pathway enrichment in the single cell clusters identified in (f), excluding the smallest cluster k6, which did not yield any significantly enriched GO term. (**i**) Standard deviation (SD) versus Mean analysis of the scRNA-seq data. DNA damage response genes (GO: 0006974) highlighted in orange.

To investigate potential sources of heterogeneity, we first performed bulk RNA-sequencing (RNA-seq) analysis, revealing differentially expressed genes after IR-induced DNA damage (Extended Data Fig. 7a). We found that DNA damage response and cell cycle regulating genes were enriched (Extended Data Fig. 7b), with *CDKN1A* (encoding p21), *FAS*, *GADD45A* and *MDM2* being among the most highly induced genes (Fig. 3a and Extended Data Fig. 7c). By contrast, *TP53BP1* expression was not significantly induced (Fig. 3a and Extended Data Fig. 7c), consistent with its constitutive expression and mechanism of action through recruitment to sites of DNA damage. Single-cell RNA-seq (scRNA-seq) analysis showed very similar (Extended Data Fig. 7d) and reproducible effects (Extended Data Fig. 7e). A clustering analysis of the scRNA-seq results revealed primarily genes involved in DNA replication, repair and cell division as main drivers of DNA-damage-induced cellular heterogeneity (Extended Data Fig. 7f–h). We next analysed highly variable genes in the unchallenged and IR-treated conditions by computing the mean and s.d. of the normalized expression values and performing a linear fit of the log_2_-transformed s.d. versus the log_2_-transformed mean (Extended Data Fig. 7i). Highly variable genes were identified as those of which the residual of the fit was above 0.5. Significantly more highly variable genes were found in the IR-treated condition compared with in the untreated condition, including some genes of the DNA damage response pathway, which are highlighted by closed circles (Fig. 3b). Further analysis showed that genes with high variability (residuals above 0.5) only in the IR condition were predominantly involved in cell cycle regulation (Fig. 3c), suggesting that DNA damage-induced increased cellular heterogeneity is linked to cell-to-cell differences in cell cycle control.

To investigate potential sources of non-genetic heterogeneity beyond induced gene expression changes, we turned to 53BP1, the expression of which was not significantly changed after DNA damage (Extended Data Fig. 7c). 53BP1 requires self-association through its oligomerization domain for foci formation at sites of DNA damage and shows features of phase separation, which is greatly affected by protein concentration^33,39^. Consistently, we found that nuclear 53BP1 concentration, at the single-cell level (analysed specifically in G1 cells to avoid interference from DNA replication), limits 53BP1 foci formation in response to increasing doses of IR (Fig. 3d). Consistently, we identified single-cell lineages, in which nuclear 53BP1 levels correlated with the strength of 53BP1 foci formation after IR in sister cells (Fig. 3e). Taken together, these results suggest that 53BP1 recruitment is limited by its nuclear concentration, and that heterogeneity in 53BP1 between individual cells impacts the response to DNA damage. Consistent with 53BP1 being limiting for the response to DNA damage at single-cell level, when cells were pretreated with ATRi to trigger replication stress-induced 53BP1 condensates in G1 cells, before acute DNA damage induction by IR, the formation of ATRi-induced 53BP1 condensates impaired the 53BP1 response to acute DNA damage (Fig. 3f). Different from γH2AX foci, which showed an additive increase after ATRi and IR, 53BP1 recruitment to IR-induced breaks was reduced due to its sequestration at replication stress-induced DNA lesions, which in turn resulted in IR-induced γH2AX foci not being served by 53BP1 (Fig. 3f).

## Heritable changes after acute DNA damage

**Extended Data Fig. 8:**
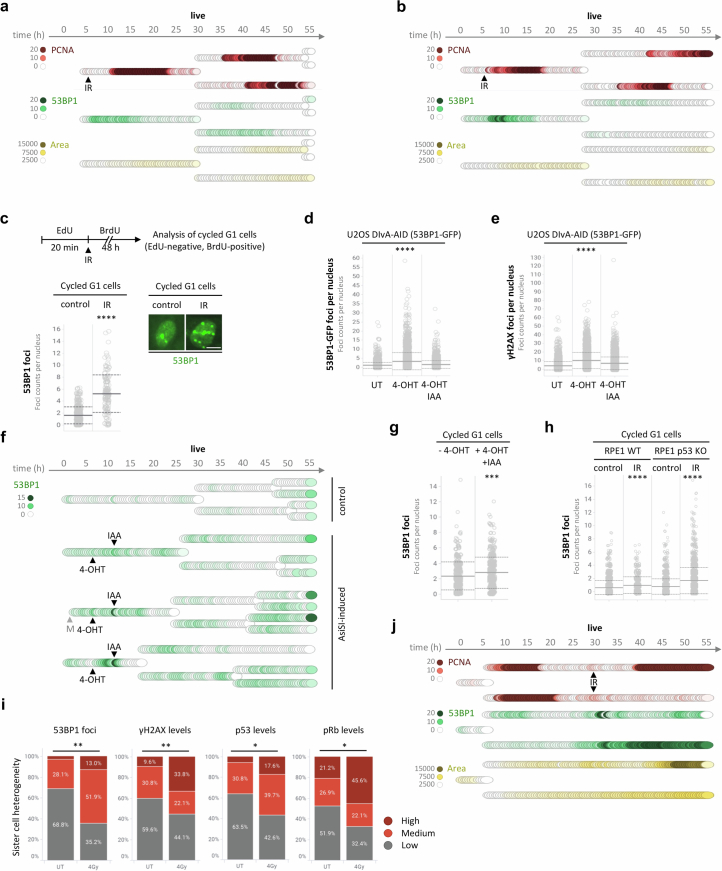
DNA damage in G1 generates heritable lesions associated with sister cell asymmetry in the next cell generation. (**a, b**) Single cell lineages from 53BP1-PCNA U-2 OS cells treated with 4 Gy IR during the G1 phase of the cell cycle. (**c**) QIBC-derived scatter plot of IR-induced 53BP1 foci in the next G1 phase. U-2 OS cells were pulsed with 10 µM EdU for 20 min before irradiation (4 Gy) and then allowed to cycle in the presence of 10 µM BrdU for 48 h to overcome the G2/M checkpoint. DNA was denatured for BrdU detection and cycled G1 cells negative for EdU and positive for BrdU were analysed. Horizontal solid lines represent the mean and horizontal dashed lines represent standard deviation. Example images are shown on the right. Scale bar, 10 µm. Statistical analysis by two-tailed unpaired t-test. (**d**) Endonuclease-induced DSBs in 53BP1-GFP DIvA-AID U-2 OS cells. AsiSI was induced by 4-OHT (300 nM) for 4 h, followed by fixation or release into medium without 4-OHT and containing auxin (IAA) (500 µM) for 20 h. 53BP1-GFP foci were analysed by QIBC. Statistical analysis by one-way ANOVA followed by Tukey’s post hoc test. (**e**) In the same cells as in (d), γH2AX foci were analysed by QIBC. Statistical analysis by one-way ANOVA followed by Tukey’s post hoc test. (**f**) Single cell lineages from 53BP1-GFP DIvA-AID U-2 OS cells treated with 4-OHT (300 nM) and IAA (500 µM) as indicated. 53BP1-GFP foci formation is depicted by the colour code. M, mitosis. (**g**) As in (c), 53BP1 foci in cycled G1 53BP1-GFP DIvA-AID U-2 OS cells after transient AsiSI induction (4-OHT for 4 h, followed by release into medium with auxin (IAA) and without 4-OHT for 44 h). Statistical analysis by two-tailed unpaired t-test. (**h**) As in (c), 53BP1 foci in cycled G1 RPE-1 cells (p53 WT and p53 KO) after IR (4 Gy). Statistical analysis by one-way ANOVA followed by Tukey’s post hoc test. (**i**) Sister cell heterogeneity in 53BP1-PCNA U-2 OS cells treated with IR (4 Gy) in G1. Heterogeneity was scored for 53BP1 foci between sister cells of the F_1_ generation in G1 phase, whereas p53, γH2AX and pRB levels were scored at the end of 55 h of live imaging. Statistical analysis by Chi-square test. (**j**) Single cell lineage of daughter cells irradiated with IR (4 Gy). Two rounds of replication associated with an increase in nuclear area can be seen for one of the two daughter cells. Source data

To investigate further how a singular event of acute DNA damage affects cellular heterogeneity, we focused on IR. As IR, in principle, induces DNA strand breaks at random sites in replicating genomes, which can lead to non-symmetric damage loads in duplicated DNA, we deliberately focused on cells that received IR in the G1 phase of the cell cycle. DNA breakage in G1 and their subsequent repair, indicated by the induction and disappearance of 53BP1 foci, was associated with pronounced heterogeneity in the next cell generation between sister cells (Extended Data Fig. 8a,b). Similar effects could be observed in RPE-1 cells (Supplementary Fig. 7e).

To test whether we could recapitulate these findings in a larger cohort of cells, we sequentially pulse-labelled asynchronously growing cell populations with EdU and BrdU to identify cells that, at the time of irradiation, were in G1 and that had then, after transient cell cycle arrest, gone through a complete cell cycle until being analysed in their next G1 phase (Supplementary Fig. 8a). Notably, the irradiated cycled cells had more 53BP1 nuclear bodies in G1 compared with their controls (Extended Data Fig. 8c), consistent with the live-cell tracking data. As IR causes not only DNA double-stranded breaks (DSBs), but can, through direct and indirect routes, induce a variety of DNA lesions, we used DSB inducible via AsiSI (DIvA) cells for endonuclease-mediated DSB formation^40^. DSBs were induced by 4-hydroxytamoxifen (4-OHT) as measured by 53BP1 and γH2AX foci formation, and most of these foci disappeared after indole-3-acetic acid (IAA)-mediated degradation of the AsiSI endonuclease fused to an auxin-inducible degron, indicating repair (Extended Data Fig. 8d,e). 4-OHT-induced repairable DSBs were also detected by live imaging of DIvA cells, and they were associated with DNA lesions and sister cell heterogeneity in daughter and granddaughter cells (Extended Data Fig. 8f), similar to IR-induced DNA breaks. Likewise, cycled DIvA cells after transient AsiSI induction had more 53BP1 nuclear bodies in G1 compared with their uninduced controls (Extended Data Fig. 8g). Consistent effects were observed in RPE-1 cells, especially after p53 loss (Extended Data Fig. 8h).

When we quantified cellular heterogeneity across multiple pairs of tracked sister cells of which the parental cells had received IR in G1, sister cell asymmetry was increased at several levels (53BP1, γH2AX, p53, p21) compared with the untreated control cells (Extended Data Fig. 8i and Supplementary Fig. 8b). Thus, even a single, transient genotoxic event in the parental DNA may result in a persistent genomic scar that affects cell cycle commitment and replication timing in the next cell generation, leading to increased asymmetry between sister cells and increased phenotypic heterogeneity within a cell population. Alternatively, damaged DNA might sequester proteins with dual functions in DSB repair and replication fork protection away from replication factories, thereby affecting replication fidelity indirectly, or aberrant repair processes such as breakage–fusion–bridge events might induce sister cell heterogeneity. These mechanisms are not mutually exclusive, and they jointly imply that a single genotoxic event can induce heterogeneity and lasting phenotypic changes in a cell population.

## Genotoxic-stress-induced polyploidization

**Fig. 4:**
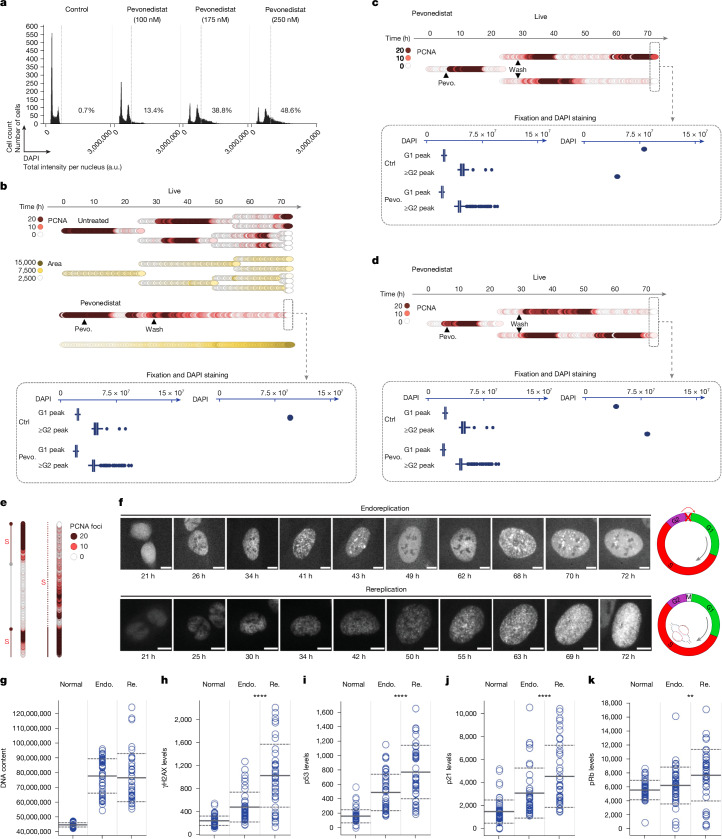
Live+QIBC reveals two distinct routes towards polyploidization. **a**, High-content-microscopy-derived cell cycle profiles of U-2 OS cells treated with increasing doses of pevonedistat (pevo.; MLN4924) for 24 h and released into fresh medium for 42 h. The percentage of hyperploid cells (>4N DNA content, marked by the dashed vertical line) is indicated. **b**, A single-cell lineage from 53BP1–PCNA U-2 OS control cells and cells treated with 175 nM of pevonedistat. The total DAPI intensity of the nucleus from the corresponding reference populations (ctrl) is depicted as well as from the pevonedistat-treated single cell at the end of live imaging. **c**, A single-cell lineage from 53BP1–PCNA U-2 OS cells treated with 175 nM of pevonedistat. The total DAPI intensity of the nucleus from the corresponding reference populations is depicted as well as from the pevonedistat-treated single cells at the end of live imaging. **d**, A second example of a single cell lineage from 53BP1–PCNA U-2 OS cells (details as in **c**). **e**, Representative single-cell analyses of the PCNA patterns of cells that undergo endoreplication or rereplication, respectively. **f**, Representative video stills of 53BP1–PCNA U-2 OS cells undergoing either endoreplication or rereplication after being treated with pevonedistat. PCNA patterns are depicted. Schematics depicting differences between endoreplication and rereplication are shown on the right. Scale bars, 10 µm. **g**, The total DAPI intensity (a.u.) of normal G2 cells and cells that underwent either endoreplication (endo.) or rereplication (re.) after being treated with pevonedistat. **h**, The mean nuclear intensity of γH2AX of normal G2 cells and cells that underwent either endoreplication or rereplication after being treated with pevonedistat. **i**, As in **h** for p53 intensity. **j**, As in **h**, but for p21 intensity. **k**, As in **h**, but for pRb intensity. Statistical analysis was performed using two-tailed unpaired *t*-tests between endoreplicating and rereplicating cells. The horizontal solid lines represent the mean and horizontal dashed lines represent s.d. The box plots in **b**–**d** show the IQR (box limits), with median (centre line) and the whiskers define the lower and upper adjacent value; dots show outliers greater than Q3 + 1.5 × IQR. Source data

**Extended Data Fig. 9:**
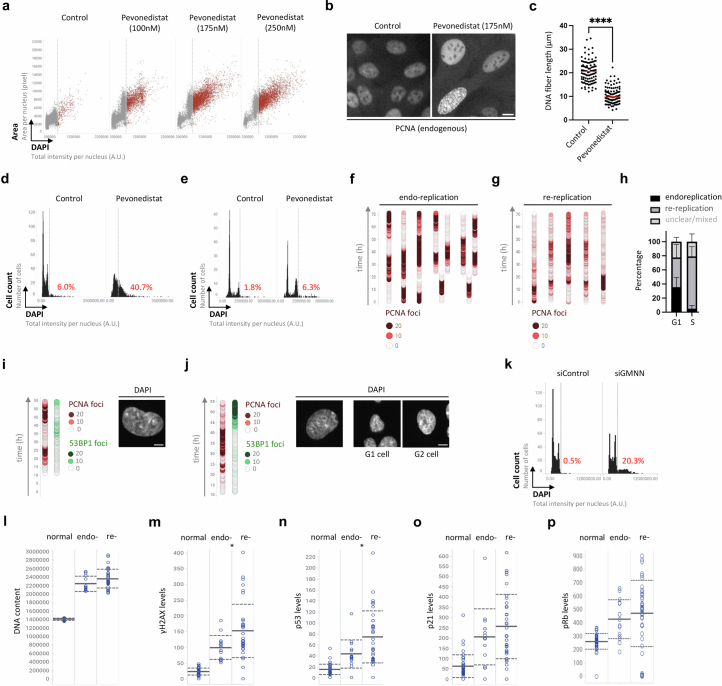
Polyploidization after treatment with Pevonedistat or depletion of Geminin. (**a**) Cell cycle resolved scatter plots of DNA content and area of the nucleus of cells shown in Fig. 4a. Hyperploid cells (> 4 N DNA content, marked by the dashed vertical line) are coloured in red. (**b**) Representative images of 53BP1-PCNA U-2 OS cells either untreated or treated with 175 nM of Pevonedistat for 24 h and allowed to recover for 42 h. Scale bar, 10 µm. (**c**) DNA fibre analysis of U-2 OS cells either untreated or treated with 175 nM of Pevonedistat for 24 h and then allowed to recover for 42 h. Statistical analysis by two-tailed unpaired t-test. (**d**) High-content microscopy derived cell cycle profiles of HCT 116 cells treated with 250 nM of Pevonedistat for 24 h. The percentage of hyperploid cells (> 4 N DNA content, marked by the dashed vertical line) is indicated. (**e**) High-content microscopy derived cell cycle profiles of RPE-1 cells treated with 175 nM of Pevonedistat for 24 h and then released into fresh medium for 42 h. The percentage of hyperploid cells (> 4 N DNA content, marked by the dashed vertical line) is indicated. (**f**) Representative single cell lineages depicting endoreplication after Pevonedistat exposure. (**g**) Representative single cell lineages depicting re-replication after Pevonedistat exposure. (**h**) Bar chart depicting the frequency of endo- and re-replication in 53BP1-PCNA U-2 OS cells treated either in G1 or S phase with 175 nM of Pevonedistat for 24 h and then released for 42 h. Mean ± SD are depicted. (**i**) Single cell lineage depicting endoreplication from live imaging of 53BP1-PCNA RPE-1 cells that were treated with Pevonedistat for 48 h. PCNA patterns and 53BP1 foci are shown. Scale bar, 10 µm. (**j**) Single cell lineage depicting re-replication from live imaging of 53BP1-PCNA RPE-1 cells that were treated with Pevonedistat for 48 h. PCNA patterns and 53BP1 foci are shown. Scale bar, 10 µm. (**k**) High-content microscopy derived cell cycle profiles of U-2 OS cells depleted of Geminin for 72 h. The percentage of hyperploid cells (> 4 N DNA content, marked by the dashed vertical line) is indicated. (**l**) Scatter plot of total DAPI intensity of normal G2 cells and Geminin depleted cells that underwent either endo- or re-replication. (**m**) Scatter plot of mean nuclear intensity of γH2AX of normal G2 cells and Geminin depleted cells that underwent either endo- or re-replication. (**n**) As in (m) for p53. (**o**) As in (m) for p21. (**p**) As in (m) for pRb. Statistical analysis was performed with two-tailed unpaired t-test between endo- and re-replication. Horizontal solid lines represent the mean and horizontal dashed lines represent standard deviation. Source data

One way to rapidly increase phenotypic heterogeneity of cancer cells is through polyploidization^17,18^. Polyploidization can lead to chromosomal instability and aneuploidy, which are frequently observed in cancers^19–21^ and which correlate with therapy resistance and poor patient outcomes^22,23^. Using time-resolved single-cell tracking, based on the PCNA signal, we observed examples of notably asymmetric replication patterns in sister cells, such as two rounds of replication without cell division in one of the two sister cells, which in principle could cause polyploidization in this branch (Extended Data Fig. 8j). To assess potential routes towards polyploidization in more detail, we used the NEDD8-activating enzyme (NAE) inhibitor pevonedistat (MLN4924, TAK924), a first-in-class anticancer drug that induces DNA rereplication, that is, the replication of already replicated DNA resulting in replication bubbles within larger replicons, through CDT1 stabilization^41–43^. Consistently, pevonedistat caused polyploidization in multiple cancer and non-cancer cell lines, which was associated with replication stress and an increase in nuclear size (Fig. 4a and Extended Data Fig. 9a–e). Moreover, single-cell tracking combined with sequential staining and DAPI-based DNA content measurements confirmed continuous replication patterns and polyploidization, in agreement with rereplication (Fig. 4b). However, tracking of sister cells also revealed distinct, asymmetric replication patterns consistent with endoreplication (also referred to as endoreduplication or endocycling, meaning multiple rounds of DNA replication without cell division) (Fig. 4c,d). Thus, two mechanistically distinct routes may lead to pevonedistat-induced polyploidization, rereplication and endoreplication (Fig. 4e,f and Extended Data Fig. 9f–j).

To assess whether the route towards increased ploidy matters for genome stability, we compared the DNA damage markers obtained through sequential staining after cell tracking in polyploid cells that had gone through either rereplication or endoreplication. Despite analysing cells with comparable DNA content after rereplication versus endoreplication, rereplicating cells acquired consistently higher DNA damage markers than cells that had gone through endoreplication (Fig. 4g–k). Similar results were obtained when polyploidization was induced by depletion of the replication inhibitor and CDT1 antagonist geminin^44,45^ (Extended Data Fig. 9k–p).

**Fig. 5:**
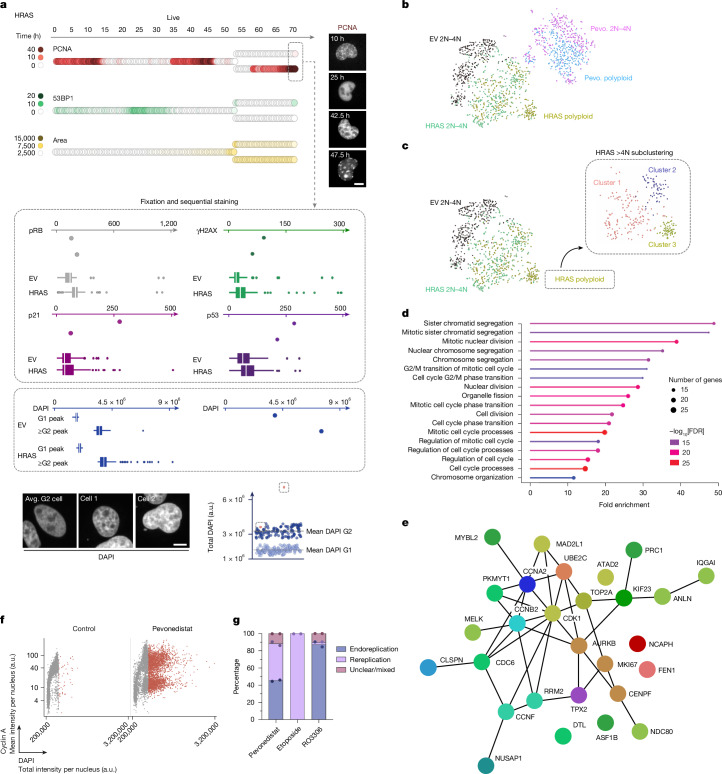
Oncogene overexpression induces two distinct routes towards polyploidization. **a**, Single-cell lineage from 53BP1–PCNA U-2 OS cells overexpressing HRAS. PCNA foci, 53BP1 foci and area of the nucleus over 70 h of live imaging are depicted. Sequential staining intensities from individual cells of the lineage and mean intensities from the corresponding reference populations are depicted for the markers pRb, γH2AX, p21 and p53. The total DAPI intensity per nucleus from the corresponding reference populations is depicted as well as from the single cells at the end of live imaging. Representative images of the polyploid cells are included as well as their position within the DAPI scatter plot of the G1 and G2 populations. Scale bars, 10 µm. The box plots show the IQR (box limits), with median (centre line) and the whiskers define the lower and upper adjacent value; dots show outliers greater than Q3 + 1.5 × IQR. Avg., average. **b**, *t*-Distributed stochastic neighbour embedding analysis of scRNA-seq results. 2N–4N, cells with 2N–4N DNA content; polyploid, cells with a DNA content >4N; pevo., pevonedistat-treated; HRAS, HRAS-overexpressing cells. *n* > 300 cells per condition. **c**, Subclustering of the HRAS polyploid sample with a Louvain resolution of 0.5. **d**, GO analysis of the top 28 genes from the overlap between HRAS polyploid and pevonedistat polyploid subclusters. FDR < 0.00001, *P* < 0.00001, FC > 4. **e**, STRING functional protein association network analysis of the top 28 genes. **f**, Cell-cycle-resolved nuclear cyclin A levels in U-2 OS cells either untreated or treated with 175 nM of pevonedistat for 24 h and then released into fresh medium for 42 h. **g**, The cellular behaviour towards polyploidy from live imaging experiments of 53BP1–PCNA U-2 OS cells treated with 175 nM of pevonedistat, 20 μM of etoposide or 5 μM of RO3306 for 24 h and then released for 42 h. Mean and individual values are depicted from two biological replicates based on live imaging data. Source data

**Extended Data Fig. 10:**
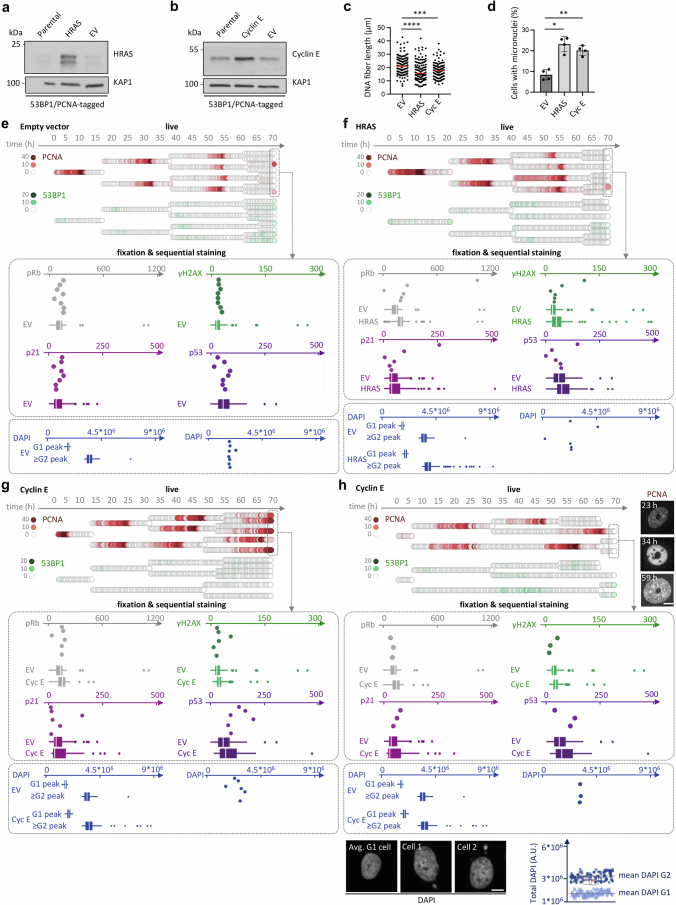
Oncogene-induced polyploidization. (**a**) Western blot analysis of 53BP1-PCNA U-2 OS cells overexpressing HRAS. (**b**) Western blot analysis of 53BP1-PCNA U-2 OS cells overexpressing Cyclin E1. (**c**) DNA fibre analysis of 53BP1-PCNA U-2 OS cells overexpressing either HRAS or Cyclin E1. Statistical analysis by one-way ANOVA followed by Tukey’s post hoc test. (**d**) Analysis of micronuclei induction in 53BP1-PCNA U-2 OS cells overexpressing either HRAS or Cyclin E1 compared to empty vector transfected control cells. Mean ± SD are depicted. Statistical analysis was performed with one-way ANOVA followed by Tukey’s post hoc test. (**e**) Single cell lineage from empty vector control 53BP1-PCNA U-2 OS cells. PCNA foci, 53BP1 foci and area of the nucleus over 70 h of live imaging are depicted. Sequential staining intensities from individual cells of the lineage and mean intensities from the corresponding reference population are depicted for the markers pRB, γH2AX, p21 and p53. The total DAPI intensity of the nucleus from the corresponding reference population is depicted as well as from the single cells at the end of live imaging. (**f**) Single cell lineage from 53BP1-PCNA U-2 OS cells overexpressing HRAS. Live+QIBC data is shown as in (e). (**g**) Single cell lineage from 53BP1-PCNA U-2 OS cells overexpressing Cyclin E1. Live+QIBC data is shown as in (e). (**h**) Single cell lineage from 53BP1-PCNA cells overexpressing Cyclin E1. Live+QIBC data is shown as in (e). Representative images of the polyploid cells are included as well as their position within the DAPI scatter plot of the G1 and G2 populations. Scale bars, 10 µm. Box plot limits indicate 25th percentile (Q1) and 75th percentile (Q3); boxes represent interquartile range (IQR, Q3-Q1) with medians (solid lines). Whiskers define lower and upper adjacent value; dots show outliers greater than Q3 + 1.5xIQR. For gel source data, see Supplementary Fig. 1. Source data

**Extended Data Fig. 11:**
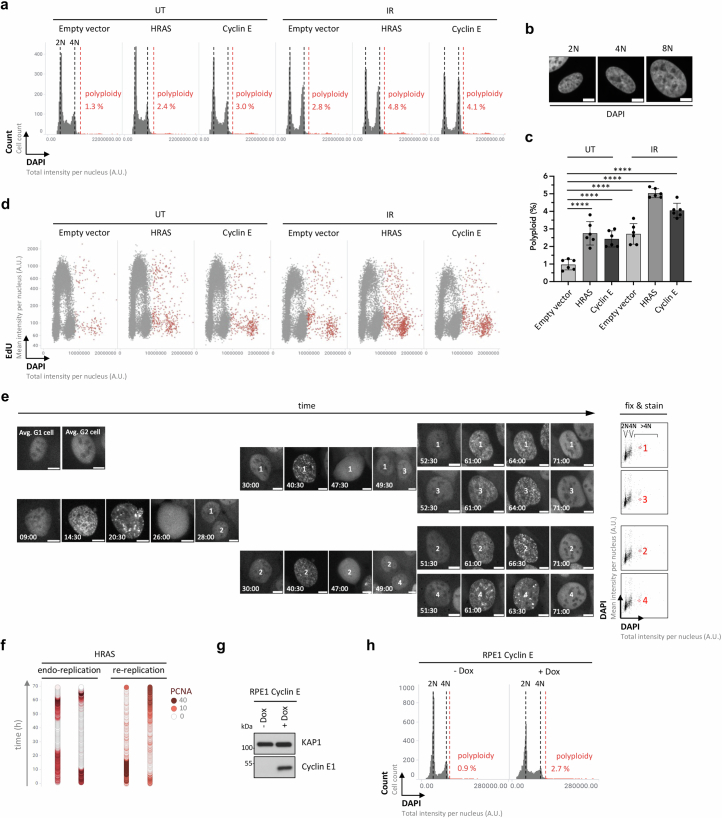
Polyploidization upon oncogene overexpression. (**a**) High-content microscopy derived cell cycle profiles of 53BP1-PCNA U-2 OS cells overexpressing either HRAS or Cyclin E1 that were treated or not with 4 Gy of IR. The percentage of hyperploid cells (> 4 N DNA content, marked by the dashed vertical line) is indicated. (**b**) Representative images of 53BP1-PCNA U-2 OS cells overexpressing HRAS with increased ploidy (2 N, 4 N, 8 N). Scale bar, 10 µm. (**c**) Bar chart depicting the percentage of polyploid cells overexpressing either HRAS or Cyclin E1 that were treated or not with 4 Gy of IR. Statistical analysis was performed with one-way ANOVA followed by Tukey’s post hoc test. (**d**) Cell cycle resolved EdU profiles of 53BP1-PCNA U-2 OS cells overexpressing HRAS or Cyclin E1. Cells were either untreated or treated with 4 Gy of IR. (**e**) Video stills of 53BP1-PCNA U-2 OS cells overexpressing HRAS that are maintaining polyploidy through multiple cell generations. The polyploid granddaughter cells with 8 N DNA content are identified in a scatter plot of mean vs total DAPI intensity post live imaging. (**f**) Representative single cell lineages from live imaging of 53BP1-PCNA U-2 OS cells that overexpress HRAS. Lineages depict endo-replication and re-replication, respectively. (**g**) Western blot validation of inducible expression of Cyclin E1 in 53BP1-PCNA RPE-1 cells. Cells were induced with Doxycycline (100 ng/ml) for 24 h. (**h**) High-content microscopy derived cell cycle profiles of 53BP1-PCNA RPE-1 with inducible expression of Cyclin E1. The percentage of hyperploid cells (> 4 N DNA content, marked by the dashed red vertical line) is indicated. Scale bars, 10 µm. For gel source data, see Supplementary Fig. 1. Source data

**Extended Data Fig. 12:**
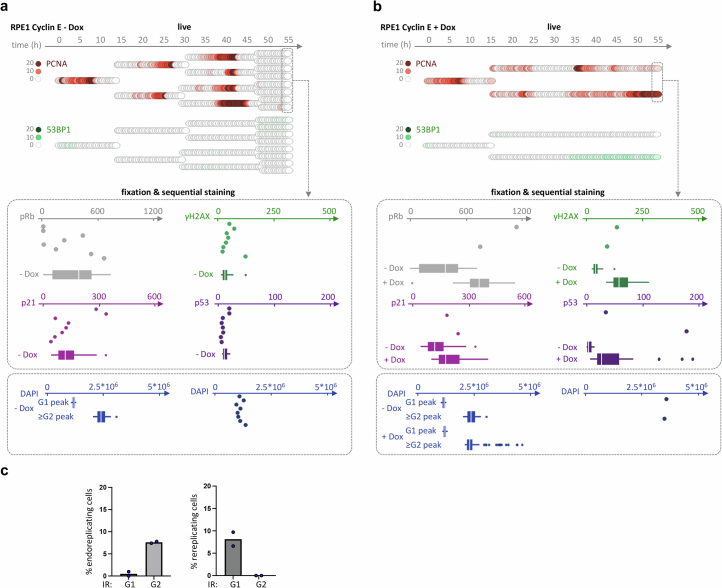
Re- and endo-replication upon oncogene overexpression. (**a**) Single cell lineage from control 53BP1-PCNA RPE-1 cells without Dox-induced Cyclin E1 expression. PCNA patterns and 53BP1 foci are depicted. Sequential staining intensities from individual cells of the lineage and mean intensities from the corresponding reference population are depicted for the markers pRB, γH2AX, p21 and p53. The total DAPI intensity of the nucleus from the corresponding reference population is depicted as well as from the single cells at the end of live imaging. (**b**) Single cell lineage from 53BP1-PCNA RPE-1 cells with Dox-induced Cyclin E1 expression (100 ng/ml Doxycycline for 24 h). Box plot limits indicate 25th percentile (Q1) and 75th percentile (Q3); boxes represent interquartile range (IQR, Q3-Q1) with medians (solid lines). Whiskers define lower and upper adjacent value; dots show outliers smaller than Q1 – 1.5 × IQR and greater than Q3 + 1.5 × IQR. (**c**) Analysis of endo- and re-replication frequency in asynchronously growing 53BP1-PCNA U-2 OS cells that received IR (4 Gy) either in G1 or in G2. Means and individual values from two independent live cell experiments are shown. Source data

Next, to address whether activation of common oncogenes might induce similar patterns of phenotypic heterogeneity and polyploidization, we generated cell lines that, in addition to expressing endogenously tagged 53BP1 and PCNA, overexpress oncogenic H-RAS V12 (HRAS) or cyclin E1 (Extended Data Fig. 10a,b). As expected, overexpression of HRAS or cyclin E1 induced replication stress, which was associated with reduced replication fork speed and an increase in micronuclei formation (Extended Data Fig. 10c,d). Using these cell lines, we performed single-cell tracking and lineage analysis over 70 h, covering up to four cell generations (Extended Data Fig. 10e). Oncogene overexpression not only markedly increased cellular heterogeneity and sister cell asymmetry compared with the control, but also resulted in enhanced polyploidization (Fig. 5a and Extended Data Fig. 10e–h). Induction of polyploidy in HRAS and cyclin E1 cells was further increased by IR (Extended Data Fig. 11a–d). Supporting their proliferative potential, polyploid cells showed EdU incorporation (Extended Data Fig. 11d) and could be followed by live-cell microscopy through multiple rounds of cell division (Extended Data Fig. 11e). Notably, oncogene-induced polyploidization occurred by either rereplication or endoreplication, similar to pevonedistat-treated cells (Extended Data Fig. 11f). Consistently, polyploidization was observed in RPE-1 cells with inducible cyclin E1 (Extended Data Figs. 11g,h and 12a,b). Taken together, these results suggest that routes towards polyploidization in interphase involve both rereplication and endoreplication induced by the same oncogenic triggers. We noticed that endoreplication in HRAS- and cyclin-E1-overexpressing cells was associated with increased DNA damage, marked by elevated 53BP1 foci, after the first and before the second round of DNA replication (Fig. 5a). Similarly, cells that were irradiated in G2 showed more frequent endoreplication compared with cells that were irradiated in G1, whereas the opposite was true for replication patterns indicative of rereplication (Extended Data Fig. 12c). DNA damage experienced early in the cell cycle therefore increases the risk of replication stress and rereplication in the ensuing S phase, while DNA damage experienced late in the cell cycle increases the risk of endoreplication.

Having observed that both routes cause aberrant genome duplication yet by different mechanisms and at different costs for genome integrity, we investigated differential gene expression patterns among polyploid cells. To this end, we performed fluorescence-activated cell sorting (FACS) for scRNA-seq analysis of polyploid and non-polyploid (2N–4N) cells after pevonedistat treatment or HRAS overexpression, comparing them to non-polyploid empty vector (EV) control cells (Supplementary Fig. 9a). Reassuringly, the polyploid HRAS and pevonedistat-treated cells segregated from the EV and their non-polyploid (2N–4N) control populations (Fig. 5b). For both, polyploid HRAS and pevonedistat-treated cells, differentially regulated genes were identified (Supplementary Fig. 9b,c and Supplementary Tables 1 and 2). Despite the difference in treatment, 150 differentially regulated genes were common between the HRAS and pevonedistat-treated polyploid cells (Supplementary Fig. 9d,e). We then performed a subcluster analysis of the HRAS polyploid cells (Fig. 5c). Pairwise analysis of differential gene expression between the subclusters followed by Gene Ontology (GO) analysis revealed cell cycle regulation and chromosome segregation during mitosis as well as DNA replication and DNA damage signalling consistently as the most highly enriched GO terms, in agreement with these processes being relevant for polyploidization (Supplementary Fig. 10a–c). Performing the same subcluster analysis for pevonedistat-treated polyploid cells yielded very similar results with the same highly enriched GO terms (Supplementary Fig. 10d–f). We therefore focused on the overlap between the HRAS and pevonedistat-treated polyploid samples and particularly on the four subclusters with the strongest differential gene expression (Supplementary Fig. 11). Among those four subclusters, 148 differentially regulated genes were identified using a cut-off of 0.001 for false-discovery rate (FDR) and *P* value and a fold-change (FC) cut-off of 1.5 (Supplementary Fig. 12a and Supplementary Table 3). A GO analysis of this group of genes yielded terms related to cell cycle regulation and chromosome segregation as the most significantly enriched terms (Supplementary Fig. 12b). Similar results were obtained from an independent replicate scRNA-seq analysis (Supplementary Fig. 12c–f). Using more stringent criteria for FC, FDR and *P* values (FC > 4, FDR < 0.00001, *P* < 0.00001), we extracted 28 genes from the original list of 148 genes, which were most significantly deregulated among the subclusters (Supplementary Table 4). These 28 genes were still enriched for GO terms related to cell cycle and mitotic cell division (Fig. 5d) and showed high connectivity in functional network analysis (Fig. 5e). In total, 22 of these 28 genes were identified in the independent replicate scRNA-seq analysis (Supplementary Table 4). At the centre of this network are the cyclin-dependent kinase CDK1, cyclin A, aurora kinase B (AURKB) and topoisomerase II alpha (TOP2A) (Fig. 5e and Supplementary Fig. 12g,h). To further explore the functional relevance of this network, we analysed nuclear cyclin A expression at protein level using QIBC and observed distinct subpopulations among polyploid cells with high/low nuclear cyclin A levels (Fig. 5f). Moreover, while pevonedistat induced both endoreplication and rereplication (with roughly identical shares), TOP2A inhibition by etoposide induced predominantly rereplication, whereas CDK1 inhibition by RO3306 caused mainly endoreplication (Fig. 5g). These differences were reflected by the cell cycle patterns of chromatin-bound MCM2, MCM4 and MCM7, constitutive components of the replicative helicase MCM2-7: while etoposide caused heterogenous MCM chromatin loading in S phase and G2, consistent with rereplication during S phase and endoreplication after S-phase completion, CDK1 inhibition by RO3306 primarily caused new loading of MCM in late S/G2, consistent with endocycles (Supplementary Fig. 12i–l). Thus, the identified gene network has functional implications for polyploidization by endoreplication versus rereplication.

## Discussion

Cellular heterogeneity is widespread in nature, yet how it emerges remains poorly understood. Stochasticity is involved in replication origin activation, and multiprotein complexes like the replisome show inherent stochastic behaviour at the molecular level^46^. Moreover, sister cell heterogeneity after replication stress experienced in the previous cell cycle may result from mosaic inheritance of new DNA synthesized by leading- and lagging-strand synthesis, respectively^47^. Acute DNA damage experienced in G1 also induced sister cell heterogeneity in the next cell generation, including marked differences in S-phase commitment. Although strand-specific DNA lesion segregation, as previously observed for replication-associated damage, DNA single-strand breaks, UV-induced lesions and chemotherapy-induced DNA damage^15,16,48–52^, can contribute to sister cell heterogeneity, additional mechanisms probably exist. One possibility is the sequestration of proteins with dual functions in DNA damage repair and replication at DSBs, therefore limiting their availability at replication forks after S-phase entry, which in turn could trigger heritable strand-specific changes. Indeed, 53BP1 and its upstream regulators RNF8 and RNF168 are limiting for the response to DSBs^53^, and they are also involved in replication fork protection^54,55^. Conversely, sequestration of RNF8, RNF168 and 53BP1 at replication-stress-associated heritable DNA lesions in G1 can limit their ability to respond to acute DSBs, and asymmetric distribution of these proteins or their mRNAs during mitosis may amplify such limitations in individual sister cells.

Moreover, DNA damage-induced chromatin changes may have a more long-lasting effect on genome organization. Although controlled DSB induction in primary mouse cells did not cause persistent transcriptional repression^56^, chromatin alterations after completion of DSB repair in HeLa cells were recently found to manifest as heritable impairments of gene expression^57^. Moreover, changes in gene expression after micronucleus formation can become heritable after reincorporation of micronuclear DNA into the main daughter cell nucleus^58^. Similarly, DNA-damage-induced changes in genome organization, beyond the process and duration of genome repair, might affect replication timing in the next cell generation. Considering that replication stress is a major source of endogenous DNA damage, a transformative loop from replication perturbations to DNA damage-induced stable chromatin changes (epigenetic scars) might be induced, which could alter transcription programs and replication timing in the next cell cycle, thereby promoting further replication stress and fuelling cellular plasticity. Possible implications are that transient DNA lesions may have long-lasting effects on genome function through inducing and amplifying cellular heterogeneity.

## Methods

### Cell culture and treatments

All cell lines were grown at 37 °C under standard cell culture conditions (humidified atmosphere, 5% CO_2_) in Dulbecco’s modified Eagle’s medium (DMEM, Gibco) containing 10% FBS (Corning) and 1% penicillin–streptomycin antibiotics. Stable endogenously tagged U-2 OS PCNA–mEmerald 53BP1–mScarlet cells were maintained in the presence of 4 µg ml^−1^ blasticidin (InvivoGen) and 500 µg ml^−1^ geneticin (Gibco). Stable endogenously tagged U-2 OS 53BP1–mScarlet cells^33^ were maintained in the presence of 400 µg ml^−1^ geneticin (Gibco). Stable U-2 OS PCNA–mEmerald 53BP1–mScarlet cells with overexpression of HRAS or cyclin E1 and their empty vector controls were maintained in the presence of 4 µg ml^−1^ blasticidin (InvivoGen), 500 µg ml^−1^ geneticin (Gibco) and 100 µg ml^−1^ hygromycin B (Invitrogen). RPE-1 PCNA–mEmerald 53BP1–mScarlet cells were maintained in the presence of 4 µg ml^−1^ blasticidin (InvivoGen) and 500 µg ml^−1^ geneticin (Gibco). Inducible cyclin E1 RPE-1 cells expressing endogenously tagged PCNA–mEmerald and 53BP1–mScarlet were maintained in the presence of 4 µg ml^−1^ blasticidin (InvivoGen), 500 µg ml^−1^ geneticin (Gibco) and 0.5 µg ml^−1^ puromycin (Thermo Fisher Scientific). U-2 OS 53BP1-GFP AID-DIvA cells^40,59^ were maintained in the presence of 1 mM sodium pyruvate (Sigma-Aldrich) and 800 µg ml^−1^ geneticin (Gibco) and 1 µg ml^−1^ puromycin (Thermo Fisher Scientific). U-2 OS CDC45-GFP MCM4-Halo cells^60^ were maintained in the presence of 1 mM sodium pyruvate (Sigma-Aldrich) and the HaloTag ligand JF549 (Promega) was added at a concentration of 200 nM for a duration of 20 min before fixation. U-2 OS and RPE-1 cells were reauthenticated by short tandem repeat (STR) profiling in 2025 with 100% match and no detectable contamination (RRID: CVCL_0042 and CVCL_4388). All cell lines used in this study were grown in sterile conditions and routinely tested for mycoplasma.

The following compounds were used in this manuscript: aphidicolin (Sigma-Aldrich), ATRi (AZ-20, TOCRIS), CDKi RO3306 (Sigma-Aldrich), pevonedistat (MLN4924, Selleckchem), etoposide (Selleckchem), 4-OHT (Sigma-Aldrich), IAA (Sigma-Aldrich) and doxycycline (Sigma-Aldrich). Transfections were performed with Ambion Silencer or Silencer Select siRNAs using Lipofectamine RNAiMAX (Thermo Fisher Scientific) at a final concentration of 25 nM. Negative Silencer Select control NEG1 from Ambion was used as a non-targeting control. The following siRNAs were used (5′–3′): *TP53BP1* (s14313; GAAGGACGGAGUACUAAUATT); *GMNN* (134697; GGAGUCAUUUGAUCUUAUGTT); *CDKN1A* (s416; GCACCCUAGUUCUACCUCATT); *TP53* (s606; GAAAUUUGCGUGUGGAGUATT); and *AMBRA1* (s31112; GCUCAACAAUAACAUUGAATT).

### Cloning

Cloning was done using chemically competent bacteria generated in-house, derived from Library Efficiency DH5a competent cells (Thermo Fisher Scientific). Primer sequences are provided separately (Supplementary Table 5). Correct cloning and integration into target vectors was confirmed by Sanger sequencing (Microsynth).

### Cloning of components for endogenous PCNA tagging

The mEmerald-P2A-BlasticidinR pUC18 template was cloned by a three-piece Gibson assembly. The pUC18 vector was linearized by PCR using primers pUC18_lin_fwd and pUC18_lin_rev. The *mEmerald* gene was amplified by two rounds of PCR from the mEmerald-C1 plasmid template (Addgene, 53975) using first primers mEm_GA_fwd and mEm_P2A_rev and the product amplified with GA_pUC18_fwd and mEm_P2A_rev. The blasticidin gene was amplified with primers BlastR_P2A_fwd and BlastR_pUC18_rev. The individual pieces were purified by gel extraction and assembled with Gibson assembly followed by transformation of the product, isolation of plasmids and verification by sequencing.

The sgRNA duplex to target Cas9 endonuclease in pX459 (Addgene, 48139) to the endogenous PCNA C-terminal near the stop codon was generated as described^61^. In brief, primers PCNA_sgRNA_top and PCNA_sgRNA_bot were phosphorylated with T4 phosphonucleotide kinase (NEB) at 37 °C for 30 min followed by a temperature gradient from 95 °C to 25 °C decreasing 5 °C min^−1^. The product was diluted 100-fold in water and assembled into the vector by Golden Gate assembly using BbsI (NEB) and T4 DNA ligase (NEB) through 12 cycles between 5 min at 37 °C and 5 min at 16 °C followed by transformation, plasmid isolation and sequencing verification of correct insertion.

The repair template for tagging endogenous PCNA by homology-directed repair was amplified by PCR from the mEmerald-P2A-BlasticidinR pUC18 plasmid generated as described above using Q5 (NEB) polymerase amplification with primers PCNA_HDR_mEm_fwd and PCNA_mEm_blst_rev followed by PCR purification with a QIAquick PCR purification column according to the manufacturer’s instructions.

### Endogenous 53BP1–mScarlet and PCNA–mEmerald tagging in U-2 OS and RPE-1 cells

U-2 OS cells with endogenously tagged 53BP1–mScarlet were described previously^33^. RPE-1 cells with endogenously tagged 53BP1–mScarlet were generated in the same manner. These cell lines were then used as the starting point for endogenous tagging of PCNA with the monomeric green fluorescent protein mEmerald. A total of 40,000 cells was seeded into two wells of a six-well plate one day before transfection. The PCR amplified PCNA mEmerald-P2A-BlasticidinR HDR template and the pX459 Cas9 plasmid generated as described above were used for transfection. Then, 1 µg of the HDR template and 1 µg of the pX459 plasmid were diluted with 250 µl OptiMEM (Invitrogen) and mixed with 6 µl TransIT-LT1 transfection reagent (Mirus). pX459 without the HDR template was prepared similarly as a negative control. This was allowed to stand 15 min before adding it to the cells. Next, 24 h later, cells were transferred to 15 cm dishes. Then, 48 h after transfection, blasticidin (InvivoGen) was added to a final concentration of 4 µg ml^−1^ for selection the next 7 days. After selection and death of cells transfected without the HDR template, individual colonies were picked by trypsin detachment in cloning cylinders and transferred to a 96-well plate (Greiner µ-clear) for expansion and validation of the presence of green fluorescence. Clones with fluorescence were further expanded and cells collected for genomic PCR with the primers PCNA_genCterm_fwd and PCNA_genCterm_rev to confirm the insertion of DNA with a size corresponding to the mEmerald-P2A-BlasticidinR module by agarose gel electrophoreses imaged on Infinity ST5 Xpress v16.16d. To visualize endogenous 53BP1 and endogenous PCNA with minimal interference of their cellular functions, monoallelic targeting was considered sufficient. The mEmerald-C1 plasmid was a gift from M. Davidson (Addgene, 53975), pSpCas9(BB)-2A-Puro (PX459) was a gift from F. Zhang (Addgene, 48139)^61^, pmScarlet-i_C1 was a gift from D. Gadella (Addgene, 85044)^62^.

### Cloning of pBABE_EV and pBABE_HRAS V12

For retroviral transduction, retroviral vectors pBABEneo-HRASV12, a gift from J. Debnath (Addgene, 71304)^63^ and pBABEneo were used. For them to be compatible with the stable PCNA–mEmerald 53BP1–mScarlet U-2 OS cells, the resistance of the pBABE plasmids was exchanged from neomycin to hygromycin. The pBABE_EV (EV = empty vector) and pBABE_HRAS V12 backbones were amplified by PCR amplification using primers Backbone_fwd and Backbone_rev while adding AscI and MfeI restriction sites. The HygroR insert was also generated by PCR amplification using primers Hygro_fwd and Hygro_rev, adding AscI and MfeI restriction sites. This was followed by incubation with DpnI (NEB) for 2.5 h at 37 °C. PCR-cleanup was done according to the manufacturer’s instructions (QIAquick PCR Purification Kit, Qiagen). Both the backbones and HygroR inserts were incubated with AscI (NEB) for 10 h, followed by heat inactivation of the restriction enzyme at 80 °C for 20 min. This was followed by incubation with MfeI (NEB) for 10 h at 37 °C. Backbones were dephosphorylated using rSAP (NEB) for 1 h followed by gel purification (QIAquick Gel Extraction Kit, Qiagen). The ligation of the backbones and HygroR inserts was done at 16 °C for 16 h. Transformation was performed using chemically competent DH5α generated in house, derived from Library Efficiency DH5α competent cells (Thermo Fisher Scientific). Correct sequences were confirmed by control digestion and sequencing.

### Cloning of pBABE_cyclin E1

The pBABEneo-cyclin E1 plasmid for retroviral transduction was a gift from P. Janscak (University of Zurich). To exchange the resistance from neomycin to hygromycin, the pBABE_cyclin E1 plasmid was linearized using primers pBABE_lin_fwd and pBABE_lin_rev. The insert was generated using the primers pBABE_Gibson_fwd and pBABE_Gibson_rev. After gel extraction, Gibson assembly and transformation were performed. Correct sequences were confirmed by control digestion and sequencing.

### Cloning of cyclin E1 plasmid for inducible expression

The pLVX-TetONE plasmid was linearized using the primers pLVX-TetONE_lin_rev and pLVX-TetONE_lin_fwd and the insert for cyclin E1 was amplified with primers pLVX-TetONE_to_cyclinE1_GA_amp_fwd and pLVX-TetONE_to_cyclinE1_GA_amp_rev. After gel extraction, Gibson assembly and transformation were performed. Correct insertion was confirmed by control digestion and sequencing.

### Generation of oncogene-overexpressing cell lines

For the generation of U-2 OS PCNA 53BP1 cells with overexpression of HRAS V12 or cyclin E1, the U-2 OS PCNA 53BP1 cells were used for retroviral transduction. For this, HEK293T Phoenix retrovirus producer cells for transduction were prepared by plating them 48 h before infection in DMEM (Gibco) containing 10% FBS (Corning) and 1% penicillin–streptomycin antibiotics (Gibco). For transduction, chloroquine was added to each plate of HEK293T Phoenix cells at a final concentration of 20 µM 1 h before transduction. For the formation of the calcium-phosphate-DNA co-precipitate deionized water, 10 µg DNA and CaCl_2_ (Sigma-Aldrich) at a final concentration of 1.25 M were mixed for a final volume of 500 µl per 10 cm plate. This mix was added dropwise to 500 µl 2× HBS (50 mM HEPES (ChemieBrunschwig), 10 mM KCl (Sigma-Aldrich), 12 mM dextrose (Sigma-Aldrich), 280 mM NaCl (Merck), 1.5 mM Na_2_HPO_4_·7H_2_O (Merck)) solution while vortexing vigorously. After incubating for 5 min, the H_2_O/DNA/CaCl_2_/HBS mix was added dropwise to the HEK293T Phoenix cells and then gently distributed. After 6 h of incubation at 37 °C under standard cell culture conditions (humidified atmosphere, 5% CO_2_), the medium was exchanged to fresh growth medium. Then, 48 h after transduction, the supernatant from the transfected HEK293T Phoenix cells was collected and centrifuged and the supernatant transferred to a new tube. Polybrene (Sigma-Aldrich) was added to the supernatant at a final concentration of 8 µg ml^−1^. After removal of the medium from the target cells, the viral supernatant was added. After 3 h, the infection was repeated with fresh viral supernatant. After 24 h, the viral supernatant was exchanged to selection medium (DMEM containing 10% FBS (Corning), 1% penicillin–streptomycin antibiotics (Gibco), 4 µg ml^−1^ blasticidin (InvivoGen), 500 µg ml^−1^ geneticin (Gibco) and 100 µg ml^−1^ hygromycin B (Invitrogen)). After selection, the expression levels and functionality were validated by immunofluorescence staining and western blot analysis.

For the generation of RPE-1 cells with inducible overexpression of cyclin E1, RPE-1 cells expressing endogenously tagged PCNA–mEmerald and 53BP1–mScarlet were subjected to lentiviral transduction. For this, a DNA/CaCl_2_/H_2_O mix was prepared by mixing 6 µg of LTR plasmid and 6 µg of pCDN-LBH plasmid with 12 µg of oncogene-encoding plasmid with CaCl_2_ at a final concentration of 250 mM in H_2_O. To this mixture, 500 µl 2× HBS (50 mM HEPES (ChemieBrunschwig), 10 mM KCl (Sigma-Aldrich), 12 mM dextrose (Sigma-Aldrich), 280 mM NaCl (Merck), 1.5 mM Na_2_HPO_4_·7H_2_O (Merck)) was added dropwise, while vortexing. After incubating the precipitate for 5 min at room temperature, it was added to HEK293T cells and left overnight at 37 °C and 5% CO_2_. The next day, the medium was replaced with fresh medium and cells were incubated again overnight. The virus was then collected on two consecutive days, the fractions were pooled and filtered through a 0.45 µm filter and stored at 4 °C. The viral supernatant was diluted with medium 1:2 and added together with 8 µg ml^−1^ polybrene to the target cells. After overnight incubation, the virus-containing cell supernatant was replaced with fresh medium and cells were incubated for 48 h. Then, puromycin was added at a final concentration of 0.5 µg ml^−1^ for selection. After selection, the expression levels and functionality were validated by immunofluorescence staining and western blot analysis.

### Live-cell imaging and treatments

On the day before imaging, cells were seeded in Fluorobrite DMEM (Thermo Fisher Scientific, A1896701) supplemented with 10% FBS (Corning), penicillin and streptomycin (Gibco) and GlutaMAX (Gibco) into 96-well µ-plates (Ibidi, 89626). In the case of RPE-1 PCNA–mEmerald 53BP1–mScarlet cells, before cell seeding, the 96-well imaging plates were coated with collagen diluted in water (1:50) (PureCol-S, 5015-20ML, Advanced Biomatrix) according to the protocol provided by the company. The plate was sealed with a Breathe-Easy sealing membrane (Sigma-Aldrich, Z380059). Live-cell imaging of cells was done using a previously described automated widefield GE InCell Analyzer 2500HS high-content screening microscope^33^ with environmental control for gas (5% CO_2_ and 20% O_2_) and temperature (37 °C) using the GE InCell Analyzer 2500 V7.4 acquisition software. The system contains a seven-colour solid-state illuminator (SSI), a PCO-sCMOS camera system (16 bit, 2,048 × 2,048 pixel, pixel size 6.5 × 6.5 μm, readout speed: 272 MHz), two quad band-pass polychroic mirrors and single band-pass emission filters. For Live+QIBC experiments, the polychroic beam splitter BGOFR_1 (blue (excitation BP 390/22, emission BP 435/48), green (excitation, BP 473/28; emission, BP 511/23), orange (excitation, BP 542/30; emission, BP 597/45), far red (excitation, BP 631/28; emission, BP 684/24)) and a CFI Plan Apo Lambda ×20 Air objective (NA 0.75, WD 1.0 mm) with hardware autofocus were used. Single-plane widefield images were acquired at 100 ms exposure for green and 300 ms exposure for orange; no binning was performed. Nine fields per well were acquired at 30 min intervals for up to 72 h, with typically 2–3 wells per experimental condition and at least 3,000 cells seeded per well.

To irradiate the cells, the plate acquisition was temporarily paused for irradiation at 130 kvp for 33 s per 1 Gy of irradiation in a Faxitron Cabinet X-ray System Model RX-650. Unless indicated otherwise, irradiation was performed with 4 Gy. The addition of drugs was done likewise. At the end of the live imaging, cells were washed once with PBS followed by fixation with 4% formaldehyde (Sigma-Aldrich) in PBS for 18 min, before being washed once more with PBS followed by storage at 4 °C until multiplex staining.

### Multiplex staining

Cells fixed after live-cell imaging were processed for iterative indirect immunofluorescence imaging (4i) as described previously^35^. The cells were permeabilized with 0.5% Triton X-100 (Sigma-Aldrich) for 5 min followed by washing with PBS. Cells were then stained with DAPI (Thermo Fisher Scientific) for 10 min, washed with PBS and images of the fixed cells with endogenous labels and DAPI were acquired. The signals from the fluorescent proteins were removed by denaturation with elution buffer (0.5 M glycine (BioSolve Chemicals), 3 M urea (Eurobio Scientific), 3 M guanidine hydrochloride (Sigma-Aldrich), 50 mM TCEP (Sigma-Aldrich) in water, pH 2.5) for 10 min, 2 times, followed by three washes with double-distilled H_2_O. Images were acquired to control for the loss of signal. The cells were then blocked and free cysteines were conjugated by incubation with sBS buffer (1% bovine serum albumin (Sigma-Aldrich), 150 mM maleimide (Sigma-Aldrich) in PBS, pH 7.4) for 1 h. After two washes with PBS the cells were incubated with 100 μl cBS (1% BSA (Sigma-Aldrich) in PBS, pH 7.4) with primary antibodies anti-γH2AX (1:1,000, mouse, BioLegend 613402) and anti-pRb (1:500, rabbit, Cell Signaling Technologies, 8516S) for 2 h. After two PBS washes, cells were incubated with 100 µl cBS with secondary antibodies anti-mouse A488 (1:500, Thermo Fisher Scientific, A11029) and anti-rabbit A647 (1:500, Thermo Fisher Scientific, A21245) for 1 h. Finally, cells were stained with DAPI. Elution and acquisition of images after elution was repeated as above, followed by sBS blocking for 1 h and washing. Then cells were stained with anti-p21 (1:500, rabbit, Abcam, ab109520) and anti-p53 (1:500, mouse, Thermo Fisher Scientific, AHO0152) for 2 h. After two PBS washes, cells were incubated with anti-mouse A488 (1:500, Thermo Fisher Scientific, A11029) and anti-rabbit A647 (1:500, Thermo Fisher Scientific, A21245) for 1 h. For every staining or elution round, imaging buffer (700 mM *N*-acetyl-cysteine (Sigma-Aldrich) in double-distilled H_2_O, pH 7.4) was added before imaging. Imaging was performed using the automated widefield GE InCell Analyzer 2500HS high-content screening, which was also used for live-cell imaging (seven-colour SSI, PCO-sCMOS camera system (16 bit, 2,048 × 2,048 pixel, pixel size: 6.5 × 6.5 μm, readout speed: 272 MHz), two quad band-pass polychroic mirrors, single band-pass emission filters). The polychroic beam splitter BGOFR_1 (blue (excitation BP 390/22, emission BP 435/48), green (excitation BP 473/28, emission BP 511/23), orange (excitation BP 542/30, emission BP 597/45), far red (excitation BP 631/28, emission BP 684/24)) and a CFI Plan Apo Lambda ×20 Air objective (NA 0.75, WD 1.0 mm) were used, with hardware laser autofocus for acquisition of single plane images. No binning was performed. Nine fields per well were acquired, with typically 2–3 wells per experimental condition and at least 3,000 cells seeded per well. For all imaging and staining rounds the acquisition settings were kept constant.

### Analysis of live-cell imaging with 4i acquisitions after fixation

#### Conversion of images from live-cell acquisition to multi-parametric time-correlated readouts

Images from the live imaging were processed using a time-lapse script (https://github.com/AltmeyerLab/SingleCellTracking_Timelapse), which involves generation of .tif stacks and generation of Olympus ScanR Analysis (v.3.0.1, 3.2 and 3.3.0) compatible images. This was followed by custom conversion in Olympus ScanR Analysis software (v.3.0.1, 3.2 and 3.3.0) to generate the required metadata and the experiment file. Segmentation of cells was performed on a smoothened mask of the PCNA channel. Parameters for total and mean intensities of the two channels, nuclear area (1 pixel ≅ 0.1056 µm^2^), circularity factor, perimeter, elongation factor, foci counts for PCNA and 53BP1, integrated intensities of foci in each channel, time slices, well number, position, centre *x* coordinate and centre *y* coordinate were extracted and tabulated for each cell in every timeframe.

#### Alignment and analysis of 4i acquisitions

For alignment of 4i imaging data, an alignment script was used (https://github.com/AltmeyerLab/SingleCellTracking_Multiplex-Alignment), which renders images from multiple rounds of staining compatible with downstream analysis using the Olympus ScanR Analysis software (v.3.0.1, 3.2 and 3.3.0). Custom conversion and image analysis was performed with the Olympus ScanR Analysis software (v.3.0.1, 3.2 and 3.3.0). In addition to the parameters outlined above, the mean and total intensities for all stainings as well as γH2AX foci counts and integrated intensities of γH2AX foci were extracted and tabulated for each cell.

#### Lineage-based tracking of live cells

Tables with data from a field of view from the post-fixation 4i acquisition were appended to data from the live-cell imaging for the same field of view. The frame numbers and *x*–*y* coordinates were used as the basis for a previously published MATLAB (MathWorks, MATLAB R2019b, R2020b, R2023a) script to automatically generate live-cell tracks for each cell. The same script along with the ImageJ/Fiji 64-bit (v.1.53f, 1.53t, 1.54f, 1.54m) plugin ‘Manual Tracking’ was used together with the corresponding image stacks to visualize, correct and manually reassign cells for lineage-based analysis. After reassignment, MATLAB scripts (both scripts are available at GitHub; https://github.com/AltmeyerLab/MatlabTracking/; script 1, Cell_tracking.m; and script 2, KeyDataExtractions.m) were used to generate lineage assignments and data from curated and reassigned cells was written into .csv files and loaded into TIBCO Spotfire (v.7.9.1, 10.10.1) for visualization of the lineage trees together with the post-fixation 4i data. For U-2 OS cells, at least 20 individual cell lineages per condition, corresponding to up to 80 granddaughter cells, were tracked. For RPE-1 cells, at least ten individual cell lineages per condition, corresponding to up to 40 granddaughter cells, were tracked. For all extended live-cell imaging experiments with lineage tracking and multiplex staining (Live+QIBC), at least two independent biological replicate experiments were performed. Area measurements are shown in pixel with 1 pixel ≅ 0.1056 µm^2^. In some instances, although the median and the complete Q1–Q3 range are shown for whole-population box plots associated with Live+QIBC results, not all individual values are displayed to allow for visualization of population effects with consistently scaled axes.

### Cell cycle analysis and analysis of sister cell heterogeneity

After validating that PCNA foci were mostly confined to EdU-positive S-phase cells, thresholds were established for S-phase categorization as well as for G1–S and S–G2 transitions based on live-cell tracking of untreated control cells. In brief, unchallenged cells transitioned from G1 to S phase quickly (typically within a 30-min interval), indicated by a marked increase in PCNA foci (≥10 foci). Likewise, unchallenged cells transitioned from S phase to G2 quickly (typically within 1–2 30-min intervals), indicated by a marked decrease in PCNA foci (≤4 foci). Thresholds were therefore defined as follows: 0–4 PCNA foci for G1; ≥10 PCNA foci for ≥2 consecutive timepoints for S phase (G1–S transition); ≤4 PCNA foci for ≥2 consecutive timepoints for G2 phase (S–G2 transition). Periods in between were considered transition periods.

53BP1 foci heterogeneity was evaluated on the basis of the endogenous 53BP1 foci in the F_1_ generation measured from the live imaging data. For the other markers, heterogeneity was evaluated from multiplexed end-point measurements. Three categories of sister cell heterogeneity were introduced based on the differences in foci counts or nuclear intensity levels; thresholds were dependent on the individual experiment: for Extended Data Fig. 5e, 53BP1 foci heterogeneity was categorized as follows: low (0–1 focus difference), medium (2–4 foci difference), high (>4 foci difference). γH2AX fluorescence intensity was categorized as follows: low (0–100 a.u. difference), medium (100–200 a.u. difference), high (>200 a.u. difference). p53 fluorescence intensity was categorized as follows: low (0–50 a.u. difference), medium (50–100 a.u. difference), high (>100 a.u. difference). p21 fluorescence intensity was categorized as follows: low (0–100 a.u. difference), medium (100–500 a.u. difference), high (>500 a.u. difference). For Extended Data Fig. 8i, 53BP1 foci heterogeneity was categorized as follows: low (0–1 focus difference), medium (2–4 foci difference), high (>4 foci difference). For γH2AX fluorescence intensity, heterogeneity was categorized as follows: low (0–50 a.u. difference), medium (50–100 a.u. difference), high (>100 a.u. difference). For p53 fluorescence intensity, heterogeneity was categorized as follows: low (0–200 a.u. difference), medium (200–500 a.u. difference), high (>500 a.u. difference). For pRb fluorescence intensity, heterogeneity was categorized as follows: low (0–500 a.u. difference), medium (500–1,000 a.u. difference), high (>1,000 a.u. difference). For Supplementary Fig. 8b, 53BP1 foci heterogeneity was categorized as follows: low (0–1 focus difference), medium (2–4 foci difference), high (>4 foci difference).

### High-content microscopy and QIBC

Automated multichannel widefield microscopy for high-content imaging and QIBC was performed using the Olympus ScanR High-Content Screening System as described previously^33,64^. The system is equipped with an inverted motorized Olympus IX83 microscope, a motorized stage, IR-laser hardware autofocus, a fast emission filter wheel with one set of band-pass filters for multi-wavelength acquisition (DAPI (excitation BP 395/25 or BP 390/22, emission BP 435/26), FITC (excitation BP 470/24 or BP 475/28, emission BP 515/30), TRITC (excitation BP 550/15 or BP 555/28, emission BP 595/40), Cy5 (excitation BP 640/30 or BP 635/22, emission BP 705/72)), and a Hamamatsu ORCA-FLASH 4.0 V2 sCMOS camera (12 bit, 2,048 × 2,048 pixel, pixel size 6.5 × 6.5 μm) with a ×40 UPLSAPO (NA 0.9, WD 0.18 mm), a ×20 UPLSAPO (NA 0.75, WD 0.6 mm) and a ×10 UPLSAPO (NA 0.4, WD 3.1 mm) air objective. Images of cell populations were acquired under non-saturating conditions (Olympus ScanR Image Acquisition software (v.3.0.1, 3.2 and 3.3.0)), typically 25 (5 × 5) to 81 (9 × 9) images per well, depending on the objective and cell density, and identical settings were applied to all samples within one experiment. Hardware and software autofocus on the DAPI channel were used. No binning was performed. Images were analysed using the inbuilt Olympus ScanR Analysis software (v.3.0.1, 3.2 and 3.3.0), a dynamic background correction was applied and nucleus segmentation was performed using an integrated intensity-based object detection module based on the DAPI signal. Downstream analyses were focused on properly detected nuclei containing a 2N–4N DNA content as measured by total and mean DAPI intensities, unless increased ploidy in cells with >4N DNA content was also analysed. Fluorescence intensities were quantified and are depicted as arbitrary units. Colour-coded scatterplots of asynchronous cell populations were generated with TIBCO Spotfire (v.7.9.1, 10.10.1). Within one experiment, similar cell numbers were compared for the different conditions. For visualizing discrete data in scatterplots, mild jittering (random displacement of data points along discrete data axes) was applied to demerge overlapping datapoints. Representative scatterplots and quantifications of independent experiments, typically containing several thousand cells each, are shown.

### DNA fibre imaging

A Leica THUNDER (Las X 3.7.6.25997) Imager 3D Live Cell system was used for DNA fibre analysis. The system is equipped with a Leica DMi8 inverted widefield microscope, a motorized stage, a Leica LED8 light source (390 nm, 440 nm, 475 nm, 510 nm, 555 nm, 575 nm, 635 nm, 747 nm) and a Leica monochrome fluorescence DFC9000 GTC sCMOS camera (12/16 bit, 2,048 × 2,048 pixel, pixel size: 6.5 × 6.5 μm). Dual-labelled DNA fibres (Alexa Fluor 488 and 555) were imaged with the 475 nm and 555 nm LEDs, the DFT5 Quad filter (excitation filters: 375–407, 462–496, 542–566, 622–654; main beam splitter: 415, 500, 572, 660; emission filters: 420–450, 506–532, 581–607, 666–724), additional emission filters 535/70 and 642/80 from an external clean-up filter wheel, and a Leica HC PL APO CS2 (NA 1.4, WD 0.14 mm) ×63 oil objective.

### Immunostaining

For standard immunofluorescence staining, high-content microscopy and QIBC analyses, cells were seeded on sterile 12 mm glass coverslips or 96-well plates and were allowed to proliferate until they reached a cell density of 70–90%. Cells were then fixed in 3% formaldehyde (Sigma-Aldrich) for 15 min at room temperature, washed once in PBS, permeabilized for 5 min at room temperature in 0.2% Triton X-100 (Sigma-Aldrich) in PBS, washed twice in PBS and incubated in blocking solution (filtered DMEM containing 10% FBS (Corning) and 0.02% sodium azide (Merck)) for 15 min at room temperature. When the staining was combined with an EdU Click-iT reaction, the reaction was performed before the incubation with the primary antibody according to manufacturer’s recommendations (Thermo Fisher Scientific). Where indicated, cells were pre-extracted in 0.2% Triton X-100 (Sigma-Aldrich) in PBS for 2 min on ice before formaldehyde fixation. Denaturing, where indicated, was performed in 2.5 M HCl for 10 min. All primary antibodies were diluted in blocking solution and incubated for 2 h at room temperature. Secondary antibodies (Alexa Fluor 488, 555, 568, 647 anti-mouse, anti-rabbit, anti-goat and anti-rat IgG, Thermo Fisher Scientific) were diluted 1:500 in blocking solution and incubated at room temperature for 1 h. Cells were washed once with PBS and incubated for 10 min with DAPI (0.5 mg ml^−1^) in PBS at room temperature. After three washing steps in PBS, the coverslips were briefly washed with distilled water and mounted onto 6 µl Mowiol-based mounting medium (Mowiol 4.88 (Calbiochem) in glycerol/Tris), whereas the wells of the 96-well plates were kept filled with PBS.

### Immunoblotting

Proteins were separated by standard SDS–PAGE and transferred onto PVDF membranes. Membranes were blocked with 5% milk in PBS-T (PBS + 0.1% Tween-20) for 1 h at room temperature and incubated with primary antibodies over night at 4 °C. The membranes were then washed three times with PBS-T and incubated with HRP-conjugated secondary antibodies for 1 h at room temperature, washed again three times with PBS-T and protein signals were detected using ECL Western Blotting Detection Reagent (Thermo Fisher Scientific) and an OPTIMAX X-Ray Film Processor (PROTEC Medizintechnik). To enable detection of multiple target proteins on the same membrane without stripping (typically 2–3 target proteins per membrane with sufficiently distinct molecular weight), membranes were cut horizontally, each piece was probed with specific primary and secondary antibodies, and the membrane was then reassembled for ECL detection. Original western blot scans are provided in Supplementary Fig. 1.

### Clonogenic survival assay

Cells transfected with the indicated siRNAs were seeded at single-cell density and exposed to the indicated drugs at the indicated final concentrations. All conditions were performed in triplicates. Cells were then incubated for 10 days and the number of colonies with more than 50 cells was counted after staining with crystal violet (0.5% crystal violet (Sigma-Aldrich) in 20% ethanol). GraphPad Prism (v.9 and 10) was used to display clonogenic survival data.

### scRNA-seq and SORT-seq

For SORT-seq, cells were sorted on a FACS Aria III 5L (FACS Diva Software v.8.0.1) system equipped with a 488 nm laser line. For the polyploidy dataset, pevonedistat-treated cells were treated with 175 nM of pevonedistat for 24 h. Before single-cell sorting, all live cells were incubated for 1 h with a final concentration of 5 µg ml^−1^ Hoechst 33342 (Thermo Fisher Scientific). All reagents used until the moment of sorting also contained 5 µg ml^−1^ Hoechst 33342 (Thermo Fisher Scientific). For the scSeq dataset, where IR-treated cells were compared to untreated cells, U-2 OS cells were treated with 4 Gy of IR 48 h before sorting or left untreated. Cells were sorted into 384-well plates that were acquired from Single Cell Discoveries (Bio-Rad, HSP3801), each well containing 10 µl sterile mineral oil (Sigma-Aldrich, M5310) and 50 nl DNA oligo primer (Sigma-Aldrich, M8410). Cells with increased ploidy were gated according to their Hoechst signal and, as a reference for the whole population, the gate was adjusted in the same sample. After sorting, plates were immediately centrifuged and placed on dry ice. They were stored at -80 °C and were shipped on dry ice to Single Cell Discoveries, where scRNA-seq was performed according to an adapted version of the SORT-seq protocol^65^ with primers described previously^66^. Cells were heat-lysed at 65 °C followed by cDNA synthesis. After second-strand cDNA synthesis, all of the barcoded material from one plate was pooled into one library and amplified using in vitro transcription (IVT). After amplification, library preparation was performed according to the CEL-Seq2 protocol^67^ to prepare a cDNA library for sequencing using TruSeq small RNA primers (Illumina). The DNA library was paired-end sequenced on the Illumina NextSeq 500 system, high output, with the 1 × 75 bp Illumina kit (read 1: 26 cycles, index read: 6 cycles, read 2: 60 cycles).

During sequencing, read 1 was assigned 26 bp and was used to identify the Illumina library barcode, cell barcode and UMI. Read 2 was assigned 60 bp and was used to map to the reference genome *Homo sapiens* GRCh38.p13 with STARSolo (v.2.7.10b)^68^. In brief, mapping and generation of count tables were automated using the STARSolo v.2.7.10b and aligner. For mapping, no UMI cut-off was used and intronic reads were not included. Multi-gene reads were not counted. Unsupervised clustering and differential gene expression analysis was performed with the Seurat R toolkit^69^. For this, logarithmic normalization was applied, which normalizes to the total RNA counts in each cell. This approach consists of dividing each raw UMI count by the total detected RNAs in that cell, multiplying by a scale factor (10,000), adding a pseudocount (1) and performing log transformation. For visualization of the *t*-SNE, the clustering algorithm used was the original Louvain algorithm where the first 19 principal components were used with a *k* parameter of 30 and a resolution of 0.5.

Subclustering of the polyploid samples was performed with Louvain resolution of 0.5 for both samples with 50 principal components as identified by principal component analysis. Differential gene expression analysis for all comparisons was done using the Venice method, a nonparametric statistical test for single-cell data^70^ in BioTuring BBrowser X software (BioTuring). Tables were extracted and plotted in TIBCO Spotfire (v.7.9.1, v.10.10.1). For the AUCellScores the integrated tool in BioTuring BBrowser X was used. For GO-analysis, ShinyGO (v.0.77 and v.0.80) available online (https://bioinformatics.sdstate.edu/go/)^71^ was used. For generation of Venn diagrams, the tool InteractiVenn (https://www.interactivenn.net)^72^ was used.

The data analysis for the replicate samples was done as described above, except that a different version of the mapper STARSolo (v.2.7.11b)^68^ was used. For subclustering of the polyploid cells of the replicate sample, a Louvain resolution of 0.5 was used for the HRAS polyploid sample and a Louvain resolution of 1.0 was used for the Pevo polyploid sample. Sequencing raw data and processed Poisson-corrected data files for both replicates of the polyploidy data set are accessible on GEO (GSE255874).

Cell clustering analysis of the IR-treated samples was performed with the Seurat R Bioconductor package^69^, using the SC transformed counts generated using the ‘v2’ vst flavour, with Louvain resolution of 0.6 and the first 20 principal components as identified by principal component analysis, for both samples. Dimension reduction was performed using the UMAP method, using the first 20 principal components. Differential expression was performed between treated versus untreated cells using the FindMarkers() function, using the option to return only positive genes. Enriched pathways per cluster were generated using the enrichGO function of the clusterProfiler Bioconductor R package^73^ or the Enrichr gene list enrichment analysis tool^74^, using the marker genes identified per cluster from the FindMarkers() function. All R functions were executed on R v.4.4.2 (https://www.R-project.org) and Bioconductor v.3.20. All scRNA-seq and SORT-seq analyses were performed on two independent sets of samples. Sequencing raw data and processed data files for the IR-treated versus UT dataset are accessible on GEO (GSE288487).

### Bulk RNA-seq

Cells were treated for 24 h with 4 Gy of IR or left untreated. RNA was extracted from triplicate samples using the TRIzol RNA MiniPrep Plus Kit according to the manufacturer’s protocol. Extracted RNA was prepared for sequencing by the Functional Genomics Center Zurich (FGCZ) using the Illumina TruSeq Total RNA Library Prep assay according to the manufacturer’s protocol. Sequencing was performed on the Illumina NovaSeq 6000 system using the S1 Reagent Kit v1.5 (100 cycles) according to the manufacturer’s protocol. Demultiplexing was performed using the Illumina bcl2fastq Conversion Software (v.2.20.0.422). Individual library sizes ranged from 22 million to 52 million reads.

RNA-seq analysis was performed using the SUSHI framework^75^, which encompassed the following steps: read quality was inspected using FastQC, and sequencing adaptors were removed using fastp^76^; pseudoalignment and transcriptomic counts of the RNA-seq reads was performed using the Kallisto Bioconductor R package^77^ with the GENCODE human genome build GRCh38.p13 (release 37)^78^; differential expression using the generalized linear model as implemented by the edgeR Bioconductor R package^79^; and Gene Ontology (GO) term pathway analysis using the hypergeometric over-representation test with the enrichGO function of the clusterProfiler Bioconductor R package^73^ or the Enrichr gene list enrichment analysis tool^74^. Additional figures were generated using the exploreDE Shiny app (10.5281/zenodo.13927692). All R functions were executed on R v.4.4.2 (https://www.R-project.org) and Bioconductor v.3.20. Bulk sequencing data files are accessible on GEO (GSE288485).

### Primary antibodies used in this study

H2AX phospho-S139 (BioLegend, 613401, 1:1,000 for IF), pRb (Cell Signaling Technologies, 8516S, 1:500 for IF), p21 (Abcam, ab109520, 1:500 for IF and WB), p53 (Thermo Fisher Scientific, AHO0152, 1:500 for IF and WB), 53BP1 (Novus Biologicals, NB100-304, 1:1,000 for IF), cyclin A (Abcam, ab181591, 1:500 for IF), cyclin E (Abcam, ab208696, 1:1,000 for WB), HRAS (GeneTex, GTX116041, 1:500 for WB), PCNA (Santa Cruz, sc-56, 1:2,000 for WB), tubulin (Sigma-Aldrich, T6199, 1:5,000 for WB), AMBRA1 (Santa Cruz, sc-398204, 1:500 for WB), KAP1 phospho-S824 (Abcam, ab70369, 1:500 for WB), KAP1 (Bethyl, A300-274A, 1:2,000 for WB), CHK1 phospho-S296 (Abcam, ab79758, 1:500 for WB), CHK1 (Abcam, ab40866, 1:500 for WB), RPA32 phospho-S4/8 (Bethyl, A300-245A, 1:1,000 for WB), RPA32/RPA2 (Abcam, ab2175, 1:500 for WB), BrdU (Abcam, ab6326, 1:250 for IF; BD Biosciences, 347580, 1:80 for IF), MCM2 (Santa Cruz, sc-9839, 1:100 for IF) and MCM7 (Santa Cruz, sc-9966, 1:100 for IF).

### Secondary antibodies used in this study

Alexa Fluor 647 goat anti-rabbit (Thermo Fisher Scientific, A21244, 1:500 for IF), Alexa Fluor 647 goat anti-mouse (Thermo Fisher Scientific, A21235, 1:500 for IF), Alexa Fluor 568 goat anti-rabbit (Thermo Fisher Scientific, A11036, 1:500 for IF), Alexa Fluor 568 goat anti-mouse (Thermo Fisher Scientific, A11031, 1:500 for IF), Alexa Fluor 555 goat anti-rat (Thermo Fisher Scientific, A21434, 1:250 for IF), Alexa Fluor 488 goat anti-rabbit (Thermo Fisher Scientific, A11034, 1:500 for IF), Alexa Fluor 488 goat anti-mouse (Thermo Fisher Scientific, A11029, 1:500 for IF), Alexa Fluor 488 rabbit anti-goat (Thermo Fisher Scientific, A11078, 1:500 for IF), goat anti-rabbit IgG antibody (H+L), peroxidase (Vector Laboratories, PI-1000-1, 1:10,000 for WB), goat anti-mouse IgG Antibody (H+L), peroxidase (Vector Laboratories, PI-2000-1, 1:10,000 for WB).

### Statistics and reproducibility

Apart from time-lapse microscopy, no samples were measured repeatedly, and all other measurements were taken from distinct samples. Statistical analysis was performed in GraphPad Prism (v.9 and 10). Two-tailed unpaired *t*-tests, *χ*^2^ tests, Fisher’s exact tests or one-way ANOVA followed by Tukey’s post hoc test were performed as indicated in the figure legends. *P ≤ 0.05, **P ≤ 0.01, ***P ≤ 0.001, ****P ≤ 0.0001. All extended time-lapse experiments were performed at least twice for each experimental condition (that is, independent biological replicates with different batches of cells and the live-cell microscopy performed in different weeks), each experiment with 1–5 wells per experimental condition (typically 2–3 wells per condition) and multiple images taken per well (typically 9 images per well). Bulk RNA-seq was performed in triplicates, scRNA-seq was performed in duplicates.

Control western blots (Supplementary Figs. 3a,b and 6b) were performed once, control DNA fibre experiments (Extended Data Figs. 9c and 10c) were performed twice. All of the other experiments were performed at least three times, and the presented results were reliably reproduced.

Sample numbers, numbers of experiments performed, statistical tests, exact *P* values, and definition of error bars and box plots are as follows: Fig. 1c, 3 independent experiments performed, *n* = 2,955 (parental) and *n* = 3,018 (edited) cells are shown. Fig. 1d, 3 independent experiments performed, representative images are shown. Fig. 1e, 3 independent experiments performed, representative images are shown. Fig. 1i, 3 independent experiments performed, *n* = 24 cell lineages shown. Fig. 1j, 3 independent experiments performed, *n* = 24 lineages shown. Fig. 2a, 3 independent live-cell experiments performed, *n =* 51 cells analysed; the box plot limits indicate the 25th percentile (Q1) and 75th percentile (Q3); the boxes represent the IQR with the median value (solid lines); the whiskers define the lower and upper adjacent value; dots show outliers smaller than Q1 − 1.5 × IQR and greater than Q3 + 1.5 × IQR. Fig. 2c, 3 independent experiments performed, *n =* 51 (UT), *n =* 43 (APH) cells analysed; the box plot limits indicate Q1 and Q3; boxes represent the IQR with the median value (solid lines); the whiskers define the lower and upper adjacent value; dots show outliers smaller than Q1 − 1.5 × IQR and greater than Q3 + 1.5 × IQR. Fig. 2d, 3 independent experiments performed, *n =* 51 (UT), *n =* 38 (ATRi) cells analysed; the box plot limits indicate Q1 and Q3; boxes represent the IQR with the median value (solid lines); the whiskers define the lower and upper adjacent value; dots show outliers smaller than Q1 − 1.5 × IQR and greater than Q3 + 1.5 × IQR. Fig. 3a, *P* values were derived from a differential gene expression test using the generalized linear model as implemented by edgeR Bioconductor R package with Benjamini–Hochberg multiple test correction, *n =* 14,664 unique genes. Fig. 3b, *P* value (two-sided Fisher’s exact test on genes with residuals of >0.5 versus genes with residuals of ≤0.5): 2.45 × 10^–12^, odds ratio = 1.90, confidence interval (CI): 1.58 to 2.299, *n =* 9,797 unique genes. Fig. 3c, *P* values were derived through Enrichr by one-sided right-tailed Fisher’s test with Benjamini–Hochberg multiple-test correction. Fig. 3d, 3 independent experiments performed, *n =* 5,065 (0 Gy), *n =* 3,747 (0.25 Gy), *n =* 3,605 (0.5 Gy), *n =* 3,632 (1 Gy), *n =* 4,068 (2 Gy), *n =* 3,686 (4 Gy), *n =* 3,938 (10 Gy) cells are shown; the solid line indicates the mean and the dashed lines indicate the s.d. Fig. 3e, 3 independent experiments performed. Fig. 3f, 3 independent experiments performed, *n =* 855 (UT), *n =* 1,011 (ATRi), *n =* 1,205 (IR), *n =* 965 (ATRi→IR), *n =* 273 (UT in G1), *n =* 598 (ATRi in G1), *n =* 404 (IR in G1), *n =* 557 (ATRi→IR in G1) cells are shown; *P* values and CIs γH2AX foci (one-way ANOVA followed by Tukey’s post hoc test): *P =* 0.0012 (CI: −3.886 to −0.7074) for UT versus ATRi, *P* < 0.0001 (CI: −5.710 to −2.865) for IR versus ATRi→IR; *P* values and CIs 53BP1 foci (one-way ANOVA followed by Tukey’s post hoc test): *P =* 0.0072 (CI: 0.2131 to 1.908) for IR versus ATRi→IR; the solid line indicates the mean and the dashed lines indicate the s.d. Fig. 4a, 3 independent experiments performed, *n =* 7,347 (control), *n =* 7,310 (pevonedistat 100 nM), *n =* 7,215 (pevonedistat 175 nM), *n =* 7,088 (pevonedistat 250 nM) cells shown. Fig. 4b, 3 independent live-cell experiments performed, *n =* 557 (G1 peak, control), *n =* 170 (≥G2 peak, control), *n =* 221 (G1 peak, pevonedistat), *n =* 282 (≥G2 peak, pevonedistat) cells analysed; the box plot limits indicate Q1 and Q3; boxes represent the IQR with the median value (solid lines); the whiskers define the lower and upper adjacent value; dots show outliers smaller than Q1 − 1.5 × IQR and greater than Q3 + 1.5 × IQR. Fig. 4c, 3 independent live-cell experiments performed, *n =* 557 (G1 peak, control), *n =* 170 (≥G2 peak, control), *n =* 221 (G1 peak, pevonedistat), *n =* 282 (≥G2 peak, pevonedistat) cells analysed; the box plot limits indicate Q1 and Q3; boxes represent the IQR with the median value (solid lines); the whiskers define the lower and upper adjacent value; dots show outliers smaller than Q1 − 1.5 × IQR and greater than Q3 + 1.5 × IQR. Fig. 4d, 3 independent live-cell experiments performed, *n =* 557 (G1 peak, control), *n =* 170 (≥G2 peak, control), *n =* 221 (G1 peak, pevonedistat), *n =* 282 (≥G2 peak, pevonedistat) cells analysed; the box plot limits indicate Q1 and Q3; boxes represent the IQR with the median value (solid lines); the whiskers define the lower and upper adjacent value; dots show outliers smaller than Q1 − 1.5 × IQR and greater than Q3 + 1.5 × IQR. Fig. 4e, 3 independent live-cell experiments performed, representative examples are shown. Fig. 4f, 3 independent live-cell experiments performed, representative images are shown. Fig. 4g, 2 independent live-cell experiments performed, *n =* 61 (normal), *n =* 94 (endoreplication), *n =* 84 (rereplication) cells analysed; where the solid line indicates the mean and the dashed lines indicate the s.d. Fig. 4h, 2 independent live-cell experiments performed, *n =* 61 (normal), *n =* 94 (endoreplication), *n =* 84 (rereplication) cells analysed; the solid line indicates the mean and the dashed lines indicate the s.d., *P* values and CI (two-tailed unpaired *t-*test): *P* < 0.0001 (CI: 423.0 to 673.3). Fig. 4i, 2 independent live-cell experiments performed, *n =* 61 (normal), *n =* 94 (endoreplication), *n =* 84 (rereplication) cells analysed; the solid line indicates the mean and the dashed lines indicate the s.d.; *P* values and CI (two-tailed unpaired *t-*test): *P* < 0.0001 (CI: 186.4 to 372.6). Fig. 4j, 2 independent live-cell experiments performed, *n =* 61 (normal), *n =* 94 (endoreplication), *n =* 84 (rereplication) cells analysed; the solid line indicates the mean and the dashed lines indicate the s.d.; *P* values and CI (two-tailed unpaired *t-*test): *P* < 0.0001 (CI: 738.3 to 2,175). Fig. 4k, 2 independent live-cell experiments performed, *n =* 61 (normal), *n =* 94 (endoreplication), *n =* 84 (rereplication) cells analysed; the solid line indicates the mean and the dashed lines indicate the s.d., *P* values and CI (two-tailed unpaired *t-*test): *P =* 0.0022 (CI: 536.2 to 2,404). Fig. 5a, 2 independent live-cell experiments performed, *n =* 88 (EV), *n =* 88 (HRAS), *n =* 489 (G1 peak, EV), *n =* 191 (≥G2 peak, EV), *n =* 411 (G1 peak, HRAS), *n =* 216 (≥G2 peak, HRAS) cells analysed; the box plot limits indicate Q1 and Q3; boxes represent the IQR with the median value (solid lines); the whiskers define the lower and upper adjacent value; dots show outliers smaller than Q1 − 1.5 × IQR and greater than Q3 + 1.5 × IQR. Fig. 5f, 3 independent experiments performed, *n =* 7,347 (control), *n =* 7,215 (pevonedistat) cells shown. Fig. 5g, 2 independent live-cell experiments performed, *n =* 20 and 15 (pevonedistat), *n =* 4 and 5 (etoposide), *n =* 20 and 20 (RO3306) cells undergoing polyploidization analysed, where solid lines depict the mean and dots represent the cumulative percentage of each experiment.

Extended Data Figures: Extended Data Fig. 1a, 2 independent live-cell experiments performed, representative images are shown. Extended Data Fig. 1c, 1 experiment performed. Extended Data Fig. 1e, 3 independent experiments performed, *n =* 1,066 (parental UT), *n =* 1,103 (edited UT), *n =* 1,133 (parental IR), *n =* 1,088 (edited IR) cells shown. Extended Data Fig. 1f, 3 independent experiments performed, *n =* 1,132 (parental UT), *n =* 1,141 (edited UT), *n =* 1,123 (parental IR), *n =* 1103 (edited IR) cells shown. Extended Data Fig. 1g, 2 independent live-cell experiments performed, *n =* 2,038 (imaged), *n =* 2,036 (not imaged) cells shown. Extended Data Fig. 1h, 2 independent live-cell experiments performed, *n =* 2,038 (imaged), *n =* 2,036 (Not imaged) cells shown. Extended Data Fig. 1i, 2 independent live-cell experiments performed, *n =* 2,051 (imaged), *n =* 2,034 (not imaged) cells shown. Extended Data Fig. 2b, 3 independent experiments performed, *n =* 4,833 (siCtrl UT), *n =* 4,486 (siCtrl IR), *n =* 4,510 (si53BP1 UT), *n =* 4,407 (si53BP1 IR) cells analysed; where the solid line indicates the mean and the dashed lines indicate the s.d.; *P* values and CIs (one-way ANOVA followed by Tukey’s post hoc test): *P* < 0.0001 (CI: −0.4840 to −0.4030) for UT siCtrl versus IR siCtrl, *P* < 0.0001 (CI: 0.5565 to 0.6393) for IR siCtrl versus IR si53BP1. Extended Data Fig. 2d, 3 independent experiments performed, *n =* 4,453 cells shown. Extended Data Fig. 2e, 3 independent experiments performed, *n =* 945 (G1), *n =* 2,963 (S), *n =* 545 (G2/M) cells shown; *P* values and CIs (one-way ANOVA followed by Tukey’s post hoc test): *P* < 0.0001 (CI: −39.23 to −34.93) for G1 versus S, *P* < 0.0001 (CI: 31.86 to 37.23) for S versus G2/M; box plot limits indicate Q1 and Q3; boxes represent the IQR with the median value (solid lines); the whiskers define the lower and upper adjacent value; dots show outliers smaller than Q1 − 1.5 × IQR and greater than Q3 + 1.5 × IQR. Extended Data Fig. 3b, 3 independent live-cell experiments performed, *n =* 23 lineages shown. Extended Data Fig. 3d, 3 independent experiments performed, representative results are shown. Extended Data Fig. 3e, 3 independent live-cell experiments performed, *n =* 26 lineages shown. Extended Data Fig. 3g, 3 independent live-cell experiments performed, *n =* 27 lineages shown. Extended Data Fig. 4b, 3 independent experiments performed, representative images are shown. Extended Data Fig. 4c, 3 independent experiments performed, *n =* 1,659 (control), *n =* 1,324 (replication stress) cells shown. Extended Data Fig. 4d, 3 independent experiments performed, *n =* 1,659 (control), *n =* 1,324 (replication stress) cells shown. Extended Data Fig. 4e, 3 independent experiments performed, *n =* 1,659 (control), *n =* 1,324 (replication stress) cells shown. Extended Data Fig. 4f, 3 independent experiments performed, *n =* 4,835 (untreated), *n =* 2,781 (APH), *n =* 3,217 (ATRi) cells shown. Extended Data Fig. 4g, 3 independent experiments performed, *n =* 3,022 (untreated), *n =* 2,966 (APH), *n =* 3,008 (ATRi) cells shown. Extended Data Fig. 4h, 3 independent experiments performed, *n =* 1,269 (untreated), *n =* 1,183 (APH), *n =* 1,053 (ATRi) cells shown. Extended Data Fig. 4i, 3 independent experiments performed, *n =* 3,165 (untreated), *n =* 2,932 (APH), *n =* 3,054 (ATRi) cells shown. Extended Data Fig. 5a, 3 independent live-cell experiments performed, *n =* 51 (UT), *n =* 43 (APH) cells analysed; the box plot limits indicate Q1 and Q3; boxes represent the IQR with the median value (solid lines); the whiskers define the lower and upper adjacent value; dots show outliers smaller than Q1 − 1.5 × IQR and greater than Q3 + 1.5 × IQR. Extended Data Fig. 5b, 3 independent live-cell experiments performed, *n =* 51 (UT), *n =* 38 (ATRi) cells analysed; the box plot limits indicate Q1 and Q3; boxes represent the IQR with the median value (solid lines); the whiskers define the lower and upper adjacent value; dots show outliers smaller than Q1 − 1.5 × IQR and greater than Q3 + 1.5 × IQR. Extended Data Fig. 5c, 3 independent live-cell experiments performed, *n =* 51 (UT), *n =* 43 (APH) cells analysed; the box plot limits indicate Q1 and Q3; boxes represent the IQR with the median value (solid lines); the whiskers define the lower and upper adjacent value; dots show outliers smaller than Q1 − 1.5 × IQR and greater than Q3 + 1.5 × IQR. Extended Data Fig. 5d, 3 independent live-cell experiments performed, *n =* 51 (UT), *n =* 38 (ATRi) cells analysed; the box plot limits indicate Q1 and Q3; boxes represent the IQR with the median value (solid lines); the whiskers define the lower and upper adjacent value; dots show outliers smaller than Q1 − 1.5 × IQR and greater than Q3 + 1.5 × IQR. Extended Data Fig. 5e, 3 independent live-cell experiments performed, *n =* 29 (53BP1 foci) and *n =* 27 (γH2AX, p53, p21) sister cell pairs for UT, *n =* 49 (53BP1 foci), *n =* 33 (γH2AX), and *n =* 31 (p53, p21) sister cell pairs for APH, *n =* 36 (53BP1 foci) and *n =* 31 (γH2AX, p53, p21) sister cell pairs for ATRi; *P* values (*χ*^2^ test): *P =* 0.0004 for UT versus ATRi (53BP1 foci), *P =* 0.0012 for UT versus ATRi (γH2AX levels), *P =* 0.0284 for UT versus APH (p53 levels), *P =* 0.0031 for UT versus ATRi (p53 levels), *P =* 0.0131 for UT versus APH (p21 levels), *P* < 0.0001 for UT versus ATRi (p21 levels). Extended Data Fig. 6b, 3 independent experiments performed, *n =* 3,592 (siCtrl UT), *n =* 4,824 (siCtrl IR), *n =* 3,267 (si53BP1 UT), *n =* 4,662 (si53BP1 IR) cells shown; *P* values and CIs (one-way ANOVA followed by Tukey’s post hoc test): *P* < 0.0001 for UT siCtrl versus IR siCtrl (CI: −1.431 to −1.288), *P* < 0.0001 for IR siCtrl versus IR si53BP1 (CI: 1.413 to 1.547); the solid line indicates the mean and the dashed lines indicate the s.d. Extended Data Fig. 6d, 3 independent experiments performed, *n =* 4,083 cells shown. Extended Data Fig. 6e, 3 independent experiments performed, *n =* 1,972 (G1), *n =* 1,759 (S), *n =* 352 (G2) cells shown; *P* values and CIs (one-way ANOVA followed by Tukey’s post hoc test): *P* < 0.0001 for G1 versus S (CI: −38.77 to −36.22), *P* < 0.0001 for S versus G2 (CI: 31.77 to 36.31); box plot limits indicate Q1 and Q3; boxes represent the IQR with the median value (solid lines); the whiskers define the lower and upper adjacent value; dots show outliers smaller than Q1 − 1.5 × IQR and greater than Q3 + 1.5 × IQR. Extended Data Fig. 6f, 3 independent experiments performed, *n =* 2,519 (untreated), *n =* 2,508 (APH), *n =* 2,501 (ATRi) cells shown. Extended Data Fig. 6g, 3 independent experiments performed, *n =* 2,519 (untreated), *n =* 2,508 (APH), *n =* 2,501 (ATRi) cells shown. Extended Data Fig. 6h, 3 independent experiments performed, *n =* 2,267 (untreated), *n =* 2,134 (APH), *n =* 2,143 (ATRi) cells shown. Extended Data Fig. 6i, 3 independent experiments, *n =* 2,267 (untreated), *n =* 2,134 (APH), *n =* 2,143 (ATRi). Extended Data Fig. 6j, 3 independent experiments performed, representative results are shown. Extended Data Fig. 7b, *P* values were derived through Enrichr using a one-sided right-tailed Fisher’s test with Benjamini–Hochberg multiple-test correction. Extended Data Fig. 7c, *P* values were derived from differential gene expression test using the generalized linear model as implemented by the edgeR Bioconductor R package with Benjamini–Hochberg multiple-test correction, boxes represent the IQR with the median value (solid lines); the whiskers define the lower and upper adjacent value; dots show outliers smaller than Q1 − 1.5 × IQR and greater than Q3 + 1.5 × IQR. *n =* 3 independent samples. Extended Data Fig. 7d, boxes represent the IQR with the median value (solid lines), the whiskers define the lower and upper adjacent value, and the violin plot represents the total distribution. *n =* 346 (UT), *n =* 227 (IR) cells analysed. Extended Data Fig. 7e, boxes represent the IQR with the median value (solid lines), the whiskers define the lower and upper adjacent value, and the violin plot represents the total distribution. *n =* 346 (UT), *n =* 227 (IR) cells for experiment 1 and *n =* 355 (UT), *n =* 230 (IR) cells for experiment 2. Extended Data Fig. 7f, *n =* 573 cells analysed. Extended Data Fig. 7g, *n =* 346 (UT), *n =* 227 (IR) cells analysed. Extended Data Fig. 7h, *P* values were derived by one-sided right-tailed Fisher’s test with Benjamini–Hochberg multiple-test correction through clusterProfiler. Extended Data Fig. 7i, Pearson’s correlation coefficient, *n =* 9,279 all genes, *n =* 418 DDR genes. Extended Data Fig. 8a, 3 independent live-cell experiments performed. Extended Data Fig. 8b, 3 independent live-cell experiments performed. Extended Data Fig. 8c, 3 independent experiments performed, *n =* 423 (control), *n =* 158 (IR) cells shown; *P* values and CI (two-tailed unpaired *t-*test): *P* < 0.0001 (CI: 3.246 to 3.978); the solid line indicates the mean and the dashed lines indicate the s.d. Extended Data Fig. 8d, 3 independent experiments performed, *n =* 4,049 (UT), *n =* 4,079 (4-OHT), *n =* 4,377 (4-OHT-IAA) cells shown; *P* values and CIs (one-way ANOVA followed by Tukey’s post hoc test): *P* < 0.0001 for UT versus 4-OHT (CI: −2.414 to −2.093), *P* < 0.0001 for 4-OHT versus 4-OHT-IAA (CI: 1.669 to 1.984); the solid line indicates the mean and the dashed lines indicate the s.d. Extended Data Fig. 8e, 3 independent experiments performed, *n =* 4,049 (UT), *n =* 4,079 (4-OHT), *n =* 4,377 (4-OHT-IAA) cells shown; *P* values and CIs (one-way ANOVA followed by Tukey’s post hoc test): *P* < 0.0001 for UT versus 4-OHT (CI: −6.751 to −5.977), *P* < 0.0001 for 4-OHT versus 4-OHT-IAA (CI: 2.990 to 3.749); the solid line indicates the mean and the dashed lines indicate the s.d. Extended Data Fig. 8f, 2 independent live-cell experiments performed. Extended Data Fig. 8g, 3 independent experiments performed, *n =* 621 (−4-OHT), *n =* 639 (+4-OHT + IAA) cells shown; *P* values and CI (two-tailed unpaired *t-*test): *P =* 0.0003 (CI: 0.1963 to 0.6682); the solid line indicates the mean and the dashed lines indicate the s.d. Extended Data Fig. 8h, 3 independent experiments performed, *n =* 2,428 (WT control), *n =* 614 (WT IR), *n =* 2,406 (p53KO control), *n =* 2,106 (p53-KO IR) cells shown; *P* values and CIs (one-way ANOVA followed by Tukey’s post hoc test): *P* < 0.0001 for WT control versus WT IR (CI: −0.5428 to −0.2159), *P* < 0.0001 for p53KO control versus p53KO IR (CI: −0.9969 to −0.7810); the solid line indicates the mean and the dashed lines indicate the s.d. Extended Data Fig. 8i, 2 independent live-cell experiments performed, *n =* 32 (53BP1 foci) and *n =* 52 (γH2AX, p53, pRb) sister cell pairs for UT; *n =* 54 (53BP1 foci) and *n =* 68 (γH2AX, p53, pRb) sister cell pairs for 4 Gy; *P* values (*χ*^2^ test): *P =* 0.0088 for UT versus 4 Gy (53BP1 foci), *P =* 0.0080 for UT versus 4 Gy (γH2AX levels), *P =* 0.0397 for UT versus 4 Gy (p53 levels), *P =* 0.0176 for UT versus 4 Gy (pRb levels). Extended Data Fig. 8j, 2 independent live-cell experiments performed. Extended Data Fig. 9a, 3 independent experiments performed, *n =* 45,070 (control), *n =* 16,136 (pevonedistat 100 nM), *n =* 10,579 (pevonedistat 175 nM), *n =* 7,551 (pevonedistat 250 nM) cells shown. Extended Data Fig. 9b, 3 independent experiments performed, representative images shown. Extended Data Fig. 9c, 2 independent experiments performed, DNA fibre length from *n =* 100 (control), *n =* 100 (pevonedistat) fibres shown; *P* values and CI (two-tailed unpaired *t-*test): *P* < 0.0001 (CI: −11.40 to −9.096); the red solid line indicates the median. Extended Data Fig. 9d, 3 independent experiments performed, *n =* 2,142 (control), *n =* 2,120 (pevonedistat) cells shown. Extended Data Fig. 9e, 3 independent experiments performed, *n =* 1,368 (control), *n =* 1,340 (pevonedistat) cells shown. Extended Data Fig. 9f, 3 independent live-cell experiments performed, representative examples are shown. Extended Data Fig. 9g, 3 independent live-cell experiments performed, representative examples are shown. Extended Data Fig. 9h, 3 independent live-cell experiments performed, *n =* 9, *n =* 13, *n =* 15 (G1), *n =* 8, *n =* 12, *n =* 15 (S) cells undergoing polyploidization; data are mean ± s.d. Extended Data Fig. 9i, 2 independent live-cell experiments performed, representative examples are shown. Extended Data Fig. 9j, 2 independent live-cell experiments performed, representative examples are shown. Extended Data Fig. 9k, 2 independent experiments performed, *n =* 2,221 (siCtrl), *n =* 2,197 (siGMNN) cells shown. Extended Data Fig. 9l, 2 independent live-cell experiments performed, *n =* 43 (normal), *n =* 15 (endoreplication), *n =* 37 (rereplication) cells analysed; the solid line indicates the mean and the dashed lines indicate the s.d. Extended Data Fig. 9m, 2 independent live-cell experiments performed, *n =* 43 (normal), *n =* 15 (endoreplication), *n =* 37 (rereplication) cells analysed; *P* values and CI (two-tailed unpaired *t-*test): *P =* 0.0256 (CI: 6.720 to 98.88) for endoreplication versus rereplication; the solid line indicates the mean and the dashed lines indicate the s.d. Extended Data Fig. 9n, 2 independent live-cell experiments performed, *n =* 43 (normal), *n =* 15 (endoreplication), *n =* 37 (rereplication) cells analysed; *P* values and CI (two-tailed unpaired *t-*test): *P =* 0.0214 (CI: 4.769 to 56.93) for endoreplication versus rereplication; the solid line indicates the mean and the dashed lines indicate the s.d. Extended Data Fig. 9o, 2 independent live-cell experiments performed, *n =* 43 (normal), *n =* 15 (endoreplication), *n =* 37 (rereplication) cells analysed; the solid line indicates the mean and the dashed lines indicate the s.d. Extended Data Fig. 9p, 2 independent live-cell experiments performed, *n =* 43 (normal), *n =* 15 (endoreplication), *n =* 37 (rereplication) cells analysed; the solid line indicates the mean and the dashed lines indicate the s.d. Extended Data Fig. 10a, 3 independent experiments performed, representative results are shown. Extended Data Fig. 10b, 3 independent experiments performed, representative results are shown. Extended Data Fig. 10c, 2 independent experiments performed, DNA fibre length of *n =* 120 (EV), *n =* 120 (HRAS), *n =* 120 (cyclin E) fibres shown; *P* values and CIs (one-way ANOVA followed by Tukey’s post hoc test): *P* < 0.0001 (CI: 1.699 to 5.797) for EV versus HRAS, *P =* 0.0003 (CI: 1.399 to 5.497) for EV versus cyclin E; the red solid line indicates the median. Extended Data Fig. 10d, 4 independent experiments performed, *n =* 100 cells per replicate (EV), *n =* 100 cells per replicate (HRAS), *n =* 100 cells per replicate (cyclin E); *P* values and CIs (one-way ANOVA followed by Tukey’s post hoc test): *P =* 0.0234 (CI: −25.86 to −3.645) for EV versus HRAS, *P =* 0.0078 (CI: −17.75 to −5.749) for EV versus cyclin E; data are mean ± s.d. Extended Data Fig. 10e, 2 independent live-cell experiments performed, *n =* 88 (EV), *n =* 489 (G1 peak, EV), *n =* 191 (≥G2 peak, EV) cells analysed; the box plot limits indicate Q1 and Q3; boxes represent the IQR with the median value (solid lines); the whiskers define the lower and upper adjacent value; dots show outliers smaller than Q1 − 1.5 × IQR and greater than Q3 + 1.5 × IQR. Extended Data Fig. 10f, 2 independent live-cell experiments performed, *n =* 88 (EV), *n =* 88 (HRAS), *n =* 489 (G1 peak, EV), *n =* 191 (≥G2 peak, EV), *n =* 411 (G1 peak, HRAS), *n =* 216 (≥G2 peak, HRAS) cells analysed; box plot limits indicate Q1 and Q3; boxes represent the IQR with the median value (solid lines); the whiskers define the lower and upper adjacent value; dots show outliers smaller than Q1 − 1.5 × IQR and greater than Q3 + 1.5 × IQR. Extended Data Fig. 10g, 2 independent live-cell experiments performed, *n =* 88 (EV), *n =* 92 (cyclin E), *n =* 489 (G1 peak, EV), *n =* 191 (≥G2 peak, EV), *n =* 548 (G1 peak, cyclin E), *n =* 248 (≥G2 peak, cyclin E) cells analysed; the box plot limits indicate Q1 and Q3; boxes represent the IQR with the median value (solid lines); the whiskers define the lower and upper adjacent value; dots show outliers smaller than Q1 − 1.5 × IQR and greater than Q3 + 1.5 × IQR. Extended Data Fig. 10h, 2 independent live-cell experiments performed, *n =* 88 (EV), *n =* 92 (cyclin E), *n =* 489 (G1 peak, EV), *n =* 191 (≥G2 peak, EV), *n =* 548 (G1, cyclin E), *n =* 248 (≥G2 peak, cyclin E) cells analysed; box plot limits indicate Q1 and Q3; boxes represent the IQR with the median value (solid lines); the whiskers define the lower and upper adjacent value; dots show outliers smaller than Q1 − 1.5 × IQR and greater than Q3 + 1.5 × IQR. Extended Data Fig. 11a, 3 independent experiments performed, *n =* 3,097 (empty vector UT), *n =* 3,026 (HRAS UT), *n =* 3,144 (cyclin E UT), 3,082 (empty vector IR), *n =* 3,149 (HRAS IR), *n =* 3,121 (cyclin E IR) cells shown. Extended Data Fig. 11b, 3 independent experiments performed, representative images are shown. Extended Data Fig. 11c, 3 independent experiments with multiple replicate samples performed, *n =* 3,093, *n =* 3,076, *n =* 3,045, *n =* 3,019, *n =* 3,020, *n =* 3,031 (empty vector UT), *n =* 3,026, *n =* 3,073, *n =* 3,126, *n =* 3,064, *n =* 2,994, *n =* 3,043 (HRAS UT), *n =* 3,144, *n =* 3,012, *n =* 3,003, *n =* 3,013, *n =* 2,991, *n =* 3,090 (cyclin E UT), *n =* 3,082, *n =* 3,044, *n =* 3,014, *n =* 3,003, *n =* 3,068, *n =* 2,985 (empty vector IR), *n =* 3,149, *n =* 3,091, *n =* 3,051, *n =* 3,035, *n =* 3,075, *n =* 2,907 (HRAS IR), *n =* 3,121, *n =* 2,869, *n =* 3,036, *n =* 3,083, *n =* 2,467, *n =* 2,179 (cyclin E IR) cells analysed; *P* values and CIs (one-way ANOVA followed by Tukey’s post hoc test): *P* < 0.0001 (CI: −2.608 to −0.9585) for empty vector UT versus HRAS UT, *P* < 0.0001 (CI: −2.291 to −0.6419) for empty vector UT versus cyclin E UT, *P* < 0.0001 (CI: −2.575 to −0.9252) for empty vector UT versus empty vector IR, *P* < 0.0001 (CI: −4.891 to −3.242) for empty vector UT versus HRAS IR, *P* < 0.0001 (CI: −3.908 to −2.259) for empty vector UT versus cyclin E IR; data are mean ± s.d. Extended Data Fig. 11d, 3 independent experiments performed, *n =* 15,249 (empty vector UT), *n =* 15,262 (HRAS UT), *n =* 15,144 (cyclin E UT), *n =* 15,200 (empty vector IR), *n =* 15,378 (HRAS IR), *n =* 14,548 (cyclin E IR) cells shown. Extended Data Fig. 11f, 3 independent live-cell experiments performed, representative examples are shown. Extended Data Fig. 11g, 2 independent experiments performed, representative results are shown. Extended Data Fig. 11h, 3 independent experiments performed, *n =* 4,867 (−Dox), *n =* 4,995 (+Dox) cells shown. Extended Data Fig. 12a, 2 independent live-cell experiments performed, *n =* 12 (−Dox), *n =* 414 (G1 peak, −Dox), *n =* 271 (≥G2 peak, −Dox) cells analysed; the box plot limits indicate Q1 and Q3; boxes represent the IQR with the median value (solid lines); the whiskers define the lower and upper adjacent value; dots show outliers smaller than Q1 − 1.5 × IQR and greater than Q3 + 1.5 × IQR. Extended Data Fig. 12b, 2 independent live-cell experiments performed, *n =* 12 (−Dox), *n =* 36 (+Dox), *n =* 414 (G1 peak, −Dox), *n =* 271 (≥G2 peak, −Dox), *n =* 286 (G1 peak, +Dox), *n =* 259 (≥G2 peak, +Dox) cells analysed; the box plot limits indicate Q1 and Q3; boxes represent the IQR with the median value (solid lines); the whiskers define the lower and upper adjacent value; dots show outliers smaller than Q1 − 1.5 × IQR and greater than Q3 + 1.5 × IQR. Extended Data Fig. 12c, 2 independent live-cell experiments performed, *n =* 174 (experiment 1), *n =* 231 (experiment 2), mean (solid lines) and individual percentage values are depicted.

Supplementary Figures: Supplementary Fig. 2a, 3 independent experiments performed, *n =* 10,096 (unchallenged), *n =* 3,319 (APH), *n =* 3,388 (ATRi) cells shown. Supplementary Fig. 2b, 3 independent experiments performed, *n =* 10,096 (unchallenged), *n =* 3,319 (APH), *n =* 3,388 (ATRi) cells shown. Supplementary Fig. 2c, 3 independent experiments performed, *n =* 1,021 (G1 unchallenged), *n =* 1,764 (S unchallenged), *n =* 604 (G2/M unchallenged), *n =* 510 (G1 APH), *n =* 2,268 (S APH), *n =* 541 (G2/M APH), *n =* 1,770 (G1 ATRi), *n =* 899 (S ATRi), *n =* 719 (G2/M ATRi) cells shown. Supplementary Fig. 2d, 3 independent experiments performed, representative images are shown. Supplementary Fig. 2e, 3 independent live-cell experiments performed, *n =* 10 lineages per condition; *P* values and CIs for perturbed replication (one-way ANOVA followed by Tukey’s post hoc test): *P* < 0.0001 (CI: −2.148 to −0.6516) for UT versus APH, *P* < 0.0001 (CI: −2.648 to −1.152) for UT versus ATRi, *P* < 0.0001 (CI: −2.148 to −0.6516) for UT versus IR; *P* values and CIs for >10 53BP1 foci (one-way ANOVA followed by Tukey’s post hoc test): *P =* 0.0003 (CI: −1.582 to −0.4182) for UT versus IR; *P* values and CIs for number of divisions (one-way ANOVA followed by Tukey’s post hoc test): *P =* 0.0002 (CI: 0.6531 to 2.347) for UT versus APH, *P* < 0.0001 (CI: 0.7531 to 2.447) for UT versus ATRi, *P =* 0.0011 (CI: 0.4531 to 2.147) for UT versus IR. Supplementary Fig. 3a, 1 experiment performed. Supplementary Fig. 3b, 1 experiment performed. Supplementary Fig. 3c, 2 independent live-cell experiments performed; an example cell lineage is shown. Supplementary Fig. 3d, 2 independent live-cell experiments performed; an example cell lineage is shown. Supplementary Fig. 3e, 2 independent live-cell experiments performed; an example cell lineage is shown. Supplementary Fig. 3f, 2 independent live-cell experiments performed; an example cell lineage is shown. Supplementary Fig. 3g, 2 independent live-cell experiments performed; an example cell lineage is shown. Supplementary Fig. 3h, 2 independent live-cell experiments performed; an example cell lineage is shown. Supplementary Fig. 3i, 2 independent live-cell experiments performed; an example cell lineage is shown. Supplementary Fig. 3j, 2 independent live-cell experiments performed; an example cell lineage is shown. Supplementary Fig. 3k, 2 independent live-cell experiments performed; an example cell lineage is shown. Supplementary Fig. 4a, 2 independent live-cell experiments performed, *n =* 21 lineages shown. Supplementary Fig. 4b, 2 independent live-cell experiments performed, *n =* 17 lineages shown. Supplementary Fig. 4c, 2 independent live-cell experiments performed, *n =* 20 lineages shown. Supplementary Fig. 4d, 2 independent live-cell experiments performed, *n =* 20 lineages shown. Supplementary Fig. 4e, 2 independent live-cell experiments performed, *n =* 20 lineages shown. Supplementary Fig. 4f, 2 independent live-cell experiments performed, *n =* 20 lineages shown. Supplementary Fig. 4g, 2 independent live-cell experiments performed, *n =* 20 lineages shown. Supplementary Fig. 4h, 2 independent live-cell experiments performed, *n =* 20 lineages shown. Supplementary Fig. 4i, 2 independent live-cell experiments performed, *n =* 20 lineages shown. Supplementary Fig. 5a, 2 independent live-cell experiments performed, *n =* 27 cells analysed; the box plot limits indicate Q1 and Q3; boxes represent the IQR with the median value (solid lines); the whiskers define the lower and upper adjacent value; dots show outliers smaller than Q1 − 1.5 × IQR and greater than Q3 + 1.5 × IQR. Supplementary Fig. 5b, 2 independent live-cell experiments performed, *n =* 27 cells (siCtrl), *n =* 47 cells (siAMBRA1) analysed; the box plot limits indicate Q1 and Q3; boxes represent the IQR with the median value (solid lines); the whiskers define the lower and upper adjacent value; dots show outliers smaller than Q1 − 1.5 × IQR and greater than Q3 + 1.5 × IQR. Supplementary Fig. 5c, 2 independent live-cell experiments performed, *n =* 27 cells (siCtrl), *n =* 47 cells (siAMBRA1), *n =* 38 cells (siAMBRA1 + APH) analysed; the box plot limits indicate Q1 and Q3; boxes represent the IQR with the median value (solid lines); the whiskers define the lower and upper adjacent value; dots show outliers smaller than Q1 − 1.5 × IQR and greater than Q3 + 1.5 × IQR. Supplementary Fig. 5d, 2 independent live-cell experiments performed, *n =* 27 cells (siCtrl), *n =* 47 cells (siAMBRA1), *n =* 25 cells (siAMBRA1 + ATRi) analysed; the box plot limits indicate Q1 and Q3; boxes represent the IQR with the median value (solid lines); the whiskers define the lower and upper adjacent value; dots show outliers smaller than Q1 − 1.5 × IQR and greater than Q3 + 1.5 × IQR. Supplementary Fig. 6a, 3 independent experiments performed; *P* values and CI (two-tailed unpaired *t-*test): *P =* 0.0006 (CI: 17.60 to 31.07) for 0.1 μM ATRi, *P =* 0.0114 (CI: 1.740 to 7.593) for 0.2 μM ATRi, *P =* 0.0111 (CI: 1.264 to 5.403) for 0.3 μM ATRi; data are mean ± s.d. Supplementary Fig. 6b, 1 experiment performed. Supplementary Fig. 7b, 2 independent live-cell experiments performed, an example lineage is shown. Supplementary Fig. 7c, 2 independent live-cell experiments performed, an example lineage is shown. Supplementary Fig. 7d, 2 independent live-cell experiments performed, an example lineage is shown. Supplementary Fig. 7e, 2 independent live-cell experiments performed, an example lineage is shown. Supplementary Fig. 8a, 3 independent experiments performed, *n =* 1,482 (control), *n =* 1,511 (IR) cells shown. Supplementary Fig. 8b, 2 independent live-cell experiments performed, *n =* 60 sister cell pairs for UT, *n =* 60 sister cell pairs for IR; *P* value (Fisher’s exact test): *P =* 0.0013. Supplementary Fig. 12i, 3 independent experiments performed, *n =* 2,240 (control), *n =* 1,927 (etoposide), *n =* 1,993 (RO3306) cells shown. Supplementary Fig. 12j, 3 independent experiments performed, *n =* 4,356 (control), *n =* 3,087 (etoposide), *n =* 3,955 (RO3306) cells shown. Supplementary Fig. 12k, 3 independent experiments performed, *n =* 4,356 (control), *n =* 3,087 (etoposide), *n =* 3,955 (RO3306) cells shown. Supplementary Fig. 12l, 3 independent experiments performed, *n =* 1,503 (G1/early S control), *n =* 1156 (mid-S control), *n =* 637 (late S/G2 control), *n =* 516 (G1/early S etoposide), *n =* 872 (mid-S etoposide), *n =* 1,801 (late S/G2 etoposide), *n =* 981 (G1/early S RO3306), *n =* 630 (mid-S RO3306), *n =* 1,249 (late S/G2 RO3306) cells shown.

### Reporting summary

Further information on research design is available in the Nature Portfolio Reporting Summary linked to this article.

## Online content

Any methods, additional references, Nature Portfolio reporting summaries, source data, extended data, supplementary information, acknowledgements, peer review information; details of author contributions and competing interests; and statements of data and code availability are available at 10.1038/s41586-025-08986-0.

## Supplementary information

Supplementary Information A combined Supplementary Information PDF of 14 pages, containing Supplementary Figs. 1–12 and the corresponding legends.

Reporting Summary

Supplementary Table 1 Differential gene expression from scRNA-seq of HRAS polyploid vs. EV cells, related to Supplementary Fig. 9b.

Supplementary Table 2 Differential gene expression from scRNA-seq of Pevonedistat-treated polyploid vs. EV cells, related to Supplementary Fig. 9c.

Supplementary Table 3 Differential gene expression from scRNA-seq of the polyploidy set sub-cluster analysis, related to Supplementary Fig. 12a.

Supplementary Table 4 Differential gene expression from scRNA-seq of the polyploidy set sub-cluster analysis with more stringent cutoffs than in Supplementary Table 3, related to Supplementary Fig. 12a.

Supplementary Table 5 Primer sequences used in this study.

Supplementary Video 1 Cell tracking: live-cell imaging of U-2 OS cells expressing endogenous PCNA–mEmerald and 53BP1–mScarlet for 36 h at 30 min intervals. Shown is the PCNA–mEmerald signal together with the single-cell tracks after cell tracking.

## Source data

Source Data Figs. 3–5, Source Data Extended Data Figs. 2, 5, 6 and 8–12 and Source Data Supplementary Figs. 2, 6, 8 and 12.

## Data Availability

No restrictions apply to data availability. Additional data (original high-content microscopy, time-lapse microscopy and Live+QIBC raw data) are available on request. Gel source data are provided in Supplementary Fig. 1. Single-cell sequencing data have been uploaded to the Gene Expression Omnibus (GEO) under accession numbers GSE255874, GSE288487 and GSE288485. GENCODE human genome build GRCh38.p13 (Release 37) was used as a reference genome^77^. Source data are provided with this paper.

## Code availability

No restrictions apply to code availability. ImageJ/Fiji and MATLAB scripts that were used in this study are available at GitHub (https://github.com/AltmeyerLab/). Detailed instructions can be found at Zenodo^80^ (https://zenodo.org/records/14921691) in the file ‘Live+QIBC_guide.pdf’, together with a test dataset, which contains data for pre-imaging (Pre-imaging.zip), post-imaging (Post-imaging.zip) and iterative staining (Iterative staining.zip), and a README file (README_TestData.pdf). Moreover, there are .txt files, which correspond to analyses performed with the test dataset and the Olympus ScanR Analysis software (v.3.0.1, 3.2 and 3.3.0; analysis_live.txt and analysis_iterative_staining.txt) to be used with the MATLAB scripts without having to perform the image analysis. Finally, there is a Tracking_fiji.xls file, which is used to copy the values generated from the ‘Coord’ table in the MATLAB script to import into the tracking plugin in ImageJ/Fiji 64-bit (v.1.53f, 1.53t, 1.54f, 1.54m), so that the tracks can be overlayed with the stack of the corresponding cells.
