# Supplementary material for: Multigenerational cell tracking of DNA replication and heritable DNA damage
Source: Nature. 2025 May 21;642(8068):785–95. doi: 10.1038/s41586-025-08986-0 (PMC12176655; doi:10.1038/s41586-025-08986-0)
Supplement: Supplementary file 1 — A combined Supplementary Information PDF of 14 pages, containing Supplementary Figs. 1–12 and the corresponding legends. [file 41586_2025_8986_MOESM1_ESM.pdf]

nature portfolio

https://doi.org/10.1038/s41586-025-08986-0

Supplementary information

Multigenerational cell tracking of DNA replication and heritable DNA damage

In the format provided by the authors and unedited

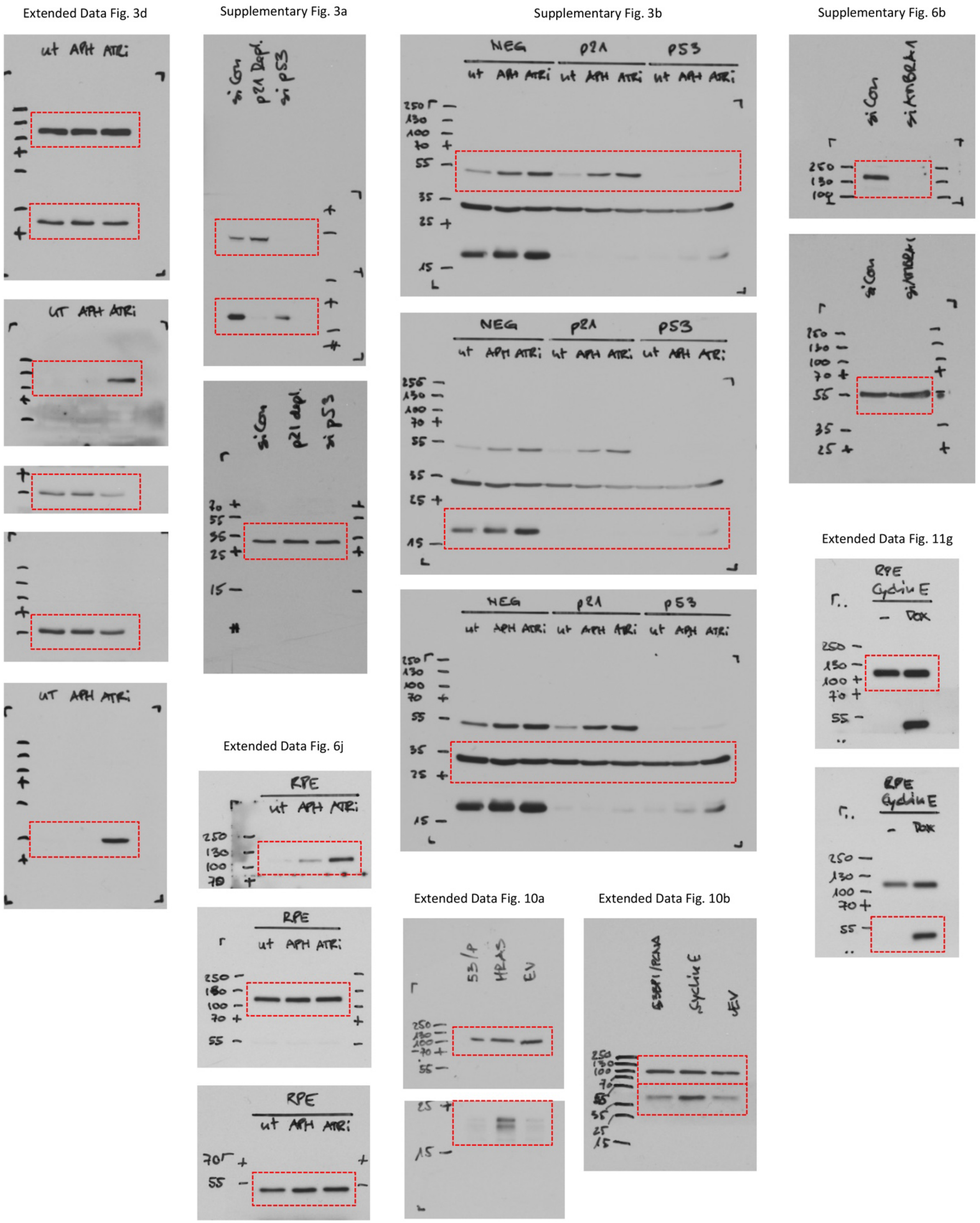

Supplementary Figure 1. Original Western blot scans. Western blot scans corresponding to figure panels, with the regions displayed in figure panels indicated by dashed red boxes. To enable detection of multiple target proteins on the same membrane without stripping (typically 2-3 target proteins per membrane with sufficiently distinct molecular weight), membranes were cut horizontally, each piece was probed with specific primary and secondary antibodies, and the membrane was then reassembled for ECL detection. Loading controls were run on the same gel and probed on the same membrane.

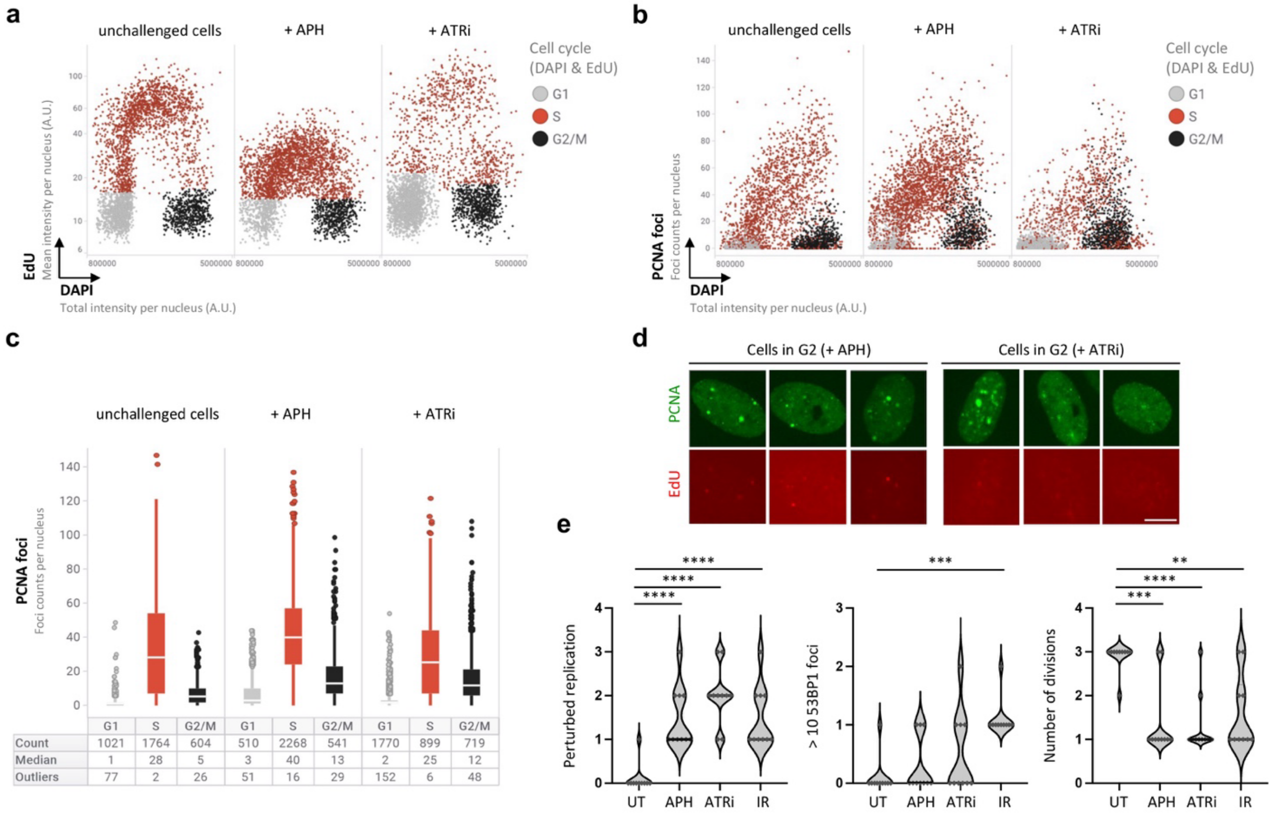

Supplementary Figure 2. Replication stress perturbs cell cycle phase boundaries. a, QIBC analysis of DNA content (DAPI) and EdU incorporation in unchallenged 53BP1-PCNA U-2 OS cells and in 53BP1-PCNA U-2 OS cells treated with 0.2 µM aphidicolin (APH) or 1 µM of ATR inhibitor (ATRi) for 24 h. Cell cycle staging as indicated by the color code. **b,** QIBC analysis of endogenous PCNA foci across the cell cycle, color coded by cell cycle according to (**a**). **c,** Box plots of endogenous PCNA foci in different phases of the cell cycle according to cell cycle gating from (**a**). **d,** Example images of endogenous PCNA foci in G2 cells after APH or ATRi treatment, according to cell cycle gating from (**a**), together with the associated EdU signals. n > 3000 cells per condition. Box plot limits in (**c**) indicate 25th percentile (Q1) and 75th percentile (Q3); boxes represent interquartile range (IQR, Q3-Q1) with medians (solid lines). Whiskers define lower and upper adjacent value; dots show outliers greater than Q3 + 1.5xIQR. Scale bar, 10 µm. **e,** Cell lineage categorization based on the indicated parameters, obtained from 10 lineages per condition, each one imaged for 55 h. One-way ANOVA followed by Tukey's test.

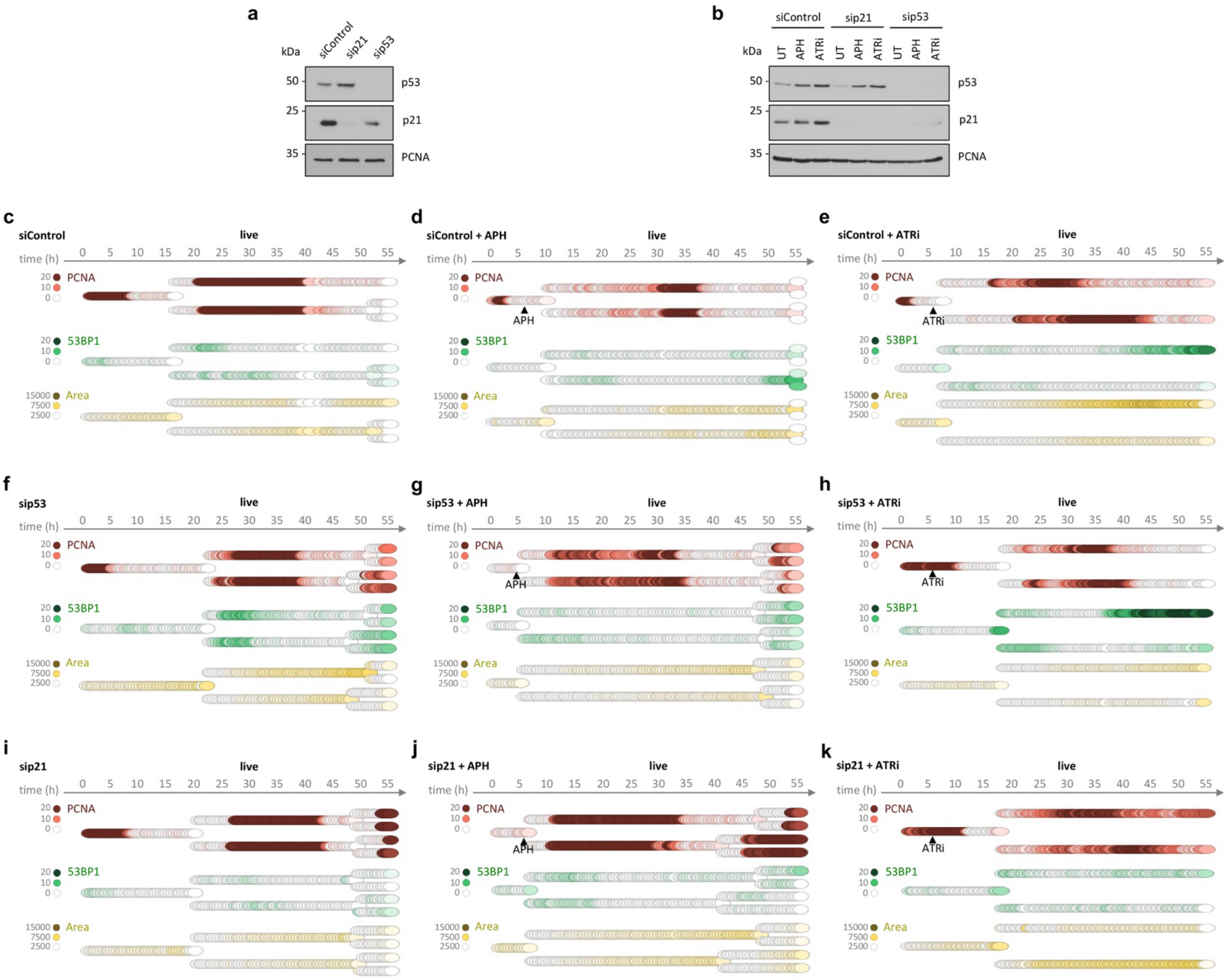

Supplementary Figure 3. Single cell tracks of cells depleted of p53 or p21 after replication stress. a, Western blot analysis of untreated U-2 OS cells depleted of p21 or p53. **b,** Western blot analysis of U-2 OS cells depleted of p21 or p53 that were either untreated or treated with 0.2 µM aphidicolin or 1 µM of ATR inhibitor for 48 h. **c,** Single cell lineage from untreated 53BP1-PCNA U-2 OS cells transfected with non-targeting control siRNA. PCNA foci, 53BP1 foci and area of the nucleus over 55 h of live imaging are depicted. **d,** Single cell lineage from 53BP1-PCNA U-2 OS cells transfected with non-targeting control siRNA and treated with 0.2 µM aphidicolin 6 h post beginning of live imaging. PCNA foci, 53BP1 foci and area of the nucleus over 55 h of live imaging are depicted. **e,** Single cell lineage from 53BP1-PCNA U-2 OS cells transfected with non-targeting control siRNA and treated with 1 µM ATRi 6 h post beginning of live imaging. PCNA foci, 53BP1 foci and area of the nucleus over 55 h of live imaging are depicted. **f,** Single cell lineage from untreated 53BP1-PCNA U-2 OS cells depleted of p53. PCNA foci, 53BP1 foci and area of the nucleus over 55 h of live imaging are depicted. **g,** Single cell lineage from 53BP1-PCNA U-2 OS cells depleted of p53 and treated with 0.2 µM aphidicolin 6 h post beginning of live imaging. PCNA foci, 53BP1 foci and area of the nucleus over 55 h of live imaging are depicted. **h,** Single cell lineage from 53BP1-PCNA U-2 OS cells depleted of p53 and treated with 1 µM ATRi 6 h post beginning of live imaging. PCNA foci, 53BP1 foci and area of the nucleus over 55 h of live imaging are depicted. **i,** Single cell lineage from untreated 53BP1-PCNA U-2 OS cells depleted of p21. PCNA foci, 53BP1 foci and area of the nucleus over 55 h of live imaging are depicted. **j,** Single cell lineage from 53BP1-PCNA U-2 OS cells depleted of p21 and treated with 0.2 µM aphidicolin 6 h post beginning of live imaging. PCNA foci, 53BP1 foci and area of the nucleus over 55 h of live imaging are depicted. **k,** Single cell lineage from 53BP1-PCNA U-2 OS cells depleted of p21 and treated with 1 µM ATRi 6 h post beginning of live imaging. PCNA foci, 53BP1 foci and area of the nucleus over 55 h of live imaging are depicted. Drugs in (**c-k**) were removed after 24 h. For gel source data, see Supplementary Figure 1.

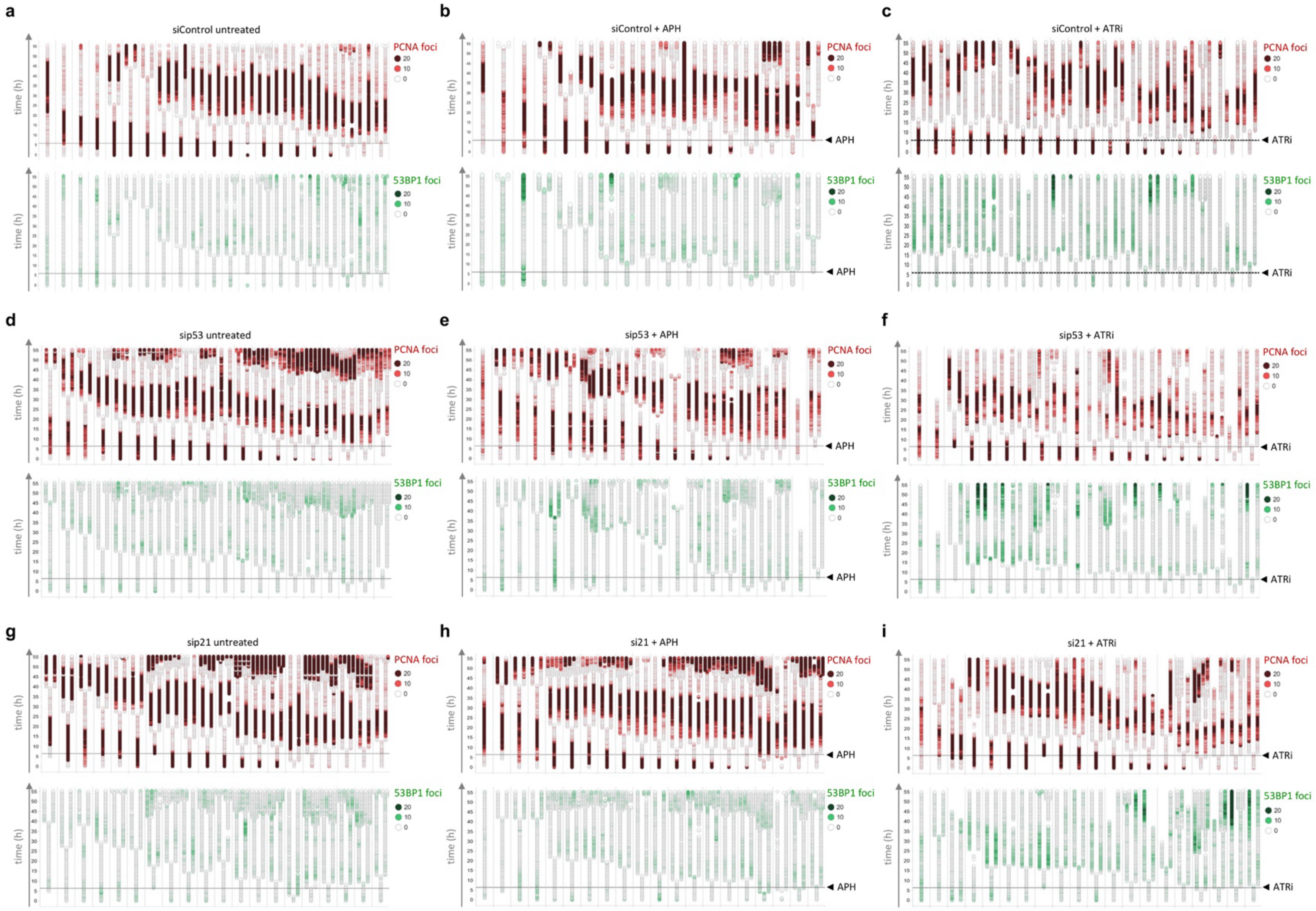

Supplementary Figure 4. *In silico* sorted single cell tracks of cells depleted of p53 or p21 after replication stress. a, *In silico* sorted single cell lineages from a 55 h experiment of untreated 53BP1-PCNA U-2 OS cells transfected with non-targeting control siRNA. PCNA foci and 53BP1 foci are depicted.
b, *In silico* sorted single cell lineages from a 55 h experiment of 53BP1-PCNA U-2 OS cells transfected with non-targeting control siRNA and treated with 0.2 µM aphidicolin 6 h post beginning of live imaging. PCNA foci and 53BP1 foci are depicted. **c,** *In silico* sorted single cell lineages from a 55 h experiment of 53BP1-PCNA U-2 OS cells transfected with non-targeting control siRNA and treated with 1 µM ATRi 6 h post beginning of live imaging. PCNA foci and 53BP1 foci are depicted. **d,** *In silico* sorted single cell lineages from a 55 h experiment of untreated 53BP1-PCNA U-2 OS cells depleted of p53. PCNA foci and 53BP1 foci are depicted. **e,** *In silico* sorted single cell lineages from a 55 h experiment of 53BP1-PCNA U-2 OS cells depleted of p53 and treated with 0.2 µM aphidicolin 6 h post beginning of live imaging. PCNA foci and 53BP1 foci are depicted. **f,** *In silico* sorted single cell lineages from a 55 h experiment of 53BP1-PCNA U-2 OS cells depleted of p53 and treated with 1 µM ATRi 6 h post beginning of live imaging. PCNA foci and 53BP1 foci are depicted. **g,** *In silico* sorted single cell lineages from a 55 h experiment of untreated 53BP1-PCNA U-2 OS cells depleted of p21. PCNA foci and 53BP1 foci are depicted. **h,** *In silico* sorted single cell lineages from a 55 h experiment of 53BP1-PCNA U-2 OS cells depleted of p21 and treated with 0.2 µM aphidicolin 6 h post beginning of live imaging. PCNA foci and 53BP1 foci are depicted. **i,** *In silico* sorted single cell lineages from a 55 h experiment of 53BP1-PCNA U-2 OS cells depleted of p21 and treated with 1 µM ATRi 6 h post beginning of live imaging. PCNA foci and 53BP1 foci are depicted; n > 16 lineages per condition. Drugs were removed after 24 h.

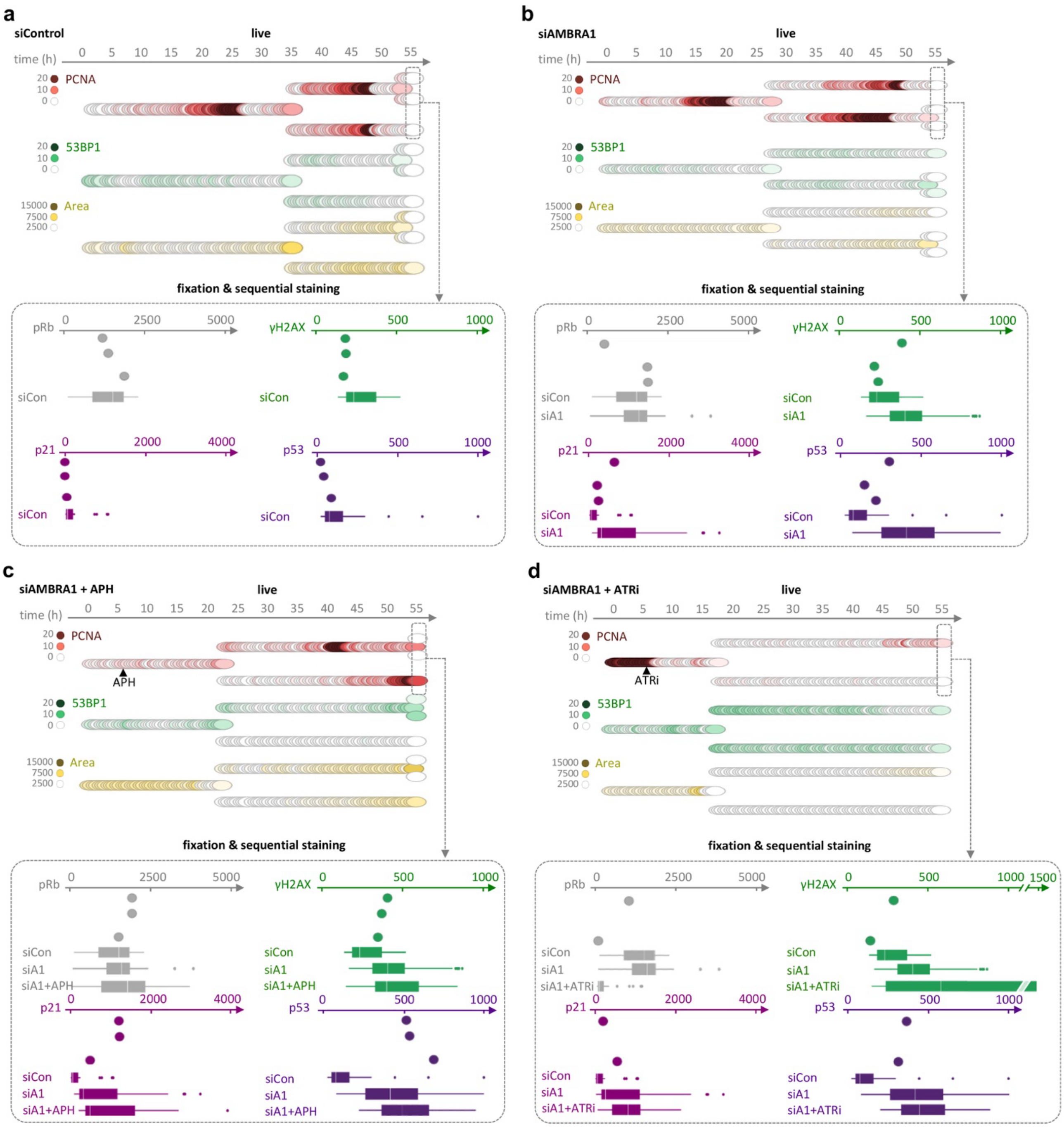

Supplementary Figure 5. Single cell tracks of cells depleted of AMBRA1. **a,** Single cell lineage from untreated 53BP1-PCNA U-2 OS cells transfected with non-targeting control siRNA. PCNA foci, 53BP1 foci and area of the nucleus over 55 h of live imaging are depicted. Sequential staining intensities from individual cells of the lineage and mean intensities from the corresponding reference population are depicted for the markers pRB, γH2AX, p21 and p53. **b,** Single cell lineage from untreated 53BP1-PCNA U-2 OS cells depleted of AMBRA1. PCNA foci, 53BP1 foci and area of the nucleus over 55 h of live imaging are depicted. Sequential staining intensities from individual cells of the lineage and mean intensities from the corresponding reference populations are depicted for the markers pRB, γH2AX, p21 and p53. **c,** Single cell lineage from 53BP1-PCNA U-2 OS cells depleted of AMBRA1 and treated with 0.2 µM aphidicolin 6 h post beginning of live imaging. PCNA foci, 53BP1 foci and area of the nucleus over 55 h of live imaging are depicted. Sequential staining intensities from individual cells of the lineage and mean intensities from the corresponding reference populations are depicted for the markers pRB, γH2AX, p21 and p53. **d,** Single cell lineage from 53BP1-PCNA U-2 OS cells depleted of AMBRA1 and treated with 1 µM ATR inhibitor 6 h post beginning of live imaging. PCNA foci, 53BP1 foci and area of the nucleus over 55 h of live imaging are depicted. Sequential staining intensities from individual cells of the lineage and mean intensities from the corresponding reference populations are depicted for the markers pRB, γH2AX, p21 and p53. Box plot limits indicate 25th percentile (Q1) and 75th percentile (Q3); boxes represent interquartile range (IQR, Q3-Q1) with medians (solid lines). Whiskers define lower and upper adjacent value; dots show outliers greater than Q3 + 1.5xIQR; n > 24 end-point measurements per sample.

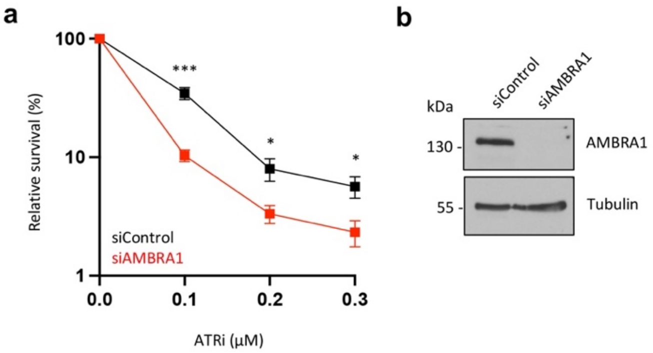

Supplementary Figure 6. Sensitization of AMBRA1-depleted cells to ATRi. a, Clonogenic survival assay in U-2 OS cells depleted of AMBRA1 and exposed to increasing concentrations of ATR inhibitor. Relative colony formation is shown. Mean ± SD from n = 3 replicates is depicted. Statistical analysis was performed with two-tailed unpaired t-test. **b,** Western blot analysis of U-2 OS cells depleted of AMBRA1. For gel source data, see Supplementary Figure 1.

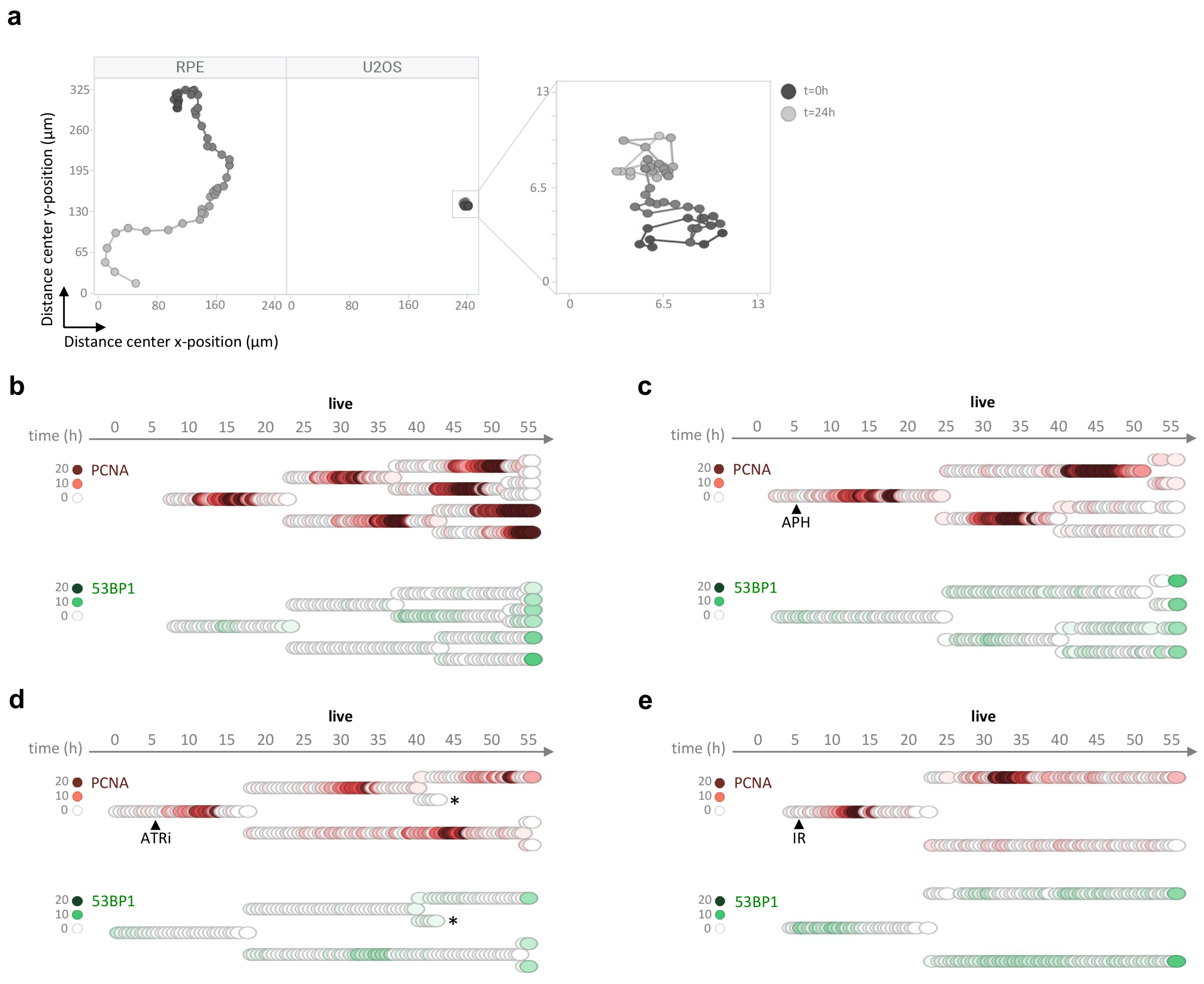

Supplementary Figure 7. Single cell tracks from RPE-1 cells treated with genotoxic agents. a, Motility comparison between 53BP1-PCNA RPE-1 and 53BP1-PCNA U-2 OS cells. Example cells imaged for 24 h with their center x/y positions plotted as a function of time. **b,** Single cell lineage from untreated 53BP1-PCNA RPE-1 cells. PCNA pattern and 53BP1 foci over 55 h of live imaging are depicted. **c,** Single cell lineage from 53BP1-PCNA RPE-1 cells treated with 0.2 µM aphidicolin. PCNA pattern and 53BP1 foci over 55 h of live imaging are depicted. **d,** Single cell lineage from 53BP1-PCNA RPE-1 cells treated with 1 µM ATRi. PCNA pattern and 53BP1 foci over 55 h of live imaging are depicted. The asterisk marks a cell that moved out of the imaged fields of view. **e,** Single cell lineage from 53BP1-PCNA RPE-1 cells treated with 4 Gy IR. PCNA pattern and 53BP1 foci over 55 h of live imaging are depicted. Drugs in (**b-d**) were washed out after 24 h.

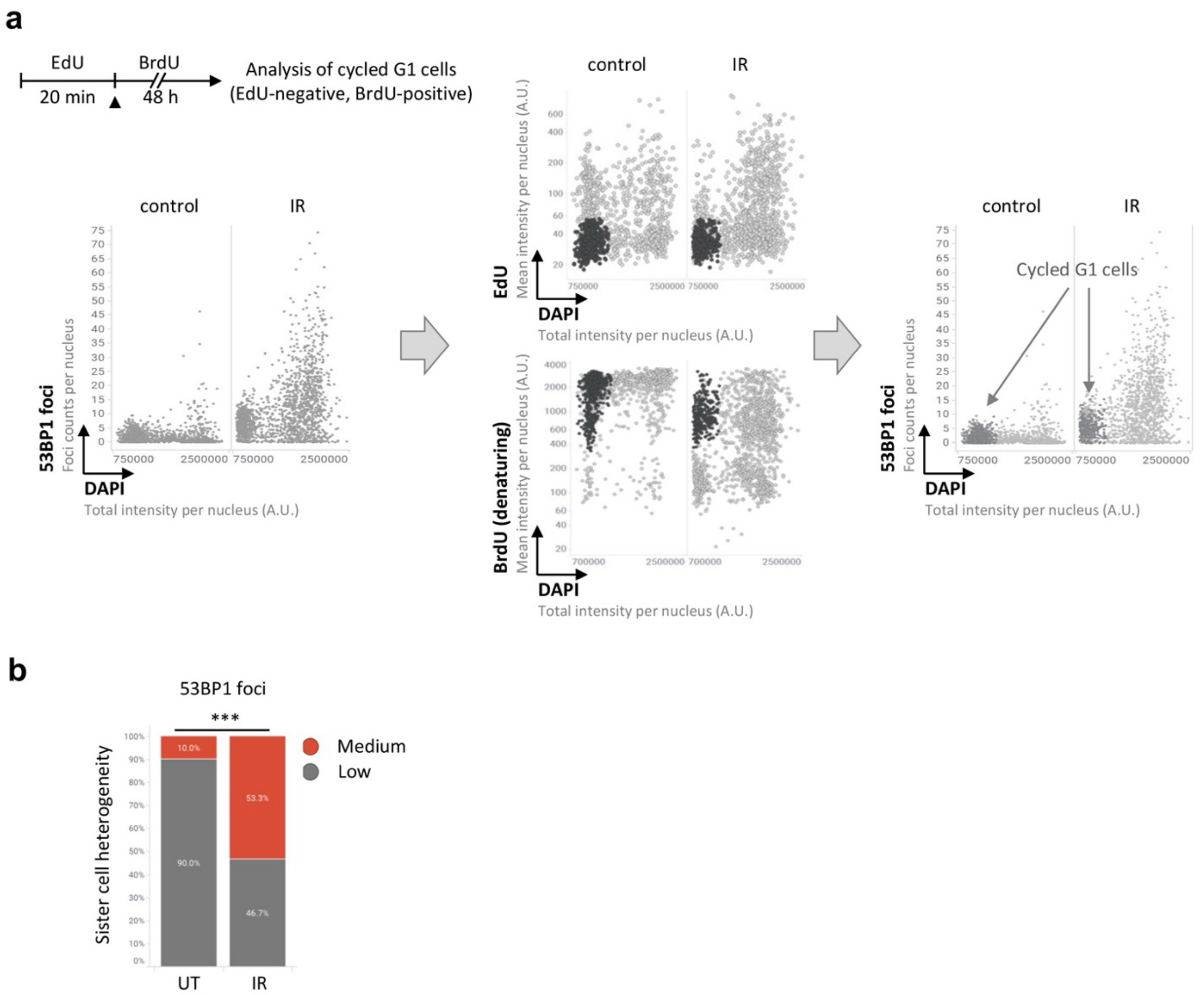

Supplementary Figure 8. DNA damage in G1 generates heritable lesions associated with sister cell asymmetry in the next cell generation. a, QIBC-derived cell cycle resolved scatter plot of IR (4 Gy) induced 53BP1 foci in the next G1 phase. U-2 OS cells were pulsed with 10 µM EdU for 20 min before irradiation and then allowed to cycle in the presence of 10 µM BrdU for 48 h to overcome the G2/M checkpoint. DNA was denatured for BrdU detection and cycled G1 cells negative for EdU and positive for BrdU were analyzed. The first graph on the left shows the total cell populations and their 53BP1 foci counts against DNA content measured by DAPI. The second graph in the middle shows the selection of EdU-negative, BrdU-positive (cycled) cells with a 2N (G1) DNA content at the time of analysis (selected cells highlighted in black). The third graph on the right shows again the total cell populations and their 53BP1 foci counts against DNA content measured by DAPI, but now with the selected cells from the middle graph highlighted in dark grey. n > 1000 cells per condition. **b,** Sister cell heterogeneity in 53BP1-PCNA RPE-1 cells treated with IR in G1. 53BP1 foci sister cell heterogeneity was scored in cells that received IR in the previous G1 phase of the cell cycle during 55 h of live imaging. n = 60 sister cells for UT and n = 60 sister cells for IR. Statistical analysis was performed with a Fisher's exact test.

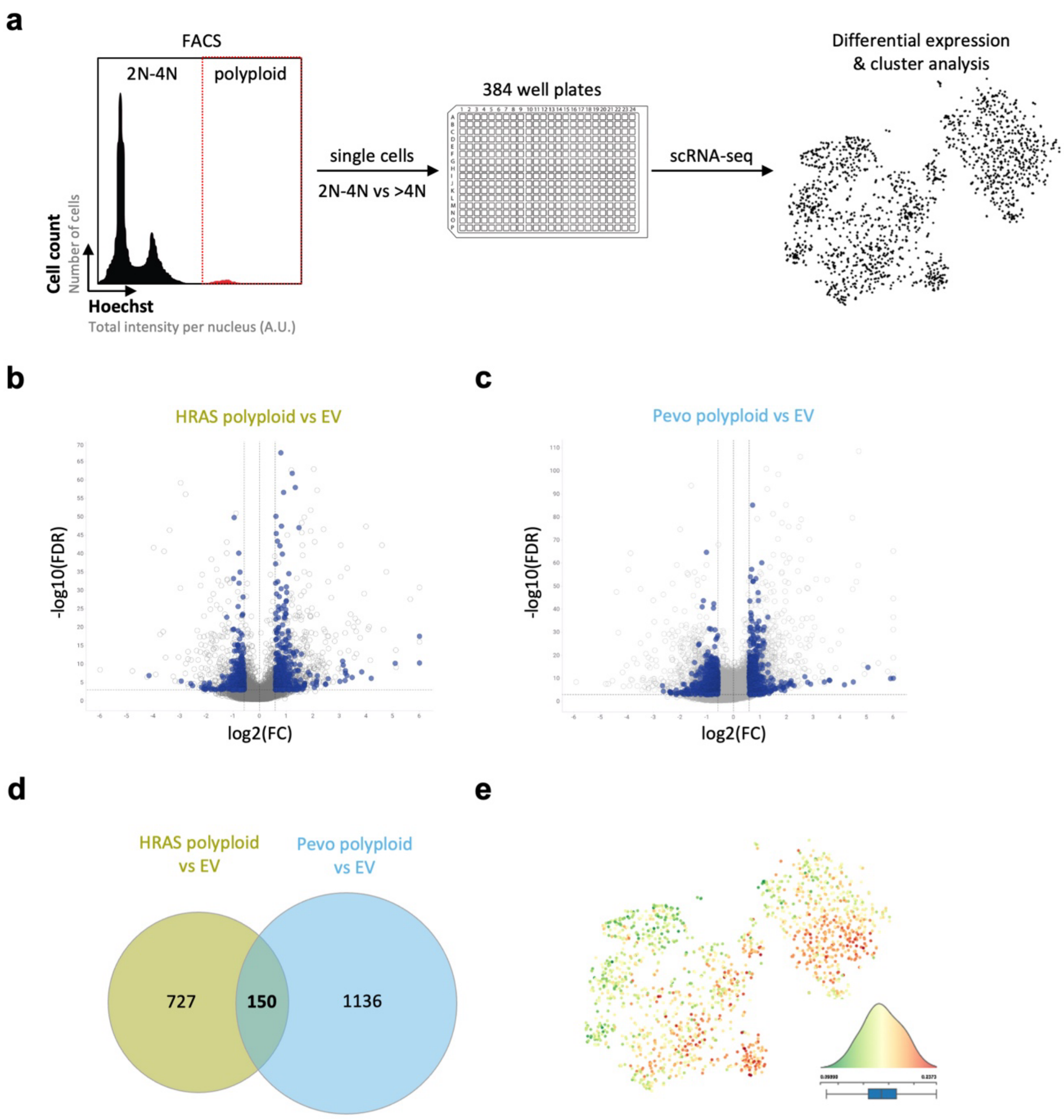

Supplementary Figure 9. scRNA-seq reveals sub-clusters among polyploid cells. a, Schematic representation of the strategy of the single cell RNA sequencing experiment. Live cells were stained with Hoechst and single cells were isolated by FACS into 384 well plates. Cells were taken both from the 2N-4N control population as well as the >4N population. **b,** Differential expression analysis of the HRAS polyploid sample vs the EV sample (shown in grey). A filtering step was included to deselect genes that were also upregulated in the HRAS 2N-4N vs EV sample (selected genes shown in blue, deselected ones in grey). FDR < 0.001, FC > 1.5 (dotted lines). **c,** Differential expression analysis of the Pevonedistat-treated polyploid sample vs the EV sample (shown in grey). A filtering step was included to deselect genes that were also upregulated in the Pevonedistat 2N-4N vs EV sample (selected genes shown in blue, deselected ones in grey). FDR < 0.001, FC > 1.5 (dotted lines). **d,** Venn diagram depicting the overlap between polyploid HRAS and Pevonedistat samples. **e,** AUCell (area under the curve) scores computed for the overlapping genes between polyploid HRAS and Pevonedistat samples, validating their enrichment in the HRAS and Pevonedistat samples of the t-SNE analysis. Q3 + 1.5IQR: 2.373e-1, Q3: 1.896e-1, median: 1.716e-1, Q1: 1.560e-1, Q1- 1.5IQR: 1.061e-1.

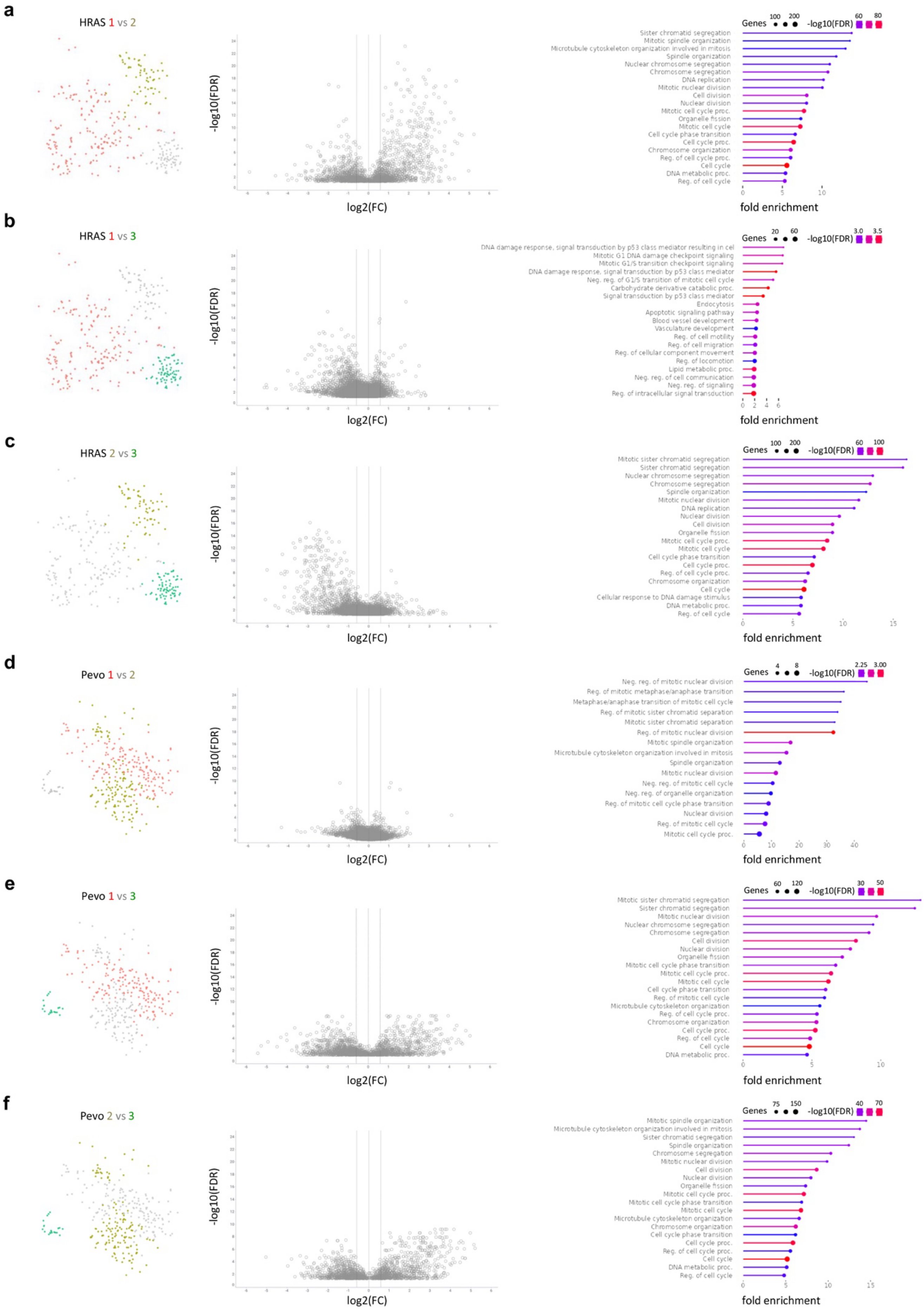
a
HRAS 1 vs 2
-log10(FDR)
log2(FC)
Genes 100 200 -log10(FDR) 60 80
Sister chromatid segregation
Mitotic spindle organization
Microtubule cytoskeleton organization involved in mitosis
Spindle organization
Nuclear chromosome segregation
Chromosome segregation
DNA replication
Mitotic nuclear division
Cell division
Nuclear division
Mitotic cell cycle proc.
Organelle fission
Mitotic cell cycle
Cell cycle phase transition
Cell cycle proc.
Chromosome organization
Reg. of cell cycle proc.
Cell cycle
DNA metabolic proc.
Reg. of cell cycle
fold enrichment
b
HRAS 1 vs 3
-log10(FDR)
log2(FC)
Genes 20 60 -log10(FDR) 3.0 3.5
DNA damage response, signal transduction by p53 class mediator resulting in cel
Mitotic G1 DNA damage checkpoint signaling
Mitotic G1/S transition checkpoint signaling
DNA damage response, signal transduction by p53 class mediator
Neg. reg. of G1/S transition of mitotic cell cycle
Carbohydrate derivative catabolic proc.
Signal transduction by p53 class mediator
Endocytosis
Apoptotic signaling pathway
Blood vessel development
Vasculature development
Reg. of cell motility
Reg. of cell migration
Reg. of cellular component movement
Reg. of locomotion
Lipid metabolic proc.
Neg. reg. of cell communication
Neg. reg. of signaling
Reg. of intracellular signal transduction
fold enrichment
c
HRAS 2 vs 3
-log10(FDR)
log2(FC)
Genes 100 200 -log10(FDR) 60 100
Mitotic sister chromatid segregation
Sister chromatid segregation
Nuclear chromosome segregation
Chromosome segregation
Spindle organization
Mitotic nuclear division
DNA replication
Nuclear division
Cell division
Organelle fission
Mitotic cell cycle proc.
Mitotic cell cycle
Cell cycle phase transition
Cell cycle proc.
Reg. of cell cycle proc.
Chromosome organization
Cell cycle
Cellular response to DNA damage stimulus
DNA metabolic proc.
Reg. of cell cycle
fold enrichment
d
Pevo 1 vs 2
-log10(FDR)
log2(FC)
Genes 4 8 -log10(FDR) 2.25 3.00
Neg. reg. of mitotic nuclear division
Reg. of mitotic metaphase/anaphase transition
Metaphase/anaphase transition of mitotic cell cycle
Reg. of mitotic sister chromatid separation
Mitotic sister chromatid separation
Reg. of mitotic nuclear division
Mitotic spindle organization
Microtubule cytoskeleton organization involved in mitosis
Spindle organization
Mitotic nuclear division
Neg. reg. of mitotic cell cycle
Neg. reg. of organelle organization
Reg. of mitotic cell cycle phase transition
Nuclear division
Reg. of mitotic cell cycle
Mitotic cell cycle proc.
fold enrichment
e
Pevo 1 vs 3
-log10(FDR)
log2(FC)
Genes 60 120 -log10(FDR) 30 50
Mitotic sister chromatid segregation
Sister chromatid segregation
Mitotic nuclear division
Nuclear chromosome segregation
Chromosome segregation
Cell division
Nuclear division
Organelle fission
Mitotic cell cycle phase transition
Mitotic cell cycle proc.
Mitotic cell cycle
Cell cycle phase transition
Reg. of mitotic cell cycle
Microtubule cytoskeleton organization
Reg. of cell cycle proc.
Chromosome organization
Cell cycle proc.
Reg. of cell cycle
Cell cycle
DNA metabolic proc.
fold enrichment
f
Pevo 2 vs 3
-log10(FDR)
log2(FC)
Genes 75 150 -log10(FDR) 40 70
Mitotic spindle organization
Microtubule cytoskeleton organization involved in mitosis
Sister chromatid segregation
Spindle organization
Chromosome segregation
Mitotic nuclear division
Cell division
Nuclear division
Organelle fission
Mitotic cell cycle proc.
Mitotic cell cycle phase transition
Mitotic cell cycle
Microtubule cytoskeleton organization
Chromosome organization
Cell cycle phase transition
Cell cycle proc.
Reg. of cell cycle proc.
Cell cycle
DNA metabolic proc.
Reg. of cell cycle
fold enrichment

Supplementary Figure 10. Differential gene expression in subclusters of polyploid cells. a, Sub-cluster analysis of cluster 1 vs 2 of HRAS polyploid U-2 OS cells with a Louvain resolution of 0.5. From left to right: t-SNE showing clusters 1 and 2 as well as the differential expression and GO analysis of the differentially expressed genes. FDR < 0.001, p-value < 0.001, FC > 1.5. **b,** Sub-cluster analysis of cluster 1 vs 3 of HRAS polyploid U-2 OS cells with a Louvain resolution of 0.5. From left to right: t-SNE showing clusters 1 and 3 as well as the differential expression and GO analysis of the differentially expressed genes. FDR < 0.001, p-value < 0.001, FC > 1.5. **c,** Sub-cluster analysis of cluster 2 vs 3 of HRAS polyploid U-2 OS cells with a Louvain resolution of 0.5. From left to right: t-SNE showing clusters 2 and 3 as well as the differential expression and GO analysis of the differentially expressed genes. FDR < 0.001, p-value < 0.001, FC > 1.5. **d,** Sub-cluster analysis of cluster 1 vs 2 of Pevonedistat polyploid U-2 OS cells with a Louvain resolution of 0.5. From left to right: t-SNE showing clusters 1 and 2 as well as the differential expression and GO analysis of the differentially expressed genes. FDR < 0.001, p-value < 0.001, FC > 1.5. **e,** Sub-cluster analysis of cluster 1 vs 3 of Pevonedistat polyploid U-2 OS cells with a Louvain resolution of 0.5. From left to right: t-SNE showing clusters 1 and 3 as well as the differential expression and GO analysis of the differentially expressed genes. FDR < 0.001, p-value < 0.001, FC > 1.5. **f,** Sub-cluster analysis of cluster 2 vs 3 of Pevonedistat polyploid U-2 OS cells with a Louvain resolution of 0.5. From left to right: t-SNE showing clusters 2 and 3 as well as the differential expression and GO analysis of the differentially expressed genes. FDR < 0.001, p-value < 0.001, FC > 1.5.

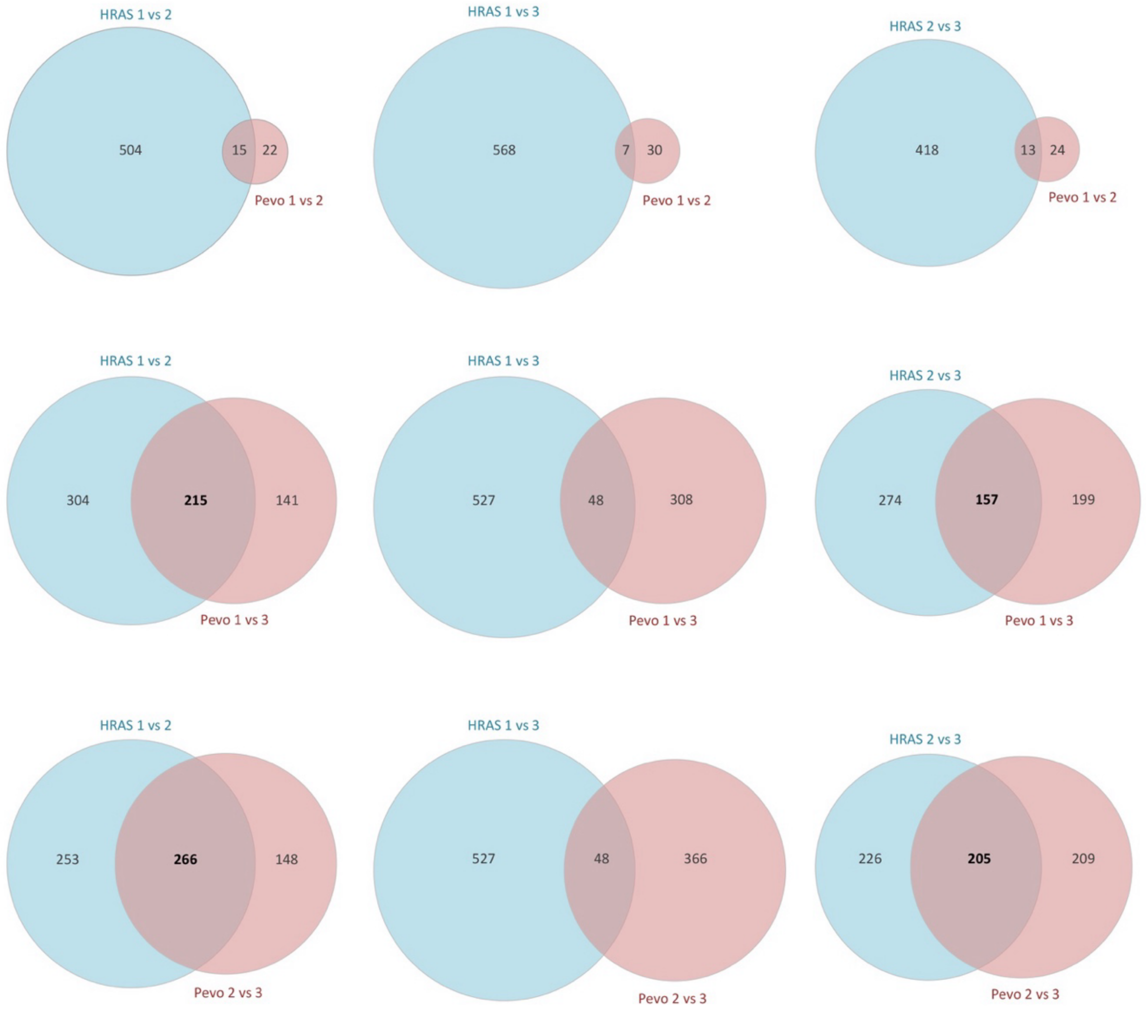

Supplementary Figure 11. Cross-comparison of differentially regulated genes in subclusters of polyploid cells. Venn diagrams showing the pairwise overlaps of differentially expressed genes in subclusters of polyploid HRAS and Pevonedistat-treated U-2 OS cells. FDR < 0.001, p-value < 0.001, FC > 1.5.

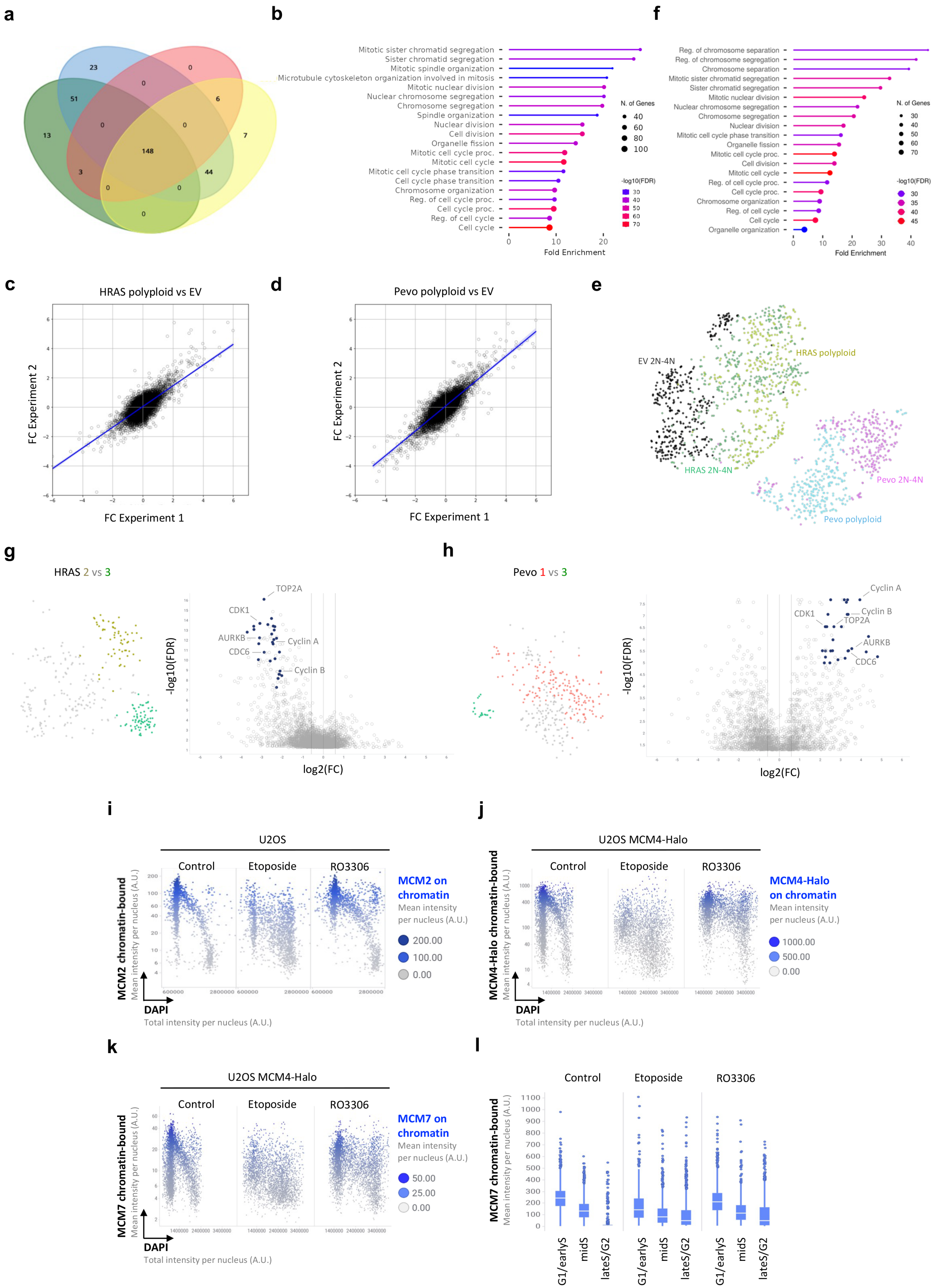
a
23
0
51
0
6
13
0
0
7
148
3
44
0
0
0
b
Mitotic sister chromatid segregation
Sister chromatid segregation
Mitotic spindle organization
Microtubule cytoskeleton organization involved in mitosis
Mitotic nuclear division
Nuclear chromosome segregation
Chromosome segregation
Spindle organization
Nuclear division
Cell division
Organelle fission
Mitotic cell cycle proc.
Mitotic cell cycle
Mitotic cell cycle phase transition
Cell cycle phase transition
Chromosome organization
Reg. of cell cycle proc.
Cell cycle proc.
Reg. of cell cycle
Cell cycle
N. of Genes
40
60
80
100
-log10(FDR)
30
40
50
60
70
Fold Enrichment
f
Reg. of chromosome separation
Reg. of chromosome segregation
Chromosome separation
Mitotic sister chromatid segregation
Sister chromatid segregation
Mitotic nuclear division
Nuclear chromosome segregation
Chromosome segregation
Nuclear division
Mitotic cell cycle phase transition
Organelle fission
Mitotic cell cycle proc.
Cell division
Mitotic cell cycle
Reg. of cell cycle proc.
Cell cycle proc.
Chromosome organization
Reg. of cell cycle
Cell cycle
Organelle organization
N. of Genes
30
40
50
60
70
-log10(FDR)
30
35
40
45
Fold Enrichment
c
HRAS polyploid vs EV
FC Experiment 2
FC Experiment 1
d
Pevo polyploid vs EV
FC Experiment 2
FC Experiment 1
e
HRAS polyploid
EV 2N-4N
HRAS 2N-4N
Pevo 2N-4N
Pevo polyploid
g
HRAS 2 vs 3
TOP2A
CDK1
AURKB
Cyclin A
CDC6
Cyclin B
-log10(FDR)
log2(FC)
h
Pevo 1 vs 3
Cyclin A
CDK1
Cyclin B
TOP2A
AURKB
CDC6
-log10(FDR)
log2(FC)
i
U2OS
Control
Etoposide
RO3306
MCM2 chromatin-bound
Mean intensity per nucleus (A.U.)
MCM2 on chromatin
Mean intensity per nucleus (A.U.)
200.00
100.00
0.00
DAPI
Total intensity per nucleus (A.U.)
j
U2OS MCM4-Halo
Control
Etoposide
RO3306
MCM4-Halo chromatin-bound
Mean intensity per nucleus (A.U.)
MCM4-Halo on chromatin
Mean intensity per nucleus (A.U.)
1000.00
500.00
0.00
DAPI
Total intensity per nucleus (A.U.)
k
U2OS MCM4-Halo
Control
Etoposide
RO3306
MCM7 chromatin-bound
Mean intensity per nucleus (A.U.)
MCM7 on chromatin
Mean intensity per nucleus (A.U.)
50.00
25.00
0.00
DAPI
Total intensity per nucleus (A.U.)
l
Control
Etoposide
RO3306
MCM7 chromatin-bound
Mean intensity per nucleus (A.U.)
G1/earlyS
midS
lateS/G2
G1/earlyS
midS
lateS/G2
G1/earlyS
midS
lateS/G2

Supplementary Figure 12. Differential gene expression in subclusters of polyploid cells. a, Venn diagram showing the overlay of the four most significant overlaps from Supplementary Figure 6, i.e. HRAS 1 vs 2 and Pevo 1 vs 3, HRAS 1 vs 2 and Pevo 2 vs 3, HRAS 2 vs 3 and Pevo 1 vs 3, HRAS 2 vs 3 and Pevo 2 vs 3. **b,** GO analysis of the 148 genes found in (**a**). **c,** Fold change (FC) comparison of differentially expressed genes in HRAS polyploid cells versus empty vector (EV) control cells from two sets of single cell samples that we sequenced by scRNA-seq and analyzed independently. **d,** Fold change (FC) comparison of differentially expressed genes in Pevonedistat-treated polyploid cells versus empty vector (EV) control cells from two sets of single cell samples that we sequenced by scRNA-seq and analyzed independently. **e,** t-SNE analysis of replicate scRNA-seq results corresponding to what is shown in Figure 5b. EV, empty vector; 2N-4N, cells with a 2N-4N DNA content; polyploid, cells with a DNA content >4N; Pevo, Pevonedistat-treated; HRAS, HRAS over-expressing cells. n > 270 cells per condition **f,** GO analysis of genes identified in the replicate scRNA-seq results corresponding to what is shown in (**b**). **g,** Differential expression analysis between subclusters 2 and 3 of polyploid HRAS cells with the most consistently deregulated genes highlighted in blue. **h,** Differential expression analysis between subclusters 1 and 3 of polyploid Pevonedistat-treated cells with the most consistently deregulated genes highlighted in blue. **i,** Cell cycle resolved MCM2 loading on chromatin in pre-extracted U-2 OS cells either untreated or treated with 20 μM of Etoposide or 5 μM of RO3306 for 24 h and then released in fresh medium for 42 h. **j,** Cell cycle resolved MCM4-Halo loading on chromatin in pre-extracted U-2 OS MCM4-Halo cells either untreated or treated with 20 μM of Etoposide or 5 μM of RO3306 for 24 h and then released in fresh medium for 42 h. **k,** Cell cycle resolved MCM7 loading on chromatin in pre-extracted U-2 OS MCM4-Halo cells either untreated or treated with 20 μM of Etoposide or 5 μM of RO3306 for 24 h and then released in fresh medium for 42 h. **l,** MCM7 loading on chromatin in cells from (**k**), gated into G1/earlyS, midS, and lateS/G2 based on DNA content. n > 2000 cells per condition. Box plot limits indicate 25th percentile (Q1) and 75th percentile (Q3); boxes represent interquartile range (IQR, Q3-Q1) with medians (solid lines). Whiskers define lower and upper adjacent value; dots show outliers greater than Q3 + 1.5xIQR.
