# Supplementary material for: Multigenerational cell tracking of DNA replication and heritable DNA damage
Source: Nature. 2025 May 21;642(8068):785–95. doi: 10.1038/s41586-025-08986-0 (PMC12176655; doi:10.1038/s41586-025-08986-0)
Supplement: Supplementary file 2 — Reporting Summary [file 41586_2025_8986_MOESM2_ESM.pdf]

nature portfolio

Corresponding author(s): Matthias Altmeyer

Last updated by author(s): 2025/03/31

Reporting Summary

Nature Portfolio wishes to improve the reproducibility of the work that we publish. This form provides structure for consistency and transparency in reporting. For further information on Nature Portfolio policies, see our Editorial Policies and the Editorial Policy Checklist.

Statistics

For all statistical analyses, confirm that the following items are present in the figure legend, table legend, main text, or Methods section.

n/a	Confirmed	
☐	☒	The exact sample size (*n*) for each experimental group/condition, given as a discrete number and unit of measurement
☐	☒	A statement on whether measurements were taken from distinct samples or whether the same sample was measured repeatedly
☐	☒	The statistical test(s) used AND whether they are one- or two-sided<br>*Only common tests should be described solely by name; describe more complex techniques in the Methods section.*
☒	☐	A description of all covariates tested
☒	☐	A description of any assumptions or corrections, such as tests of normality and adjustment for multiple comparisons
☐	☒	A full description of the statistical parameters including central tendency (e.g. means) or other basic estimates (e.g. regression coefficient) AND variation (e.g. standard deviation) or associated estimates of uncertainty (e.g. confidence intervals)
☐	☒	For null hypothesis testing, the test statistic (e.g. *F*, *t*, *r*) with confidence intervals, effect sizes, degrees of freedom and *P* value noted<br>*Give P values as exact values whenever suitable.*
☒	☐	For Bayesian analysis, information on the choice of priors and Markov chain Monte Carlo settings
☒	☐	For hierarchical and complex designs, identification of the appropriate level for tests and full reporting of outcomes
☒	☐	Estimates of effect sizes (e.g. Cohen's *d*, Pearson's *r*), indicating how they were calculated

Our web collection on statistics for biologists contains articles on many of the points above.

Software and code

Policy information about availability of computer code

Data collection	HCI: Olympus ScanR Image Acquisition software (versions 3.0.1, 3.2 and 3.3.0); GE InCell Analyzer 2500 V7.4 acquisition software; Leica THUNDER (Las X 3.7.6.25997)<br>Western blot: OPTIMAX X-Ray Film Processor (PROTEC Medizintechnik GmbH & Co. KG)<br>Agarose gel imaging: Infinity ST5 Xpress v16.16d
Data analysis	Microscopy image analysis: Olympus ScanR Analysis (versions 3.0.1, 3.2 and 3.3.0); ImageJ/Fiji 64-bit (versions 1.53f, 1.53t, 1.54f, 1.54m);<br>Microscopy data visualization: TIBCO Spotfire (versions 7.9.1, 10.10.1)<br>Statistical analysis and data visualization: Graphpad Prism (versions 9 and 10)<br>Matlab: MathWorks MATLAB R2019b, R2020b, R2023a<br>RNA sequencing analysis: Illumina bcl2fastq Conversion Software (v2.20.0.422); STARSolo 2.7.10b; STARSolo 2.7.11b; ShinyGO (versions V0.77 and V0.80); BioTuring BBrowser X software (BioTuring Inc., San Diego, CA, USA); R version 4.4.2; Bioconductor version 3.20; FACS Diva Software v8.0.1<br>Custom scripts: https://github.com/AltmeyerLab/SingleCellTracking_Timelapse; https://github.com/AltmeyerLab/SingleCellTracking_Multiplex-Alignment; https://github.com/AltmeyerLab/MatlabTracking (Cell_tracking.m & KeyDataExtractions.m); https://zenodo.org/records/14921691

For manuscripts utilizing custom algorithms or software that are central to the research but not yet described in published literature, software must be made available to editors and reviewers. We strongly encourage code deposition in a community repository (e.g. GitHub). See the Nature Portfolio guidelines for submitting code & software for further information.

Data

Policy information about availability of data

All manuscripts must include a data availability statement. This statement should provide the following information, where applicable:

- Accession codes, unique identifiers, or web links for publicly available datasets
- A description of any restrictions on data availability
- For clinical datasets or third party data, please ensure that the statement adheres to our policy

Sequencing data were deposited at Gene Expression Omnibus (GEO)
GSE255874: https://www.ncbi.nlm.nih.gov/geo/query/acc.cgi?acc=GSE255874
GSE288487: https://www.ncbi.nlm.nih.gov/geo/query/acc.cgi?acc=GSE288487
GSE288485: https://www.ncbi.nlm.nih.gov/geo/query/acc.cgi?acc=GSE288485
GENCODE human genome build GRCh38.p13 (Release 37) was used as reference genome.

Research involving human participants, their data, or biological material

Policy information about studies with human participants or human data. See also policy information about sex, gender (identity/presentation), and sexual orientation and race, ethnicity and racism.

Reporting on sex and gender	Not applicable
Reporting on race, ethnicity, or other socially relevant groupings	Not applicable
Population characteristics	Not applicable
Recruitment	Not applicable
Ethics oversight	Not applicable

Note that full information on the approval of the study protocol must also be provided in the manuscript.

Field-specific reporting

Please select the one below that is the best fit for your research. If you are not sure, read the appropriate sections before making your selection.

☒ Life sciences ☐ Behavioural & social sciences ☐ Ecological, evolutionary & environmental sciences

For a reference copy of the document with all sections, see nature.com/documents/nr-reporting-summary-flat.pdf

Life sciences study design

All studies must disclose on these points even when the disclosure is negative.

Sample size	This study did not include animal models or human participants and sample sizes were determined based on current standards in the field (e.g. Toledo et al., Cell 2013 Nov 21; 155(5):1088-1103; Michelena et al., Nat Commun. 2018 Jul 11; 9(1):2678; Sedlackova et al., Nature, 2020 Oct 21; 587, 297-302; Lezaja et al., Nat Commun. 2021 Jun 22; 12(1):3827). For QIBC experiments: >500 cells per condition; for DNA fiber analyses: >100 fibers per condition; for Live+QIBC experiments: >20 cell lineages corresponding to up to 40 daughter and up to 80 granddaughter cells per condition for U-2 OS cells and >10 cell lineages for RPE-1 cells; for single cell sequencing: 384 well plates were used per condition.
Data exclusions	No relevant data were excluded from this study.
Replication	Experiments were performed in at least 2-3 biological replicates and experimental findings were reliably reproduced.
Randomization	Experiments were performed with asynchronously cycling cell populations and cultures serving as control or experimental groups, respectively, were randomly assigned. For each condition multiple non-overlapping fields of view using an evenly distributed standard grid were acquired during microscopy.
Blinding	Data collection and analysis of microscopy experiments was conducted using automated or semi-automated image acquisition and analysis software. No further blinding was applied and no animals or human research participants or samples were involved in the study.

Reporting for specific materials, systems and methods

We require information from authors about some types of materials, experimental systems and methods used in many studies. Here, indicate whether each material, system or method listed is relevant to your study. If you are not sure if a list item applies to your research, read the appropriate section before selecting a response.

Materials & experimental systems

n/a	Involved in the study
☐	☒ Antibodies
☐	☒ Eukaryotic cell lines
☒	☐ Palaeontology and archaeology
☒	☐ Animals and other organisms
☒	☐ Clinical data
☒	☐ Dual use research of concern
☒	☐ Plants

Methods

n/a	Involved in the study
☒	☐ ChIP-seq
☒	☐ Flow cytometry
☒	☐ MRI-based neuroimaging

Antibodies

Antibodies used

Primary antibodies used in this study:
H2AX phospho S139 (Biolegend, 613401, diluted 1:1000 for IF)
pRB (Cell Signaling Technologies, 8516S, diluted 1:500 for IF)
p21 (Abcam, ab109520, diluted 1:500 for IF and WB)
p53 (Thermo Fisher, AHO0152, diluted 1:500 for IF and WB)
53BP1 (Novus Biologicals, NB100-304, diluted 1:1000 for IF)
Cyclin A (Abcam, ab181591, diluted 1:500 for IF)
Cyclin E (Abcam, ab208696, diluted 1:1000 for WB)
HRAS (GeneTex, GTX116041, diluted 1:500 for WB)
PCNA (Santa Cruz, sc-56, diluted 1:2000 for WB)
Tubulin (Sigma Aldrich, T6199, 1:5000 for WB)
AMBRA1 (Santa Cruz, sc-398204, diluted 1:500 for WB)
KAP1 phospho-S824 (Abcam, ab70369, diluted 1:500 for WB)
KAP1 (Bethyl, A300-274A, diluted 1:2000 for WB)
CHK1 phospho-S296 (Abcam, ab79758, diluted 1:500 for WB)
CHK1 (Abcam, ab40866, diluted 1:500 for WB)
RPA32 phospho-S4/8 (Bethyl, A300-245A, 1:1000 diluted for WB)
RPA32/RPA2 (Abcam, ab2175, diluted 1:500 for WB)
BrdU (Abcam, ab6326, diluted 1:250 for IF)
BrdU (BD Biosciences, 347580, 1:80 for IF)
MCM2 (Santa Cruz, sc-9839, 1:100 for IF)
MCM7 (Santa Cruz, sc-9966, 1:100 for IF)

Secondary antibodies used in this study:
Alexa Fluor 647 Goat Anti-Rabbit (Thermo Fisher Scientific, A21244, diluted 1:500 for IF)
Alexa Fluor 647 Goat Anti-Mouse (Thermo Fisher Scientific, A21235, diluted 1:500 for IF)
Alexa Fluor 568 Goat Anti-Rabbit (Thermo Fisher Scientific, A11036, diluted 1:500 for IF)
Alexa Fluor 568 Goat Anti-Mouse (Thermo Fisher Scientific, A11031, diluted 1:500 for IF)
Alexa Fluor 555 Goat Anti-Rat (Thermo Fisher Scientific, A21434, diluted 1:250 for IF)
Alexa Fluor 488 Goat Anti-Rabbit (Thermo Fisher Scientific, A11034, diluted 1:500 for IF)
Alexa Fluor 488 Goat Anti-Mouse (Thermo Fisher Scientific, A11029, diluted 1:500 for IF)
Alexa Fluor 488 Rabbit Anti-Goat (Thermo Fisher Scientific, A11078, diluted 1:500 for IF)
Goat Anti-Rabbit IgG Antibody (H+L), Peroxidase (Vector Laboratories, PI-1000-1, diluted 1:10000 for WB)
Goat Anti-Mouse IgG Antibody (H+L), Peroxidase (Vector Laboratories, PI-2000-1, diluted 1:10000 for WB)

Validation

H2AX phospho S139 (Biolegend, 613401) was previously validated by ATM inhibition (Lezaja et al., Nat. Commun. 12(1); 3827 (2021)). pRB (Cell Signaling Technologies, 8516S) was previously validated by immunofluorescence (Liu et al., Nat. Commun. 11(1):5305 (2020)). p21 (Abcam, ab109520) was validated in this study by p21 knockdown analyzed by immunofluorescence and western blot. p53 (Thermo Fisher, AHO0152) was validated in this study by p53 knockdown analyzed by immunofluorescence and western blot. 53BP1 (Novus Biologicals, NB100-304) was previously validated by the manufacturer by gamma irradiation and by immunofluorescence and by 53BP1 knockdown (Han et al., Sci. Adv. 14;1(7):e1500454 (2015)). Cyclin A (Abcam, ab181591) was previously validated by co-staining with other cell cycle markers and used for immunofluorescence (Lezaja et al., Nat. Commun. 12(1); 3827 (2021)). Cyclin E (Abcam, ab208696) was validated by knockout from the manufacturer. HRAS (GeneTex, GTX116041) was validated in this study by western blot. PCNA (Santa Cruz, sc-56) was validated by knockout and western blot (Dietsch et al., BioTechniques. 62(2):80-82 (2017)). Tubulin (Sigma Aldrich, T6199) was validated by the company. AMBRA1 (Abcam, sc-398204) was validated in this study by knockdown analyzed by western blot. KAP1 phospho-S824 (Abcam, ab70369) was previously validated (Teloni et al., Mol Cell. 3(4):670-683 (2019); Michelena et al., J. Cell Biol. 218(9): 2865-2875 (2019)). KAP1 (Bethyl, A300-274A) was previously validated by the manufacturer

and used in recent studies as loading control (Uhlen et al., Nat. Methods.12(10):823 (2016); Spies et al. Nat. Cell. Bio.21(4):487-497 (2019); Teloni et al., Mol Cell. 3(4):670-683 (2019); Silva et al., Nat.Commun. 10(1),2253(2019); Spegg et al., Nat. Struc.t Mol. Biol. 30, 451-462 (2023)). CHK1 phospho-S296 (Abcam, ab79758) was previously validated by CHK1i (Michelena et al., J. Cell Biol. 218(9): 2865-2875 (2019)). CHK1 (Abcam, ab40866) was previously validated (Michelena et al., J. Cell Biol. 218(9): 2865-2875 (2019)). RPA32 phospho-S4/8 (Bethyl, A300-245) was previously validated by Western Blot (Zhang et al., Nat. Commun. 13, 6907 (2022)). RPA32/RPA2 (Abcam, ab2175) was previously validated by knockdown (Mylers et al., Proc Natl Acad Sci U S A. 2016 Mar 1;113(9):E1170-9; Lezaja et al., Nat.Commun. 12, 3827 (2021)), it was further validated by microscopy analysis of co-localization with GFP-RPA2 signal (Spegg et al., Nat. Struct. Mol. Biol. 30, 451-462(2023)). BrdU (Abcam, ab6326) was previously validated by the manufacturer by immunofluorescence in untreated and BrdU-treated cells. BrdU (BD Biosciences, 347580) was previously validated by the manufacturer by immunofluorescence followed by fluorescence microscopy and flow cytometry. MCM2 (Santa Cruz, sc-9839) was previously validated by RNAi experiments of CUL4B (Zou et al., J. Cell Biol. 200: 743-756 (2013)). MCM7 (Santa Cruz, sc-9839) was previously validated by the manufacturer by Western blot of whole cell extracts from various cell lines. It has also been used by various studies for example: Sedlackova et al., Nat. Commun. 13, 6090 (2022).

Secondary antibodies used in this study:
All secondary antibodies were validated by the manufacturers and used in recent studies (Spegg et al., Nat. Struct. Mol. Biol. 30(4):451-462 (2023), Lezaja et al., Nat. Commun. 12(1); 3827 (2021), Teloni et al., Mol. Cell. 72(4):670-683 (2019)).

Eukaryotic cell lines

Policy information about cell lines and Sex and Gender in Research

Cell line source(s)

U-2 OS cell line: ATCC (HTB-96, RRID:CVCL_0042)
U-2 OS H2B-GFP cell line: kindly provided by Dr. Daniel Gerlich (IMBA, Vienna, Austria)
U-2 OS GFP 53BP1 cell line: kindly provided by Dr. Jiri Lukas (University of Copenhagen, Denmark)
U-2 OS 53BP1-GFP-AID-DIvA cell line: kindly provided by Dr. Gaëlle Legube (CBI Toulouse, France)
U-2 OS 53BP1-mScarlet PCNA-mEmerald cell line: this study
U-2 OS 53BP1-mScarlet PCNA-mEmerald cell line (Empty vector-EV): this study
U-2 OS 53BP1-mScarlet PCNA-mEmerald cell line (HRAS overexpression): this study
U-2 OS 53BP1-mScarlet PCNA-mEmerald cell line (Cyclin E overexpression): this study
RPE-1 cell line: ATCC (RRID:CVCL_4388)
RPE-1 53BP1-mScarlet PCNA-mEmerald cell line: this study
RPE-1 53BP1-mScarlet PCNA-mEmerald cell line for Dox-inducible Cyclin E overexpression: this study
HCT 116 cell line: ATCC (RRID:CVCL_0291)
HEK 293T cell line: ATCC (RRID:CVCL_0063)

Authentication

The parental U-2 OS and RPE-1 cell lines were authenticated by STR profiling (last authentication in March 2025, both with 100 % matched identity). The derived cell lines were not additionally authenticated. The HCT 116 and HEK 293 T cells were not authenticated.

Mycoplasma contamination

All cell lines used in this study were routinely tested every 4-6 weeks for mycoplasma contamination and always scored negative.

Commonly misidentified lines
(See ICLAC register)

No commonly misidentified cell lines were used in this study.

Plants

Seed stocks

Report on the source of all seed stocks or other plant material used. If applicable, state the seed stock centre and catalogue number. If plant specimens were collected from the field, describe the collection location, date and sampling procedures.

Novel plant genotypes

Describe the methods by which all novel plant genotypes were produced. This includes those generated by transgenic approaches, gene editing, chemical/radiation-based mutagenesis and hybridization. For transgenic lines, describe the transformation method, the number of independent lines analyzed and the generation upon which experiments were performed. For gene-edited lines, describe the editor used, the endogenous sequence targeted for editing, the targeting guide RNA sequence (if applicable) and how the editor was applied.

Authentication

Describe any authentication procedures for each seed stock used or novel genotype generated. Describe any experiments used to assess the effect of a mutation and, where applicable, how potential secondary effects (e.g. second site T-DNA insertions, mosiacism, off-target gene editing) were examined.
